# Gene dosage imbalance disrupts systemic metabolism in the Dp16 Down syndrome mouse model

**DOI:** 10.7554/eLife.110476

**Published:** 2026-08-03

**Authors:** Fangluo Chen, Muzna Saqib, Christy M Nguyen, Dylan C Sarver, Y Eugene Yu, Susan Aja, Marcus M Seldin, G William Wong

**Affiliations:** 1 Department of Physiology, Pharmacology and Therapeutics, Johns Hopkins University, School of Medicine Baltimore United States; 2 Center for Metabolism and Obesity Research, Johns Hopkins University, School of Medicine Baltimore United States; 3 Department of Biological Chemistry, University of California, Irvine Irvine United States; 4 Center for Epigenetics and Metabolism, University of California Irvine Irvine United States; 5 The Children's Guild Foundation Down Syndrome Research Program, Department of Cancer Genetics and Genomics, Roswell Park Comprehensive Cancer Center Buffalo United States; 6 Genetics, Genomics and Bioinformatics Program, State University of New York at Buffalo Buffalo United States; 7 Department of Neuroscience, Johns Hopkins University School of Medicine Baltimore United States; Yale University United States; University of Colorado Anschutz Medical Campus United States

**Keywords:** down syndrome, obesity, diabetes, insulin resistance, dyslipidemia, gene dosage imbalance, Mouse

## Abstract

Gene dosage imbalance resulting from an extra copy of human chromosome 21 (Hsa21) contributes to numerous clinical features in Down syndrome (DS). While dysregulated metabolism has long been noted in DS, the underlying cause is poorly understood and vastly understudied. To fill this critical knowledge gap, we conducted a comprehensive metabolic analysis of Dp(16)1Yey/+mice (abbreviated Dp16), a segmental duplication model carrying ~58% of the triplicated Hsa21 gene orthologs. Our multi-tissue transcriptomic analyses reveal shared and sex-specific increases in expression dosage of the triplicated genes in white and brown adipose tissues, liver, skeletal muscle, and hypothalamus. Despite sexual dimorphism in body weight, body temperature, food intake, and physical activity, Dp16 males and females share striking core phenotypes of pronounced insulin resistance, glucose intolerance, impaired lipid clearance, and dyslipidemia. Functional assessments, combined with biochemical, transcriptomic, and metabolomic analyses reveal tissue signatures of immune activation and a pro-inflammatory state, ER and oxidative stress, fibrosis, impaired glucose and fatty acid catabolism, altered lipid and bile acid profiles, and reduced mitochondrial respiratory capacity in Dp16 mice. These concerted changes disrupt homeostatic mechanisms that underpin metabolic health, contributing to systemic metabolic dysfunction. An obesogenic diet further exacerbates insulin resistance in Dp16 males and females despite divergent weight gain. The collective phenotypes broadly reflect the metabolic profile of DS. Our extensive molecular, biochemical, and physiological data provide an essential foundation for genetic dissection of dosage-sensitive genes affecting glucose and lipid metabolism, and for testing therapeutic strategies to improve metabolic outcomes in DS.

## Introduction

Down syndrome (DS) is the most common aneuploidy compatible with postnatal survival and it affects ~1/700 live births (Sherman et al., 2007; Antonarakis et al., 2020). The presence of an extra human chromosome 21 (Hsa21) in DS alters the expression dosage of a large number of triplicated genes, which has major functional impacts across many organ systems (Zhu et al., 2018; Korenberg et al., 1994; Antonarakis, 2017; Duchon et al., 2021; Korbel et al., 2009). The core clinical features of DS include cognitive deficits, craniofacial dysmorphology, hypotonia, and the development of Alzheimer (AD)-like pathology in mid-life (Antonarakis et al., 2020; LaCombe and Roper, 2020). With variable degrees of penetrance and expressivity, individuals with DS also frequently exhibit congenital heart defects, hearing and vision loss, leukemia, reduced bone mass, and gastrointestinal diseases (Antonarakis et al., 2020; LaCombe and Roper, 2020).

In the last few decades, DS research has largely been centered on understanding how trisomy 21 affects brain development and its consequences on learning and cognitive function (Haydar and Reeves, 2012). However, a major clinical feature that has frequently been overlooked and neglected, although increasingly appreciated, is that adolescents and adults with DS also have a much higher incidence of obesity, insulin resistance, type 2 diabetes, and dyslipidemia (Van Goor et al., 1997; Bertapelli et al., 2016; Fonseca et al., 2005; Aslam et al., 2022; Valentini et al., 2017; Buonuomo et al., 2016; Adelekan et al., 2012; Gross et al., 2019; Garcia-de la Puente et al., 2021; de la Piedra et al., 2017). A recent large retrospective study in the UK, spanning three decades, found that the median age at diabetes diagnosis was 15 years earlier in individuals with DS and diabetes was more than four times more common in children and young adults with DS than in individuals without DS (Aslam et al., 2022). There was also an increased incidence of obesity in children and young adults with DS, with rates increasing over time (Aslam et al., 2022). Despite the fact that metabolic dysfunction in DS was first noted in the 1960s (Milunsky and Neurath, 1968) and well documented (Van Goor et al., 1997; Bertapelli et al., 2016; Fonseca et al., 2005; Milunsky and Neurath, 1968; Real de Asua et al., 2014), the underlying cause is largely unknown.

Beyond clinical and epidemiological observations (Van Goor et al., 1997; Bertapelli et al., 2016; Fonseca et al., 2005; Aslam et al., 2022), only limited studies have been conducted to determine the mechanistic underpinnings of DS-associated metabolic dysfunction. Most human studies involving adolescents or adults with DS assessed the impact of trisomy 21 on food intake, adiposity, physical activity level, and energy expenditure in adolescents or adults with DS (Hill et al., 2013; González-Agüero et al., 2011; Fernhall et al., 2005; Ptomey et al., 2020; Allison et al., 1995; Gutierrez-Hervas et al., 2020; Magenis et al., 2018; Fox et al., 2019; Luke et al., 1996), with one study documenting a deficit in mitochondrial function in the skeletal muscle (Phillips et al., 2013). However, the relative contribution of genetics versus lifestyle to altered metabolic parameters seen in DS remains challenging to untangle. At the cellular level, altered mitochondrial morphology, dynamics, and function have been well documented in vitro in cultured cells derived from DS (Helguera et al., 2013; Valenti et al., 2010; Valenti et al., 2011; Panagaki et al., 2019; Parra et al., 2018; Xu et al., 2022; Mollo et al., 2021; Anderson et al., 2021; Piccoli et al., 2013; Izzo et al., 2014; Izzo et al., 2017); it is unclear, however, whether this translates into changes in systemic metabolism in vivo.

To fill this critical knowledge gap, we have recently conducted a comprehensive and in-depth analysis of changes in systemic metabolism in two trisomic DS mouse models (Ts65Dn and TcMAC21; Sarver et al., 2023a; Sarver et al., 2023b). Ts65Dn mice were the workhorse of DS models for over two decades (Reeves et al., 1995). Due to a translocation event between mouse chromosome 16 (Mmu16) and Mmu17, Ts65Dn mice carry a freely segregating marker chromosome, Ts(17^16^), that contains ~59% of the gene orthologs found on Hsa21 (Reeves et al., 1995; Davisson et al., 1990; Davisson et al., 1993); they lack ~70 Hsa21 gene orthologs (Gupta et al., 2016). Under the basal state, chow-fed Ts65Dn mice of both sexes are glucose intolerant (Sarver et al., 2023b). Deterioration in metabolic homeostasis becomes much more apparent when mice are challenged with a high-fat diet (HFD). While obese Ts65Dn mice of both sexes exhibit dyslipidemia, male mice also show impaired systemic insulin sensitivity, reduced mitochondrial activity, and elevated fibrotic and inflammatory gene signatures in the liver and adipose tissue. Our systems-level analysis also reveals major changes in gene connectivity and pathways in liver and adipose tissues that contribute to dysregulated glucose and lipid metabolism seen in Ts65Dn mice (Sarver et al., 2023b). The metabolic phenotypes of Ts65Dn mice are largely consistent with the clinical and epidemiological findings of DS, namely the proclivity of individuals with DS to develop diabetes (Van Goor et al., 1997; Bertapelli et al., 2016; Fonseca et al., 2005; Aslam et al., 2022), dyslipidemia (Adelekan et al., 2012; Garcia-de la Puente et al., 2021; de la Piedra et al., 2017; Magge et al., 2019), and reduced mitochondrial function (Helguera et al., 2013). However, Ts65Dn has one major limitation. In addition to the 103 triplicated Hsa21 gene orthologs, Ts65Dn mice also carry an additional 41 triplicated protein-coding genes (from the sub-centromeric region of Mmu17) unrelated to Hsa21 (Reinholdt et al., 2011; Duchon et al., 2011). This confounds and complicates the genotype-phenotype correlations in this mouse model (Duchon et al., 2022; Guedj et al., 2023).

TcMAC21 is a recently generated transchromosomic mouse model carrying a non-mosaic Hsa21q (Kazuki et al., 2020). Due to multiple deletions, TcMAC21 mice are missing ~7% of the Hsa21 genes located on the long arm. Our systematic analysis of TcMAC21 mice has led to completely unexpected findings–the TcMAC21 mice are hypermetabolic, showing a dramatic increase in mitochondrial respiration and energy expenditure, and are markedly leaner despite consuming greater amounts of food, and are strikingly more insulin sensitive (Sarver et al., 2023a). These phenotypes are inconsistent with the clinical profile of DS. Thus, TcMAC21 mice do not model the metabolic phenotypes seen in DS. This phenomenon may be partly caused by abnormal interactions between human and mouse proteins, including the orthologous proteins of these two species, as well as the presence of over 400 non-coding human genes with uncertain effects on the mouse transcriptome (Sarver et al., 2023a).

To overcome the caveats and limitations associated with the metabolic studies of Ts65Dn and TcMAC21, and to help inform the selection of mouse model that best reflects the metabolic profile of DS, we undertook a comprehensive metabolic analysis of Dp(16)1Yey/+(abbreviated Dp16), another widely used mouse model of DS (Li et al., 2007). The Dp16 mice carry a duplicated segment of Mmu16 syntenic to Hsa21, with 115 triplicated Hsa21 gene orthologs (Li et al., 2007). Unlike the Ts65Dn mice generated from chromosomal translocation (Davisson et al., 1990; Davisson et al., 1993), the Dp16 mice were generated by chromosomal engineering based on precise Cre/LoxP-mediated recombineering (Li et al., 2007) and therefore carry no extra non-Hsa21 gene orthologs. Also, unlike the TcMAC21 mice, all the triplicated Hsa21 gene orthologs in Dp16 mice are of mouse origin. The Dp16 mouse model thus allows us to address the contribution of gene dosage imbalance to changes in systemic metabolism.

While we observed striking sex differences in various metabolic parameters, Dp16 male and female mice also shared important metabolic deficits that are hallmarks of dysregulated systemic metabolism; both sexes developed pronounced glucose intolerance, insulin resistance, impaired lipid clearance, and dyslipidemia, and these phenotypes were further exacerbated by a HFD. Using a multi-omics approach, we showed that Dp16 mice displayed transcriptomic, metabolomic, and biochemical signatures associated with tissue fibrosis, oxidative stress, a pro-inflammatory state, impaired metabolism and mitochondrial function, and dyslipidemia. Our pathway enrichment analyses revealed global changes in gene expression and biological pathways across major metabolic tissues in Dp16 mice. These collective changes underpin and contribute to the metabolic dysfunction seen in Dp16 mice. Collectively, our data suggest that dosage imbalance arising from the triplication of Hsa21 gene orthologs, along with its cascading effects across tissue transcriptomes and metabolomes, are causally linked to insulin resistance and impaired systemic glucose and lipid metabolism. Our present study lays critical and essential groundwork for genetic dissection of dosage-sensitive genes underpinning widespread metabolic deficits in DS.

## Results

### Increased gene expression dosage of the triplicated Hsa21 gene orthologs across major metabolic tissues

**Figure 1.**
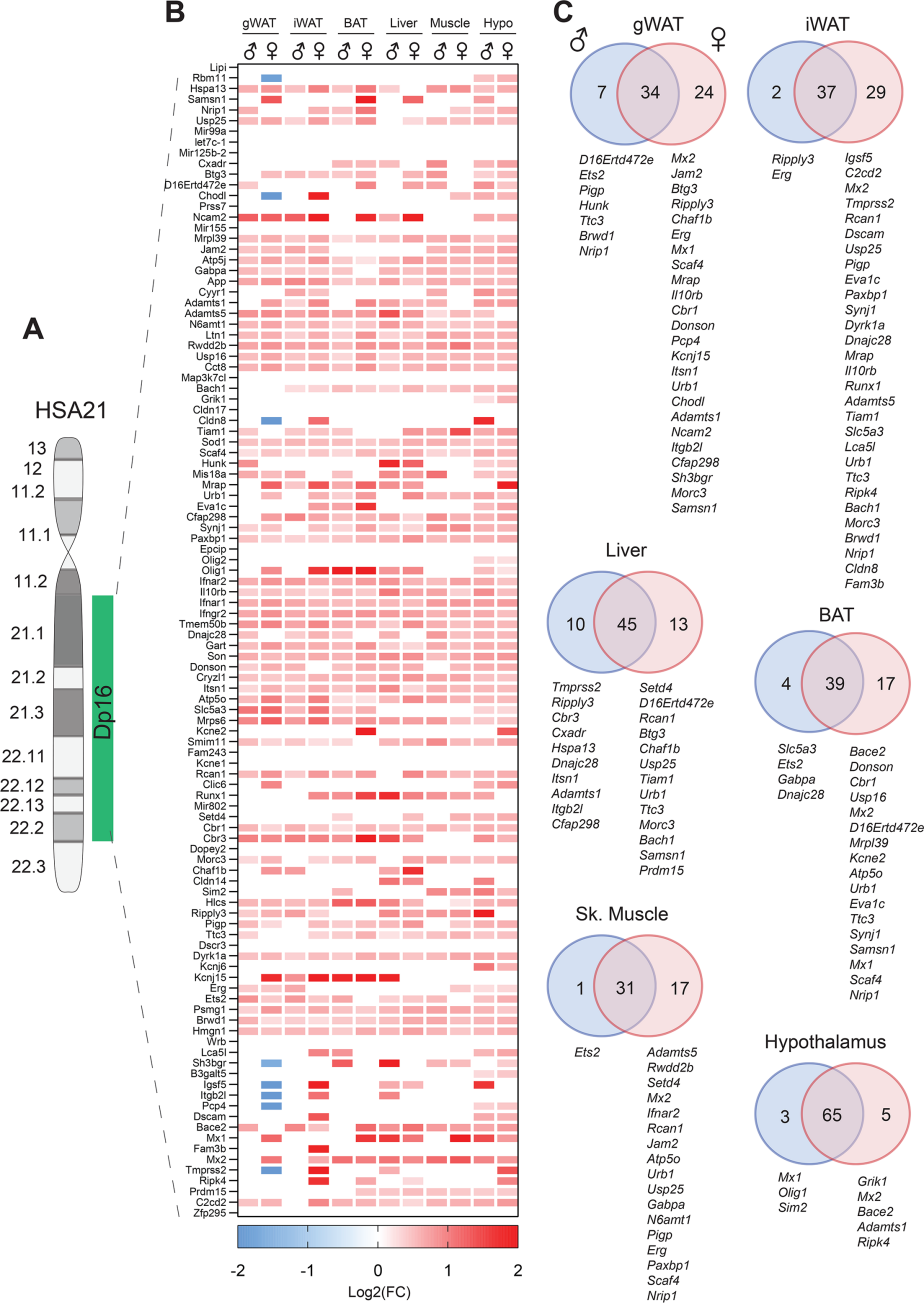
Increased gene expression dosage of the triplicated Hsa21 gene orthologs on mouse chromosome 16 (Mmu16) across tissues. (**A**) Graphical representation of human chromosome 21 (Hsa21) and the syntenic Mmu16 segment that is duplicated in Dp16 mice. (**B**) Global view of the expression of 108 triplicated Hsa21 gene orthologs on Mmu16 in gonadal white adipose tissue (gWAT), inguinal white adipose tissue (iWAT), interscapular brown adipose tissue (BAT), skeletal muscle (gastrocnemius), and hypothalamus. Red denotes transcript that is expressed at >1.5-fold the WT level, whereas blue denotes transcript that is expressed at significantly lower level compared to WT control. The Ktrap gene cluster (23 Ktrap genes) located between Cldn8 and Tiam1 is not shown. (**C**) Overlap analysis showing differentially expressed Hsa21 gene orthologs that are shared between males and females across six tissues. The criteria for differentially expressed genes (DEGs) is log2(FC)>0 with padj <0.05. n=6 RNA samples per genotype per sex per tissue-type. Chow-fed WT and Dp16 mice were at 27.5 weeks of age at the time of tissue collection.

The Dp16 mice are triplicated for ~58% of Hsa21 gene orthologs (Li et al., 2007; Lana-Elola et al., 2016; Figure 1A). First, we wanted to establish which among the 115 triplicated Hsa21 gene orthologs on Mmu16 are expressed in six major metabolic tissues: gonadal white adipose tissue (gWAT; visceral fat), inguinal white adipose tissue (iWAT; subcutaneous fat), brown adipose tissue (BAT), skeletal muscle (gastrocnemius), and hypothalamus. Bulk RNA-sequencing showed that the majority of the triplicated genes are indeed expressed at the expected higher dosage (1.5-fold or higher) across tissues and their expression are regulated in a tissue- and sex-specific manner (Figure 1B and C). Interestingly, female gWAT is the only tissue with eight triplicated Hsa21 gene orthologs (*Rbm11*, *Chodl*, *Cldn8*, *Sh3bgr*, *Igsf5*, *Itgb2l*, *Pcp4* and *Tmprss2*) whose expressions are significantly suppressed relative to WT controls (Figure 1B). Except female gWAT, we observed little evidence of dosage compensation, consistent with recent findings (Hunter et al., 2023). Of the six tissues examined, hypothalamus expresses the largest number of triplicated Hsa21 gene orthologs, as well as the largest number of shared triplicated genes between males and females (Figure 1C). Visceral fat (gWAT) and skeletal muscle (gastrocnemius) have the lowest number of shared differentially expressed triplicated genes between sexes, with females expressing a significantly higher number of triplicated genes compared to males (Figure 1C). Together, these data indicate increased expression dosage of many triplicated Hsa21 gene orthologs across major metabolic tissues in Dp16 mice, with sex differences noted.

### Sexual dimorphism in body weight, food intake, physical activity, and body temperature in Dp16 mice

**Figure 2.**
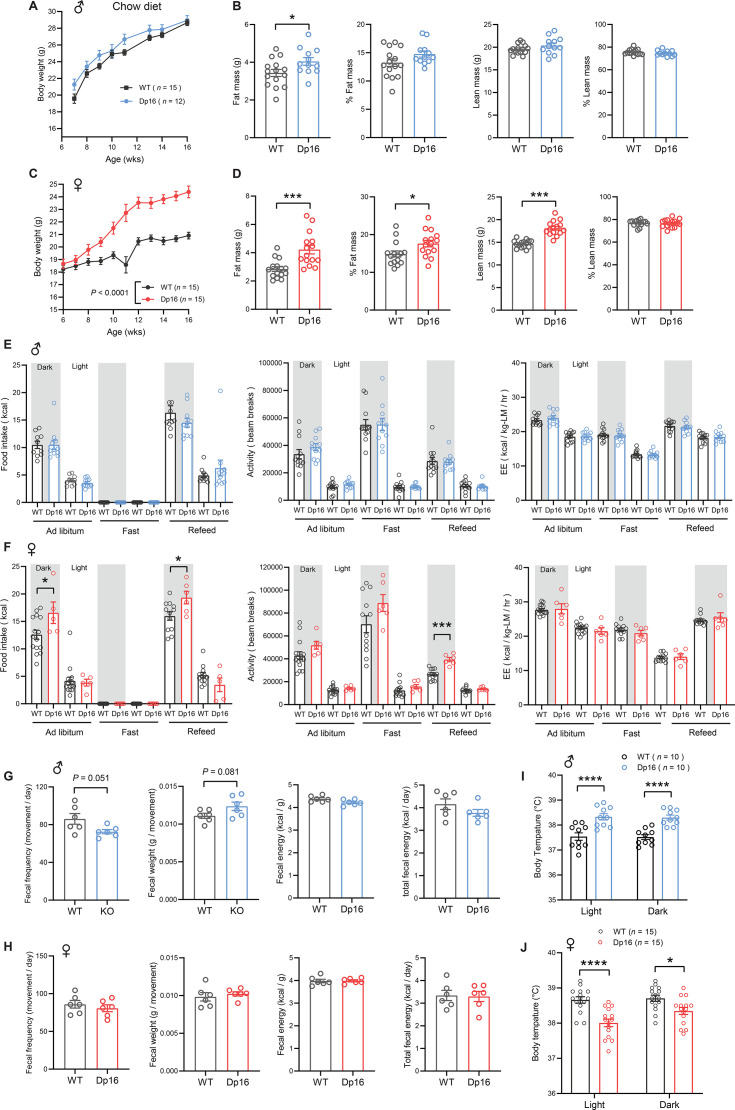
Sexual dimorphism in body weight, body temperature, food intake, and physical activity in chow-fed Dp16 mice. (**A**) Body weight of chow-fed male Dp16 and WT mice over time. (**B**) Absolute and relative (% of body weight) fat and lean mass in male mice (WT = 15; Dp16 = 12). (**C**) Body weight of chow-fed female Dp16 and WT mice over time. (**D**) Absolute and relative (% of body weight) fat and lean mass in female mice (WT = 15; Dp16 = 15). (**E–F**) Food intake, total physical activity level, and energy expenditure of male (**E**) and female (**F**) Dp16 and WT mice across the circadian cycle (light and dark) and metabolic states (ad libitum fed, fast, refeed). Sample size for male (WT = 10–12; Dp16 = 11–12) and female (WT = 13–15; Dp16 = 5–6) mice. (**G–H**) Fecal frequency, average fecal weight, and fecal energy content (per gram and total) in male (**G**) and female (**H**) Dp16 and WT mice. Sample size for male (WT = 6; Dp16 = 6) and female (WT = 6; Dp16 = 6) mice. (**I–J**) Body temperature in the light and dark cycle of male (**I**) and female (**J**) Dp16 and WT mice. Sample size for male (WT = 10; Dp16 = 10) and female (WT = 15; Dp16 = 15) mice. All data are presented as mean ± SEM. * p<0.05; *** p<0.001; **** p<0.0001. For body weight over time, data were analyzed by 2-way ANOVA with Sidek post hoc tests.

We next determined the impact of gene dosage imbalance on systemic metabolism under the basal state when mice were fed a standard chow. The body weights of Dp16 male mice from 7 to 16 weeks old were not different from WT controls (Figure 2A). Body composition analysis by NMR revealed a modest increase in fat mass in Dp16 male mice but no difference in lean mass (Figure 2B). At 27.5 weeks of age, body weights and the absolute and relative (% of body weight) weights of visceral (gWAT) and subcutaneous (iWAT) fat, liver, and heart were not different between genotypes in male mice (Figure 2—figure supplement 1). The absolute and relative weights of the kidney, however, were significantly higher in Dp16 male mice. Female body weights were similar between genotypes from 6 to 8 weeks of age; however, Dp16 females gained markedly more weight compared to WT controls from 9 weeks of age onward (Figure 2C). Increased body weight in Dp16 females was due to increased fat and lean mass (Figure 2D). At 27.5 weeks of age, body weights and the absolute weights of gWAT and iWAT were significantly higher in Dp16 females compared to WT controls (Figure 2—figure supplement 1). The absolute and relative weights of liver, heart, and kidney were not different between genotypes in female mice.

**Figure 2—figure supplement 1.**
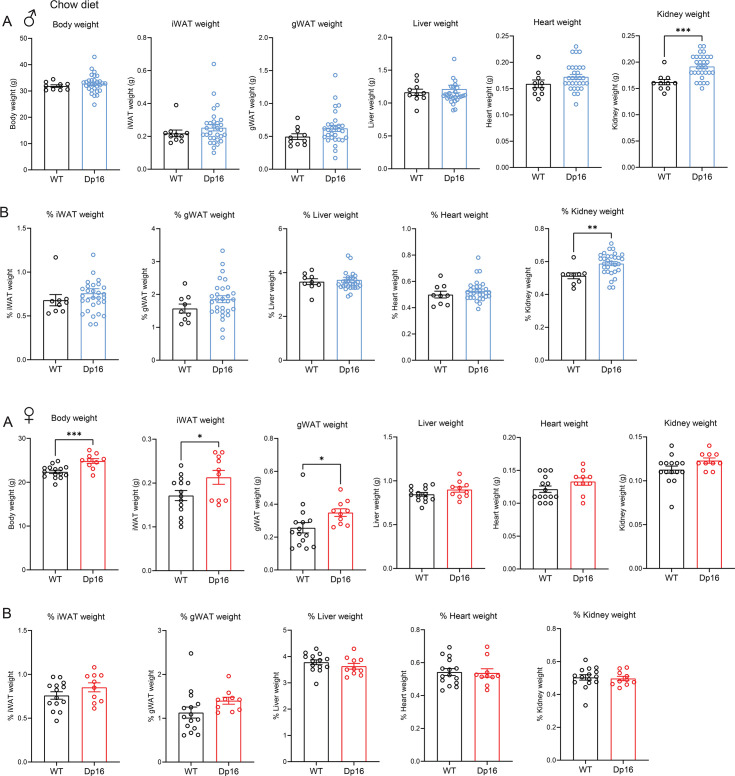
Body and tissue weights of chow-fed male and female mice at termination of study. Tissues were collected from chow-fed male and female mice at 27.5 weeks of age. Body weights and the absolute (**A and C**) and relative (B and D; % of body weight) weights of gWAT, iWAT, liver, and kidney in Dp16 and WT male (**A–B**) and female (**C–D**) mice. gWAT, gonadal white adipose tissue; iWAT, inguinal white adipose tissue. Sample size: WT male = 10; Dp16 male = 30; WT female = 15; Dp16 female = 10. All data are presented as mean ± SEM. * p<0.05; ** p<0.01; *** p<0.001.

**Figure 2—figure supplement 2.**
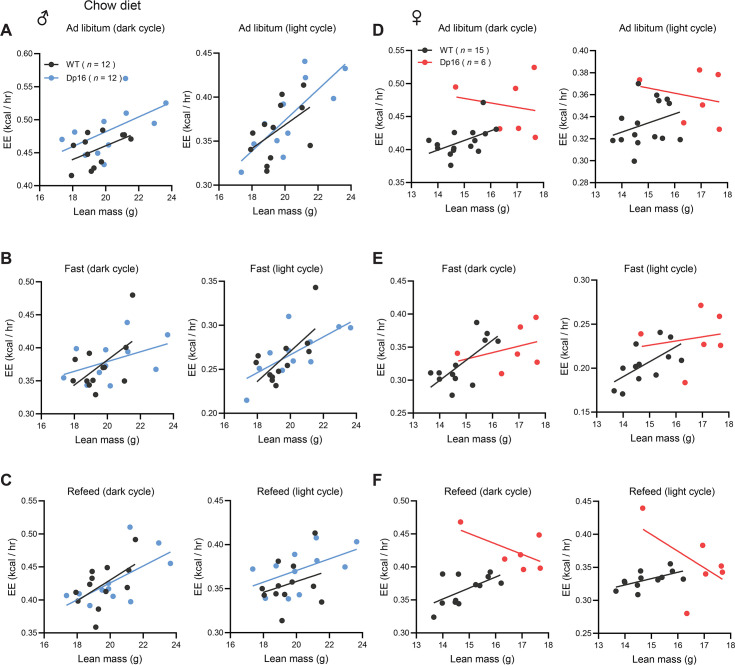
ANCOVA analysis of energy expenditure in chow-fed mice where lean mass is used as a covariate. ANCOVA analysis of WT and Dp16 male mice across the circadian cycle (dark and light) in ad libitum fed (**A**), fasted (**B**), and refed (**C**) states. ANCOVA analysis of WT and Dp16 female mice across the circadian cycle (dark and light) in ad libitum fed (**D**), fasted (**E**), and refed (**F**) states. Male Sample size: WT = 12; Dp16 = 12. Female sample size: WT = 15; Dp16 = 6.

**Figure 2—figure supplement 3.**
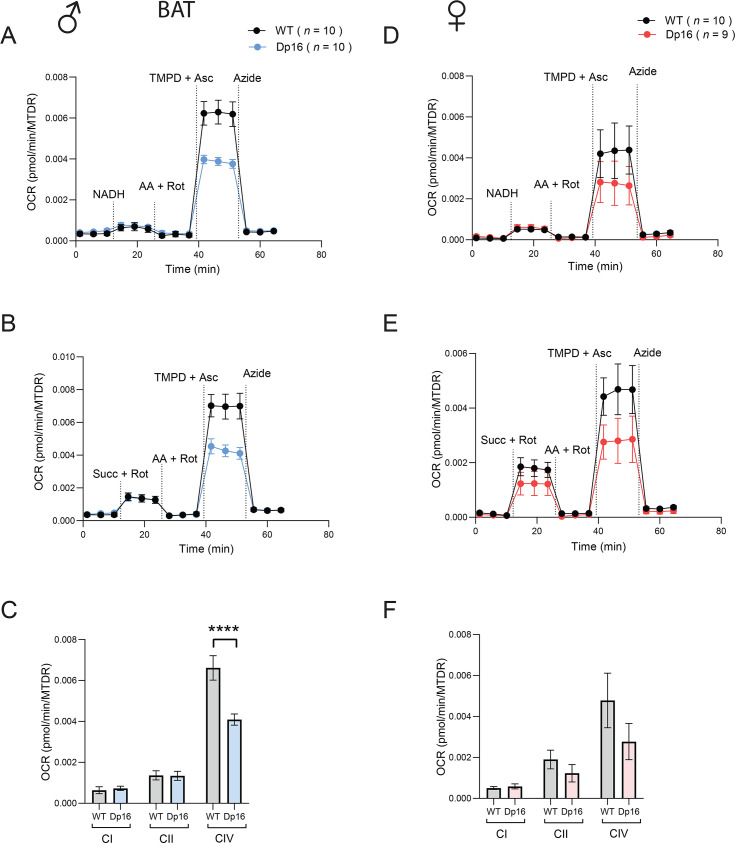
Reduced mitochondrial activity in the brown adipose tissue (BAT) of Dp16 mice. Mitochondrial respiration through complex I (CI), CII, and CIV in BAT of WT and Dp16 male and female mice fed a standard chow. (**A and D**) Average oxygen consumption rate (OCR) traces per group, normalized to mitochondrial content. Each group tracing represents the average trace of 10 WT and 9–10 Dp16 samples. Each tracing shows the entire process of the Seahorse-based respirometry assay with injection compounds listed at the time of introduction to the sample. The sequence is as follows: (i) basal reads, (ii) addition of NADH (activation of respiration through complex I), (iii) addition of antimycin A (AA, inhibitor of complex III) and rotenone (Rot, inhibitor of complex I), (iv) addition of TMPD and ascorbate (to activate complex IV via electron donation to cytochrome c), and finally (v) addition of azide (inhibitor of complex IV). (**B and E**) The same information as presented in (**A and D**) conducted on the same samples, but the NADH injection step is replaced with the injection of succinate (to activate respiration through complex II) and rotenone (to inhibit complex I). (**C and F**) Average values of all data presented for BAT. Each data point represents the average of three technical replicates measured at three separate times. Both independent measurements of complex IV (CIV) were used to determine average CIV respiration. **** p<0.0001 (two-way ANOVA with Sidak’s multiple comparison).

**Figure 2—figure supplement 4.**
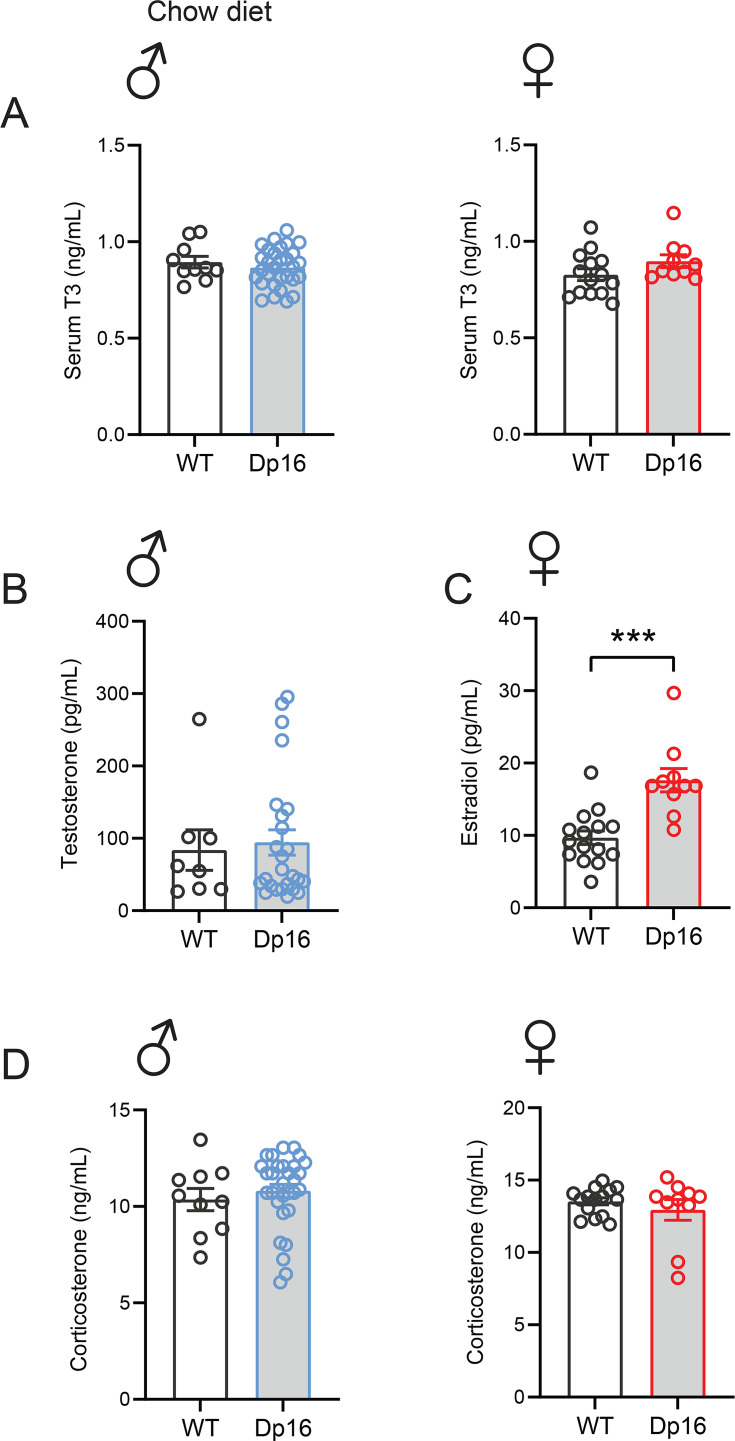
Serum Triiodothyronine (T3), sex, and stress hormone levels in WT and Dp16 mice fed a standard chow. (**A**) Serum T3 levels in male and female mice. (**B**) Serum testosterone levels in male mice. (**C**) Serum estradiol levels in female mice. (**D**) Serum corticosterone in male and female mice. Sample size: male WT = 8–10; male Dp16 = 25–30; female WT = 14–15; female Dp16 = 10.

We performed indirect calorimetry analysis to determine food intake, physical activity level, metabolic rate (VO_2_), and energy expenditure across the circadian cycle (light and dark phase) and metabolic states (ad libitum fed, fasted, and refed). None of the parameters measured were different between genotypes in male mice (Figure 2E). In females, however, food intake was significantly higher in Dp16 mice compared to WT controls in the ad libitum fed state and during the refeeding period following a fast (Figure 2F). Physical activity levels were also higher in Dp16 females during the refed period. Energy expenditure (normalized to lean mass), however, was not different between Dp16 females and WT controls across the circadian cycles and metabolic states (Figure 2F). Normalization of energy expenditure to lean mass can lead to an overestimation of energy expenditure (Tschöp et al., 2012). We therefore also performed ANCOVA analyses (using lean mass as a covariate of energy expenditure) (Tschöp et al., 2012). Both types of analyses indicated no differences in energy expenditure between genotypes of either sex across the circadian cycles and metabolic states (Figure 2—figure supplement 2).

To account for any potential differences in nutrient absorption in the intestine, we measured fecal output, frequency, and weight, as well as the fecal energy content by fecal bomb calorimetry. None of the fecal parameters were significantly different between genotypes of either sex (Figure 2G–H). Interestingly, Dp16 males had higher body temperature compared to WT controls in both the light and dark cycle, whereas Dp16 females had lower body temperature (Figure 2I–J). This prompted us to assess whether Dp16 mice have altered mitochondrial function in BAT, a major thermogenic tissue. Despite differences in body temperature, both Dp16 males and females had reduced maximal mitochondrial respiratory capacity in BAT, an effect more pronounced in males (Figure 2—figure supplement 3). Circulating Triiodothyronine (T3) levels, a hormone that also controls body temperature, were not different between genotypes of either sex (Figure 2—figure supplement 4). We measured serum levels of sex and stress hormones, as these could contribute to sex differences in metabolic outcomes. Corticosterone levels were not different between genotypes of either sex. Testosterone levels in males were variable and not significantly different between genotypes, whereas estradiol levels were higher in Dp16 females (Figure 2—figure supplement 4), possibly contributing to greater physical activity (Krause et al., 2021; Correa et al., 2015) and lower body temperature (Zhang et al., 2020; Krajewski-Hall et al., 2018). Our results suggest that lower body temperature, coupled with higher food intake, likely contributes to greater weight gain over time in Dp16 females. Together, these data reveal striking sex differences in body weight, food intake, physical activity, and body temperature in Dp16 mice.

### Glucose intolerance, insulin resistance, impaired lipid clearance, and dyslipidemia in Dp16 mice

**Figure 3.**
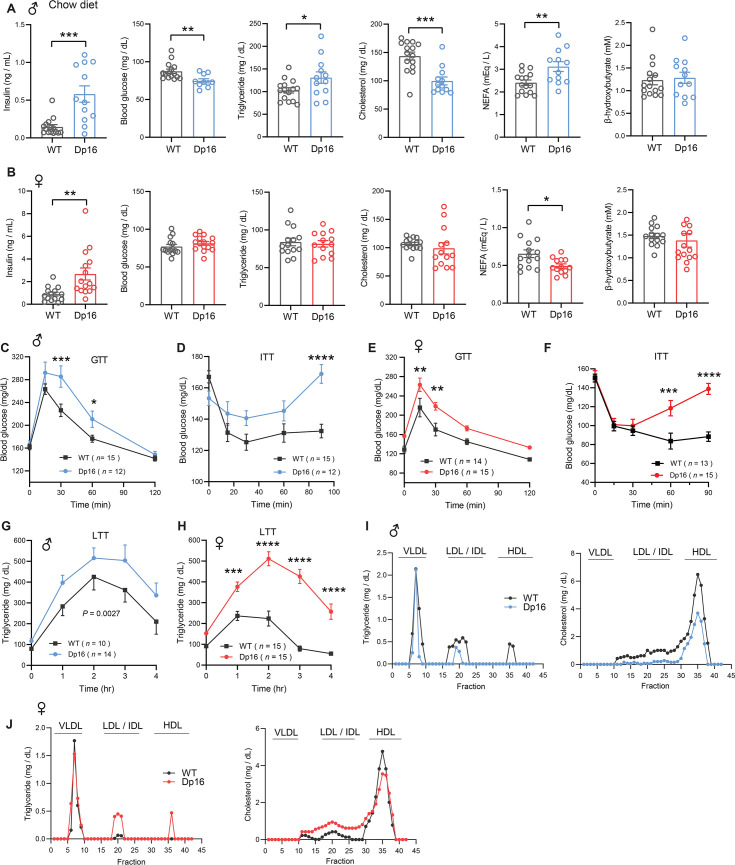
Glucose intolerance, insulin resistance, and impaired lipid clearance in chow-fed Dp16 mice. (**A–B**) Overnight fasting insulin, blood glucose, serum triglyceride, cholesterol, non-esterified free fatty acids (NEFA), and β-hydroxybutyrate (ketone) in male (**A**) and female (**B**) Dp16 and WT mice. Sample size for male mice (WT = 15; Dp16 = 12) and female mice (WT = 14; Dp16 = 15). (**C–F**) Impaired glucose tolerance as determined by the glucose tolerance test (GTT) in male (**C**) and female (**E**) Dp16 mice compared to WT controls. Impaired insulin sensitivity as determined by the insulin tolerance test (ITT) in male (**D**) and female (**F**) Dp16 compared to WT controls. Sample size for male mice (WT = 15; Dp16 = 12) and female mice (WT = 14; Dp16 = 15). (**G–H**) Impaired triglyceride clearance in response to lipid gavage as determined by the lipid tolerance test (LTT) in male (**G**) and female (**H**) Dp16 relative to WT controls. Sample size for male mice (WT = 10; Dp16 = 14) and female mice (WT = 15; Dp16 = 15). (**I–J**) Pooled mouse sera from male (**I**) and female (**J**) Dp16 and WT mice were fractionated by fast protein liquid chromatography (FPLC), and the triglyceride and cholesterol content of each fraction was quantified. Fractions corresponding to very-low density lipoprotein (VLDL), low-density lipoprotein (LDL), intermediate-density lipoprotein (IDL), and high-density lipoprotein (HDL) are indicated. All data are presented as mean ± SEM. * p<0.05; ** p<0.01; *** p<0.001; **** p<0.0001. For all tolerance tests, data were analyzed by two-way ANOVA with Sidek post hoc tests.

Type 2 diabetes and altered lipid profile are well documented in DS population (Van Goor et al., 1997; Bertapelli et al., 2016; Fonseca et al., 2005; Aslam et al., 2022; Valentini et al., 2017; Buonuomo et al., 2016; Adelekan et al., 2012; Gross et al., 2019; Garcia-de la Puente et al., 2021; de la Piedra et al., 2017). To determine baseline insulin, glucose, and lipid profiles, we measured blood glucose, serum insulin, triglyceride (TG), cholesterol, non-esterified free fatty acids (NEFA), and β-hydroxybutyrate (BHB; ketone) in overnight fasted (16 hr) mice. The Dp16 males fed a standard chow had significantly higher fasting insulin levels (Figure 3A). Fasting hyperinsulinemia likely contributed to lower fasting blood glucose seen in Dp16 male mice. Serum TG and NEFA levels were also significantly higher in Dp16 males compared to WT controls, suggesting enhanced fat mobilization in the fasted state. Serum β-hydroxybutyrate (ketone) levels were not different between genotypes whereas serum cholesterol levels were markedly lower in Dp16 male mice (Figure 3A). Like the males, Dp16 females also had significantly higher fasting serum insulin levels (Figure 3B). Unlike the males, blood glucose, TGs, cholesterol, and ketone levels were not different between genotypes in females. Also, different from the males, Dp16 females had lower NEFA levels relative to WT controls.

**Figure 3—figure supplement 1.**
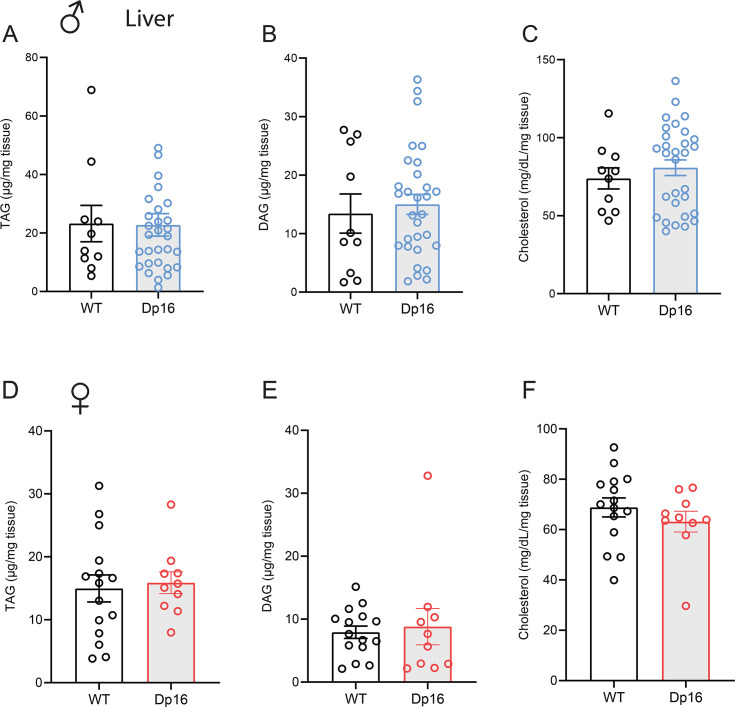
Liver triacylglycerol (TAG), diacylglycerol (DAG), and cholesterol levels in chow-fed Dp16 mice. Quantification of hepatic TAG and DAG (by TLC method), and cholesterol (by infinity assay kit) levels in chow-fed Dp16 male (**A–C**) and female mice (**D–F**) and their corresponding WT controls. Sample size: male WT = 10 and Dp16 = 30; female WT = 15 and Dp16 = 10.

**Figure 3—figure supplement 2.**
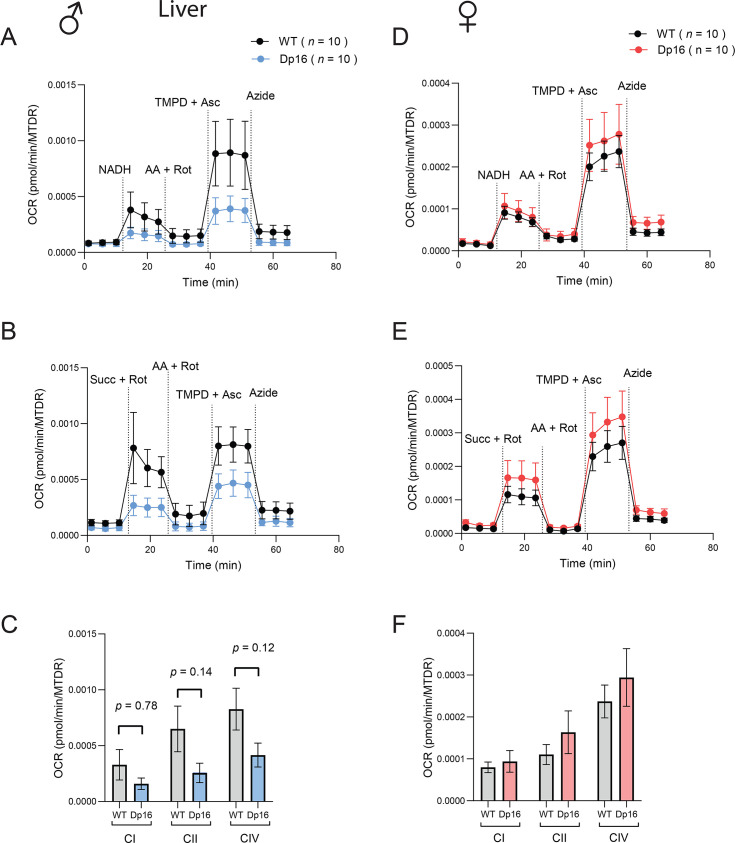
Mitochondrial activity in the liver of Dp16 mice. Mitochondrial respiration through complex I (CI), CII, and CIV in the liver of WT and Dp16 male and female mice fed a standard chow. (**A and D**) Average oxygen consumption rate (OCR) traces per group, normalized to mitochondrial content. Each group tracing represents the average trace of 10 WT and 10 Dp16 samples. Each tracing shows the entire process of the Seahorse-based respirometry assay with injection compounds listed at the time of introduction to the sample. The sequence is as follows: (i) basal reads, (ii) addition of NADH (activation of respiration through complex I), (iii) addition of antimycin A (AA, inhibitor of complex III) and rotenone (Rot, inhibitor of complex I), (iv) addition of TMPD and ascorbate (to activate complex IV via electron donation to cytochrome c), and finally (v) addition of azide (inhibitor of complex IV). (**B and E**) The same information as presented in (**A and D**) conducted on the same samples, but the NADH injection step is replaced with the injection of succinate (to activate respiration through complex II) and rotenone (to inhibit complex I). (**C and F**) Average values of all data presented for liver. Each data point represents the average of three technical replicates measured at three separate times. Both independent measurements of complex IV (CIV) were used to determine average CIV respiration.

Higher fasting insulin levels in Dp16 males and females suggest insulin resistance. To further assess glucose metabolism in these mice, we performed glucose and insulin tolerance tests (ITTs) to determine the rate of glucose clearance in response to glucose or insulin injection. Both Dp16 males and females showed impaired glucose clearance after glucose loading (Figure 3C and E). To confirm that Dp16 mice have impaired insulin action, we directly assessed insulin sensitivity via ITT. The rate of glucose clearance in response to insulin injection was markedly impaired in both Dp16 males and females (Figure 3D and F). Impaired insulin action, glucose intolerance, and fasting hyperinsulinemia strongly indicate an insulin resistance phenotype in Dp16 mice.

We next assessed whether Dp16 mice have altered lipid handling capacity by performing a lipid tolerance test. The rate of TG clearance in response to an acute lipid load was significantly impaired in Dp16 mice of either sex, with Dp16 females showing a much more striking deficit in lipid clearance (Figure 3G and H). To determine whether Dp16 mice have altered lipoprotein profiles, we subjected pooled sera to FPLC fractionation followed by the quantification of TG and cholesterol levels in each fraction. Dp16 males had lower TG and cholesterol levels in the LDL/IDL and HDL fractions (Figure 3I). In contrast, Dp16 females had higher TG and cholesterol levels in the LDL/IDL fractions, as well as higher TG levels in the HDL fractions. Since liver is a key organ in lipid and lipoprotein synthesis and export, we measured hepatic TG, diacylglycerol (DAG), and cholesterol contents. No genotypic differences in either sex was observed (Figure 3—figure supplement 1). Dp16 males had lower maximal liver mitochondrial respiratory capacity, though not significant; and this was not different between genotypes in females (Figure 3—figure supplement 2). Altogether, our data indicate that Dp16 mice of either sex developed pronounced insulin resistance, glucose intolerance, dyslipidemia, and impaired lipid clearance.

### Altered hepatic and serum metabolome in Dp16 mice

**Figure 4.**
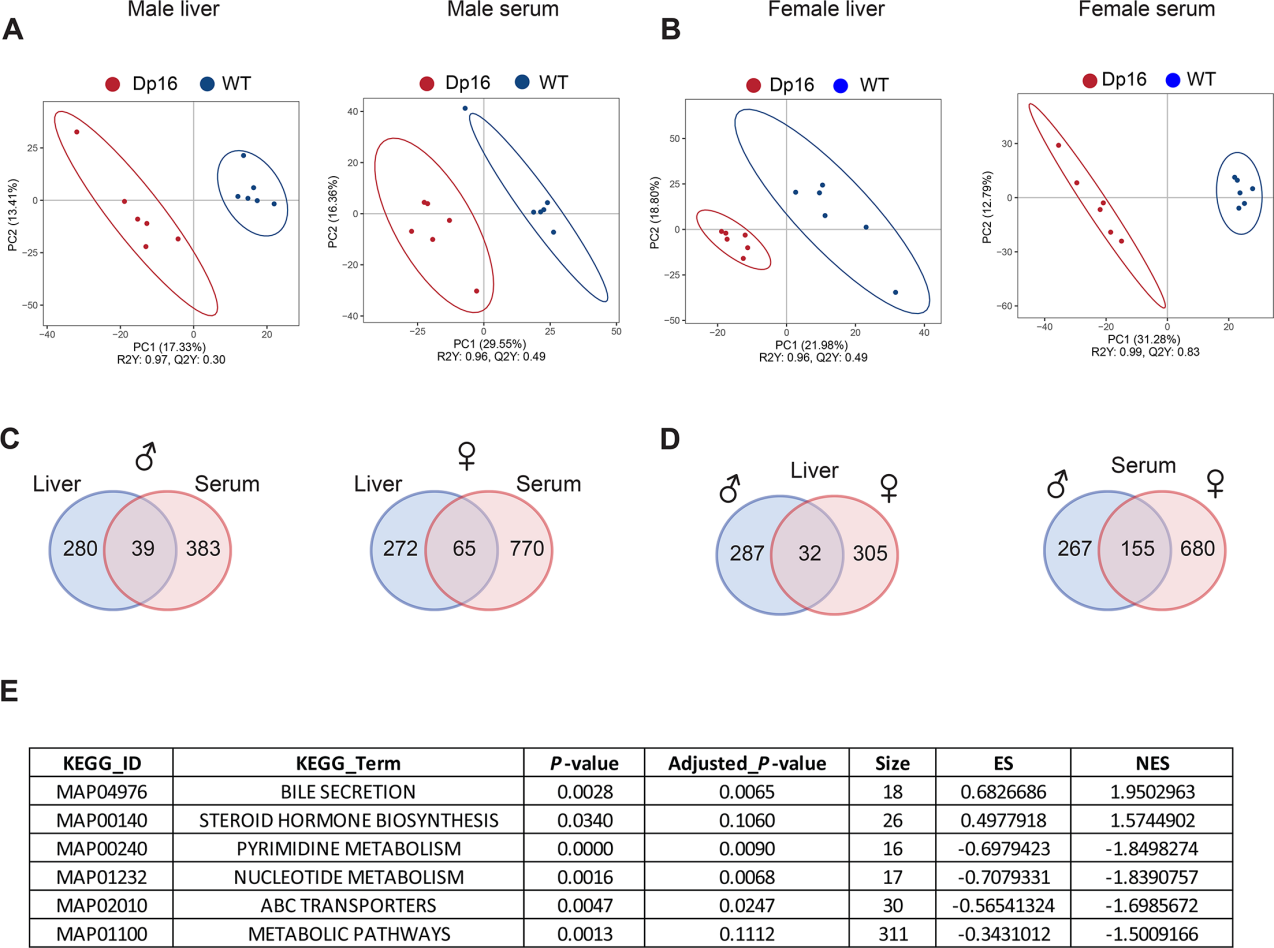
Altered liver and serum metabolome in Dp16 male and female mice. (**A–B**) Partial least squares discrimination analysis (PLS-DA) of liver and serum metabolites of Dp16 and WT males and females. N=6 samples per genotype per sex. (**C**) Venn diagram of differential metabolites shared between liver and serum in Dp16 male or female mice. (**D**) Venn diagram of differential liver or serum metabolites shared between Dp16 males and females. (**E**) KEGG enrichment showing altered metabolic processes in Dp16 female serum. ES, enrichment score; NES, normalized enrichment score. Figure 4—source data 1.Differential metabolites in Dp16 male mouse liver vs WT controls.Differential metabolites criteria: VIP >1.0, fold change (FC)>1.2 or FC <0.833 and p-value <0.05. Sample name notation: male WT liver (M_WT_L), male WT serum (M_WT_S), male Dp16 liver (M_16_L), male Dp16 serum (M_16_S), female WT liver (F_WT_L), female WT serum (F_WT_L), female Dp16 liver (F_16_L), female Dp16 serum (F_16_S). Figure 4—source data 2.Differential metabolites in Dp16 female mouse liver vs WT controls.Differential metabolites criteria: VIP >1.0, fold change (FC)>1.2 or FC <0.833 and p-value <0.05. Sample name notation: male WT liver (M_WT_L), male WT serum (M_WT_S), male Dp16 liver (M_16_L), male Dp16 serum (M_16_S), female WT liver (F_WT_L), female WT serum (F_WT_L), female Dp16 liver (F_16_L), female Dp16 serum (F_16_S). Figure 4—source data 3.Differential metabolites in Dp16 male mouse serum vs WT controls.Differential metabolites criteria: VIP >1.0, fold change (FC)>1.2 or FC <0.833 and *<*i>P-value <0.05. Sample name notation: male WT liver (M_WT_L), male WT serum (M_WT_S), male Dp16 liver (M_16_L), male Dp16 serum (M_16_S), female WT liver (F_WT_L), female WT serum (F_WT_L), female Dp16 liver (F_16_L), female Dp16 serum (F_16_S). Figure 4—source data 4.Differentially expressed metabolites in Dp16 female mouse serum vs WT controls.Differential metabolites criteria: VIP >1.0, fold change (FC)>1.2 or FC <0.833 and *<*i>P-value <0.05. Sample name notation: male WT liver (M_WT_L), male WT serum (M_WT_S), male Dp16 liver (M_16_L), male Dp16 serum (M_16_S), female WT liver (F_WT_L), female WT serum (F_WT_L), female Dp16 liver (F_16_L), female Dp16 serum (F_16_S). Figure 4—source data 5.Shared and distinct differential metabolites in Dp16 male and female mouse liver and serum vs WT controls.Sample name notation: male WT liver (M_WT_L), male WT serum (M_WT_S), male Dp16 liver (M_16_L), male Dp16 serum (M_16_S), female WT liver (F_WT_L), female WT serum (F_WT_L), female Dp16 liver (F_16_L), female Dp16 serum (F_16_S).

Since Dp16 mice showed profound disturbances in systemic energy metabolism, we performed untargeted metabolomic analyses to assess possible changes in liver and serum metabolome. A total of 4182 metabolites were identified from the 48 serum and liver samples. Partial Least Square Discriminant Analysis (PLS-DA) indicated that liver and serum metabolome of Dp16 males and females are clearly distinguishable from that of their corresponding WT controls (Figure 4A and B). In male and female liver, we observed 319 and 337 differential metabolites, respectively, in Dp16 mice vs WT controls (Figure 4—figure supplement 1; Figure 4—source data 1–4). In Dp16 male and female serum samples, we observed 422 and 835 differential metabolites, respectively (Figure 4—figure supplement 1). When comparing the differential metabolites found in liver and serum, there appeared to be limited overlap between the two compartments (Figure 4C). Major sex differences were seen in the differential metabolites found in liver and serum. There were 32 differential metabolites shared between Dp16 male and female liver, and 155 differential serum metabolites shared between the sexes (Figure 4D). The majority of differential metabolites found in liver and serum, however, were not shared between the sexes (Figure 4—source data 5).

**Figure 4—figure supplement 1.**
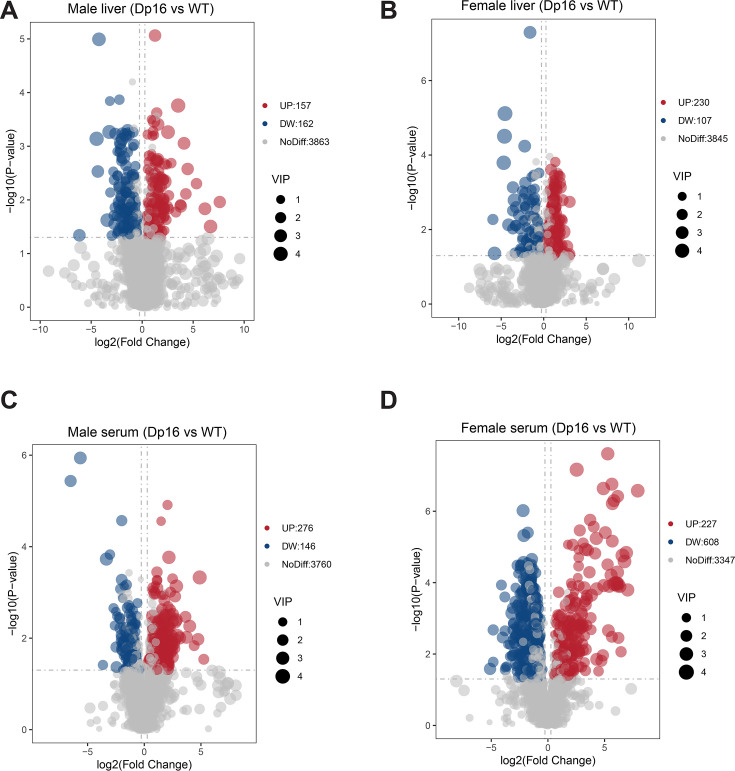
Differential metabolites found in the liver and serum of Dp16 male and female mice. Volcano plots showing differential metabolites up- and down-regulated in Dp16 male liver (**A**), female liver (**B**), male serum (**C**), and female serum (**D**). n=6 per genotype. VIP, Variable Importance in Projection. VIP scores provide a quantitative measure of a metabolite’s discriminatory power between different groups. Metabolites with a VIP score of 1.0 or greater are considered significant.

**Figure 4—figure supplement 2.**
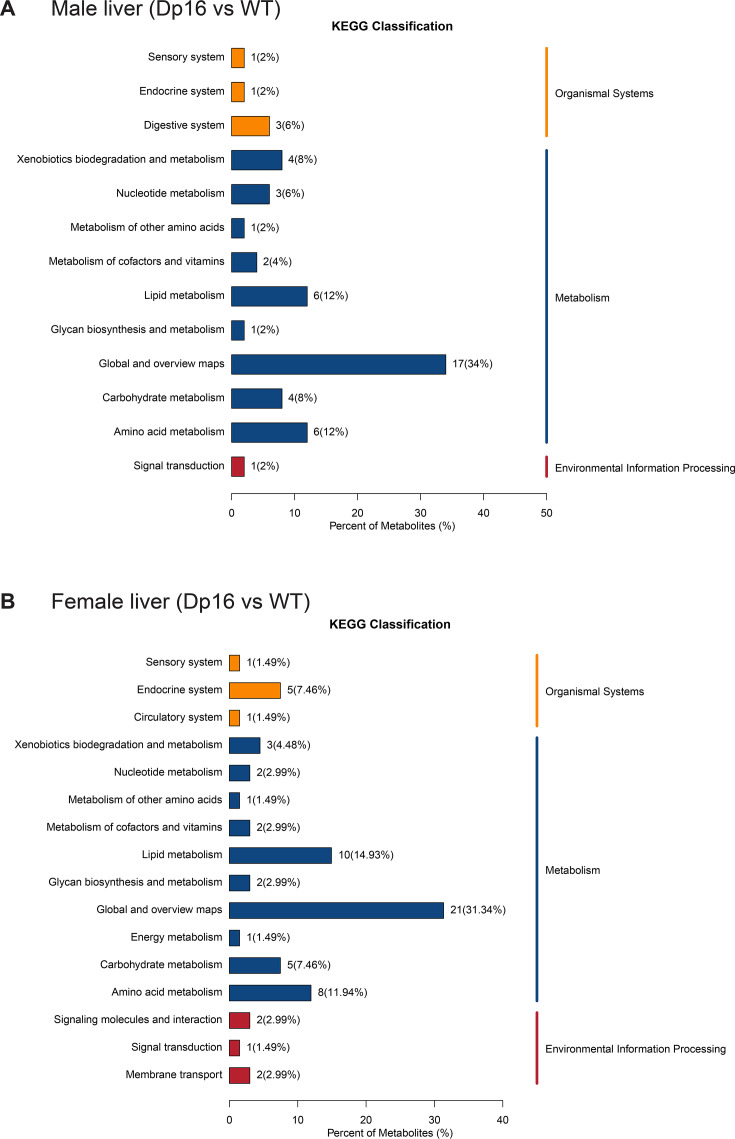
KEGG classification analysis of liver metabolites. KEGG classification plots based on the differential metabolites from male (**A**) and female (**B**) Dp16 mouse liver vs WT control. The horizontal coordinates in the graph indicate the number of metabolites annotated under a particular KEGG pathway as a percentage of the number of all annotated metabolites, the vertical coordinates are KEGG pathway primary classifications on the right and KEGG pathway secondary classifications on the left.

**Figure 4—figure supplement 3.**
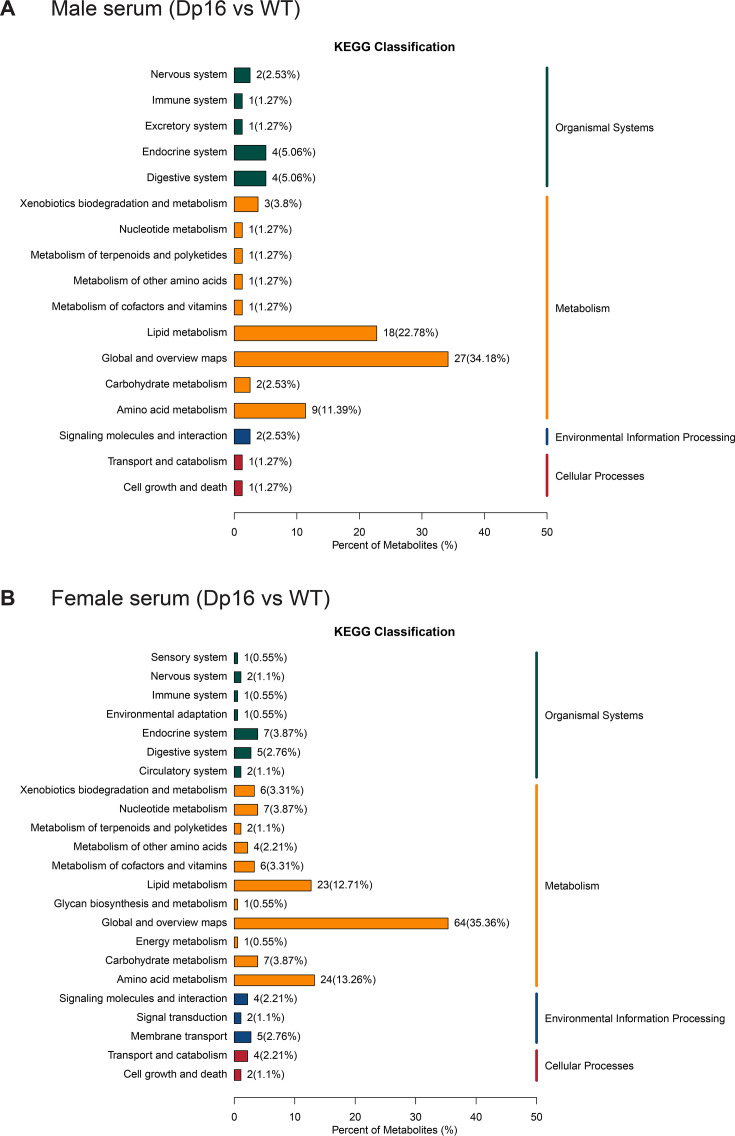
KEGG classification analysis of serum metabolites. KEGG classification plots based on the differential metabolites from male (**A**) and female (**B**) Dp16 mouse liver vs WT control. The horizontal coordinates in the graph indicate the number of metabolites annotated under a particular KEGG pathway as a percentage of the number of all annotated metabolites, the vertical coordinates are KEGG pathway primary classifications on the right and KEGG pathway secondary classifications on the left.

**Figure 4—figure supplement 4.**
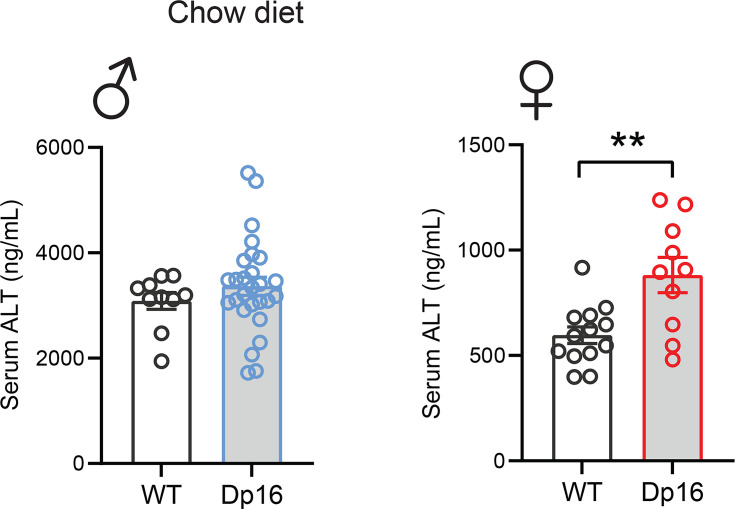
Serum alanine aminotransferase (ALT) levels in WT and Dp16 mice fed a standard chow. Serum ALT levels in male and female mice. Sample size: male WT = v10; male Dp16=27; female WT = v14; female Dp16=10.

As shown by Kyoto Encyclopedia of Genes and Genomes (KEGG) classification, differential metabolites related to global, as well as lipid and amino acid, metabolism accounted for the major differences seen in liver and serum of Dp16 males and females (Figure 4—figure supplements 2 and 3). In Dp16 female serum, KEGG enrichment analysis indicated altered metabolic pathways related to bile secretion, steroid hormone biosynthesis, nucleotide metabolism, and ABC transporters (Figure 4E). In female and male liver, as well as male serum, KEGG enrichment analyses did not yield any pathways with a false discovery rate less than 0.05.

To provide greater detail, we highlighted some of the differential metabolites found in Dp16 male and female mice (Tables 1 and 2). Consistent with recent findings (Dunn et al., 2026), we also observed changes in hepatic bile acids content in both sexes, with most of the bile acids showing a reduced level in Dp16 mice. In contrast to the liver, we observed a more extensive changes in circulating bile acids, all of them except two were elevated in Dp16 mice of both sexes. Many bile acids serve as ligands for nuclear hormone receptors (e.g. FXR and TGR5) that control various aspects of glucose and lipid metabolism (Fuchs et al., 2025; Perino et al., 2021), and extensive changes in circulating bile acids may potentially contribute, at least in part, to the systemic metabolic phenotypes in Dp16 mice. In addition to bile acids, circulating levels of many phospholipids (e.g. LysoPC, LysoPA, LysoPE), some with signaling roles (O’Donnell et al., 2018), were also altered. In both liver and serum, multiple acyl-carnitines (intermediates in fat oxidation) were elevated in liver and serum, suggesting impaired fatty acid catabolism in Dp16 mice; this phenotype is further supported by our transcriptomic data indicating reduced expression of fat oxidation genes in liver and BAT (data are discussed further below).

**Table 1.** Selective differential metabolites in the liver and serum of Dp16 male mice. Metabolites are considered significantly different if fold change (FC)>1.2 or<0.833, p-value <0.05, and the variable importance in projection (VIP) score is >1. Sample size: WT (n=6) and Dp16 (n=6).

Name	Class	log2FC	p-value	VIP	Up.Down
**Liver**					
Chenodeoxycholylmethionine	Bile acids	0.906	0.0003	1.23	Up
23-Nordeoxycholic acid	Bile acids	–0.958	0.0066	1.08	Down
Taurolithocholate sulfate	Bile acids	–0.796	0.0490	2.11	Down
7 a,12a-Dihydroxy-cholestene-3-one (DHCHO)	Cholestane steroids	3.771	0.0123	2.17	Up
Undecanedioylcarnitine	Acylcarnitine	1.016	0.0217	1.68	Up
(6E)-Tridec-6-enedioylcarnitine	Acylcarnitine	0.803	0.0303	1.88	Up
(2E,5Z,7E)-Decatrienoylcarnitine	Acylcarnitine	–2.315	0.0212	1.05	Down
(9Z,11E,13Z)-Octadeca-9,11,13-trienoylcarnitine	Acylcarnitine	–1.277	0.0349	1.44	Down
Icosadienoic acid	Fatty acids and conjugates	2.030	0.0042	2.80	Up
12-HHTrE	Fatty acids and conjugates	–0.679	0.0402	1.56	Down
LysoPC(18:4(6Z,9Z,12Z,15Z)/0:0)	Glycerophosphocholines	0.994	0.0214	1.48	Up
Lipoyllysine	Lipoamides	–1.226	0.0440	1.54	Down
all-trans-4-Oxoretinoic acid	Retinoids	–1.065	0.0254	1.88	Down
Sphingosine (d17:1)	Amines	2.643	0.0148	1.70	Up
**Serum**					
hyocholic acid	Bile acids	2.493	0.0030	3.06	Up
Taurolithocholate sulfate	Bile acids	1.516	0.0109	3.29	Up
Apocholic acid	Bile acids	1.837	0.0173	3.29	Up
Methyl cholate	Bile acids	1.417	0.0182	3.29	Up
Taurochenodeoxycholic acid (TCDCA)	Bile acids	2.049	0.0195	2.55	Up
Tauro-omega-muricholic acid	Bile acids	1.597	0.0201	2.91	Up
(3b,5b,7a,12a)–3,7,12-trihydroxy-Cholan-24-oic acid	Bile acids	2.315	0.0209	2.50	Up
Glycohyocholic acid (GHCA)	Bile acids	1.709	0.0210	3.26	Up
3beta-Glycocholic acid	Bile acids	1.764	0.0256	3.01	Up
7-Ketodeoxycholic acid (7-keto DCA)	Bile acids	1.635	0.0340	2.51	Up
6,7-Diketolithocholic acid (6,7-diketo LCA)	Bile acids	2.171	0.0400	2.18	Up
7,12-diketolithocholic acid (7,12-diketo LCA)	Bile acids	2.068	0.0414	1.75	Up
Glycoursodeoxycholic acid (GUDCA)	Bile acids	–1.308	0.0403	1.60	Down
23-Nordeoxycholic acid (23-nor- DCA)	Bile acids	–1.190	0.0032	1.18	Down
6,15-diketo-13,14-dihydro Prostaglandin F1alpha	Eicosanoids	1.173	0.0008	2.20	Up
Prostaglandin B1	Eicosanoids	2.094	0.0020	1.89	Up
Prostaglandin D2	Eicosanoids	1.157	0.0041	1.28	Up
11-Dehydro-thromboxane B2	Eicosanoids	1.181	0.0116	1.08	Up
8-Isoprostaglandin F2a	Eicosanoids	0.916	0.0237	1.10	Up
Non-7-enoylcarnitine	Acyl-carnitine	–1.563	0.0007	1.79	Down
3,6-Dihydroxydecanoylcarnitine	Acyl-carnitine	–2.095	0.0008	1.83	Down
(6E)-Tridec-6-enedioylcarnitine	Acyl-carnitine	0.553	0.0221	1.16	Up
trans-2-Dodecenoylcarnitine	Acyl-carnitine	0.501	0.0276	1.24	Up
O-dodecanedioylcarnitine	Acyl-carnitine	0.663	0.0263	1.46	Up
7-Keto-dehydroepiandrosterone	Androstane steroids	1.122	0.0005	1.70	Up
Testosterone	Androstane steroids	1.984	0.0081	2.99	Up
Dehydroepiandrosterone (DHEA)	Androstane steroids	2.470	0.0256	3.12	Up
5Alpha-Androstan-17-Beta-Ol-3-One (DHT)	Androstane steroids	1.544	0.0368	3.48	Up
19-Hydroxyandrost-4-ene-3,17-dione	Androstane steroids	1.781	0.0404	2.10	Up
Androstenedione	Androstane steroids	0.441	0.0486	1.49	Up
FAHFA(22:6(4Z,7Z,10Z,13Z,16Z,19Z)/14-O-22:6(4Z,7Z,10Z,13Z,16Z,19Z))	Fatty acids and conjugates	–1.123	0.0305	1.99	Down
PC(20:4(6Z,8E,10E,14Z)–2OH(5 S,12R)/2:0)	Phospholipid	2.498	0.0005	1.75	Up
LysoPC(22:4(7Z,10Z,13Z,16Z)/0:0)	Phospholipid	0.766	0.0008	1.89	Up
LysoPC(18:4(6Z,9Z,12Z,15Z)/0:0)	Phospholipid	1.590	0.0138	3.04	Up
PC(MonoMe (11,3)/MonoMe (1,3))	Phospholipid	2.072	0.0228	3.07	Up
1,2-Dilauroyl-sn-glycero-3-phosphocholine	Phospholipid	–1.042	0.0055	1.31	Down
LysoPE(0:0/15:0)	Phospholipid	–1.353	0.0087	1.72	Down
1-Heptadecanoyl-glycero-3-phosphoethanolamine	Phospholipid	–0.950	0.0116	1.64	Down
LysoPE(20:5(5Z,8Z,11Z,14Z,17Z)/0:0)	Phospholipid	–1.087	0.0478	1.89	Down
19-Nordeoxycorticosterone	Hydroxysteroids	1.447	0.0043	1.01	Up
Lipoamide	Lipoamides	–1.707	0.0162	1.31	Down

**Table 2.** Selective differential metabolites in the liver and serum of Dp16 female mice. Metabolites are considered significantly different if fold change (FC)>1.2 or<0.833, p-value <0.05, and the variable importance in projection (VIP) score is >1. Sample size: WT (n=6) and Dp16 (n=6).

Name	Class	log2FC	p-Value	VIP	Up.Down
**Liver**					
23-Norcholic acid (23-NCA)	Bile acids	–4.557	0.000008	4.86	Down
6,7-Diketolithocholic acid	Bile acids	–2.049	0.0086	1.56	Down
3-Oxo-7-hydroxychol-4-enoic acid	Bile acids	–1.817	0.0159	1.21	Down
Apocholic acid	Bile acids	–2.577	0.0186	1.31	Down
23-Nordeoxycholic acid (23-NDCA)	Bile acids	–2.563	0.0350	2.72	Down
(3b,5b,7a,12a)–3,7,12-trihydroxy-Cholan-24-oic acid	Bile acids	–3.375	0.0425	2.05	Down
20-Hydroxy-leukotriene E4	Eicosanoids	1.214	0.0261	2.66	Up
6-Keto-prostaglandin E1	Eicosanoids	0.886	0.0282	1	Up
Prostaglandin E1	Eicosanoids	0.822	0.0356	1.56	Up
Prostaglandin A1	Eicosanoids	0.721	0.0382	1.16	Up
4-Hydroxydecanedioylcarnitine	Acyl-carnitine	1.690	0.0003	1.41	Up
3-Oxobutanoylcarnitine	Acyl-carnitine	0.788	0.0100	1.1	Up
4-Hydroxyhexanoycarnitine	Acyl-carnitine	0.701	0.0182	2.29	Up
(3E)-Glutaconylcarnitin	Acyl-carnitine	1.654	0.0204	1.27	Up
O-(17-Carboxyheptadecanoyl)carnitine	Acyl-carnitine	1.722	0.0262	1.55	Up
(6E)-Tridec-6-enedioylcarnitine	Acyl-carnitine	0.663	0.0278	1.61	Up
LysoPE(22:5(7Z,10Z,13Z,16Z,19Z)/0:0)	Phospholipid	1.792	0.0151	2.61	Up
PC(MonoMe (11,3) /MonoMe (11,3))	Phospholipid	–5.980	0.0054	2.17	Down
TG(20:3n6/O-18:0/18:3(9Z,12Z,15Z))	Triacylglycerols	1.293	0.0008	1.68	Up
**Serum**					
3-Oxo-7-hydroxychol-4-enoic acid (7-HOCA)	Bile acids	5.645	1.7E-07	2.84	Up
3beta-Glycocholic acid	Bile acids	3.727	1.8E-06	2.46	Up
Taurochenodeoxycholic acid (TCDCA)	Bile acids	5.069	4.0E-06	2.75	Up
Tauro-omega-muricholic acid	Bile acids	5.682	6.9E-06	2.72	Up
Glycohyocholic acid (GHCA)	Bile acids	3.525	1.2E-05	2.46	Up
Taurolithocholic acid (TLCA)	Bile acids	6.759	2.6E-05	3.26	Up
lithocholic acid (LCA)	Bile acids	3.436	2.8E-04	2.49	Up
Cholan-24-oic acid, 12-hydroxy-3-(sulfooxy)-, disodium salt, (3alpha,5beta,12alpha)- (9 CI)	Bile acids	1.948	9.3E-04	1.98	Up
3beta-Hydroxy-5-cholestenoic acid	Bile acids	2.044	9.0E-03	1.54	Up
3alpha,7alpha-Dihydroxy-12-oxo-5beta-cholanate	Bile acids	1.725	1.1E-02	1.46	Up
Glycochenodeoxycholate-3-sulfate (GCDCA-S)	Bile acids	1.989	1.2E-02	2.19	Up
(3b,5b,7a,12a)–3,7,12-trihydroxy-Cholan-24-oic acid	Bile acids	3.245	1.7E-02	1.47	Up
Beta-Hyodeoxycholic acid (β-HDCA)	Bile acids	3.311	2.9E-02	1.86	Up
hyocholic acid (HCA)	Bile acids	1.800	3.8E-02	1.66	Up
23-Norcholic acid (23-NCA)	Bile acids	–2.306	3.5E-03	1.82	Down
23-Nordeoxycholic acid (23-NDCA)	Bile acids	–1.923	4.0E-02	1.58	Down
Biliverdin	Bilirubins	0.885	1.9E-02	2.64	Up
5,6-Dihydroxyprostaglandin F1a	Eicosanoids	0.924	2.5E-03	1.06	Up
2-glyceryl-11,12-EET	Eicosanoids	1.295	1.1E-02	1.39	Up
11-Dehydro-thromboxane B2	Eicosanoids	1.654	3.9E-02	1.38	Up
13,14-dihydro-15-keto-PGA2	Eicosanoids	–1.304	1.4E-04	2.18	Down
15(S)-HETrE	Eicosanoids	–1.517	5.0E-04	2.63	Down
THROMBOXANE B2	Eicosanoids	–1.040	3.0E-03	1.03	Down
FAHFA(22:6(4Z,7Z,10Z,13Z,16Z,19Z)/14-O-22:6(4Z,7Z,10Z,13Z,16Z,19Z))	Fatty acids and conjugates	–2.148	2.7E-05	2.92	Down
FAHFA 38:5	Fatty acids and conjugates	–2.415	3.1E-05	1.62	Down
Arachidonic acid	Fatty acids and conjugates	–0.928	2.8E-04	2	Down
N-Palmitoyl Glutamine	Fatty acids and conjugates	–1.304	5.2E-03	1.33	Down
Resolvin D1	Fatty acids and conjugates	–0.876	9.8E-03	1.44	Down
LysoPC(22:4(7Z,10Z,13Z,16Z)/0:0)	Phospholipid	0.951	1.9E-04	1.96	Up
LysoPC(22:5(7Z,10Z,13Z,16Z,19Z)/0:0)	Phospholipid	0.762	1.0E-03	1.56	Up
LysoPC(22:5(4Z,7Z,10Z,13Z,16Z)/0:0)	Phospholipid	0.654	2.9E-03	1.28	Up
PC(MonoMe (11,3)/MonoMe (11,3))	Phospholipid	6.452	8.6E-03	1.9	Up
1-O-Palmitoyl-2-O-acetyl-sn-glycero-3-phosphorylcholine	Phospholipid	0.816	3.2E-02	1.2	Up
LysoPC(18:4(6Z,9Z,12Z,15Z)/0:0)	Phospholipid	1.148	3.7E-02	1.47	Up
LysoPA(20:3(8Z,11Z,14Z)/0:0)	Phospholipid	–0.934	4.3E-03	2.15	Down
LysoPC(0:0/18:1(9Z))	Phospholipid	–1.494	1.5E-03	1.14	Down
PC(20:3(8Z,11Z,14Z)/18:3(9Z,12Z,15Z))	Phospholipid	–0.868	7.3E-03	1.54	Down
LysoPE(P-18:1(9Z)/0:0)	Phospholipid	–1.305	9.8E-04	1.18	Down
Glycerophospho-N-Oleoyl Ethanolamine	Phospholipid	–1.189	1.7E-03	1.32	Down
1-heptadecanoyl-glycero-3-phosphoethanolamine	Phospholipid	–1.071	2.4E-03	1.57	Down
LPE(14:0)	Phospholipid	–1.001	7.4E-03	1.01	Down
LysoPE(20:3(8Z,11Z,14Z)/0:0)	Phospholipid	–0.987	9.4E-03	1.07	Down
LysoPE(20:5(5Z,8Z,11Z,14Z,17Z)/0:0)	Phospholipid	–1.067	9.9E-03	1.55	Down
Pregnenolone	Pregnane steroids	–1.031	5.7E-04	1.18	Down
17alpha-Hydroxyprogesterone	Pregnane steroids	–0.807	3.9E-03	2	Down
Dehydroepiandrosterone sulfate (DHEAS)	Sulfated steroids	–1.334	4.6E-04	1.31	Down

The levels of multiple eicosanoids, a class of lipids with pro- and anti-inflammatory roles (Dennis and Norris, 2015), were also changed in Dp16 mice. For example, several pro-inflammatory eicosanoids (e.g., 11-dehydro-thromboxane B2, prostaglandin B1, prostaglandin D2, 20-hydroxy-leukotriene E4) were elevated while some anti-inflammatory eicosanoids (e.g., 13,14-dihydro-15-keto-PGA2, 15(S)-HETrE) were reduced. In addition to eicosanoids, we observed lower levels of several fatty acids with anti-inflammatory and/or anti-diabetic roles (e.g., FAHFA, resolvin D1, all-trans-4-oxoretinoic acid) (Yore et al., 2014; Kuda et al., 2016; Serhan and Levy, 2018) and a concomitant increase in fatty acids with pro-inflammatory roles (e.g., icosadienoic acid, sphingosine) (Huang et al., 2011; Gomez-Larrauri et al., 2025). The general pro-inflammatory state in Dp16 mice, reflected by the metabolite data, parallel our transcriptomic data in BAT, liver, muscle, and hypothalamus showing a pro-inflammatory and heightened immune activation state (data are discussed further below).

We also would like to highlight that a crucial biomarker for oxidative stress, 8-Isoprostaglandin F2α (Roberts and Morrow, 2000), was significantly elevated in Dp16 male serum, whereas metabolites (e.g., lipoyllysine and lipoamide) that act as indirect antioxidants (Li et al., 2008) to maintain mitochondrial health were reduced. Further, N-palmitoyl glutamine, a recently discovered acylated amino acid that stimulates mitochondrial biogenesis and efficiency (Robbins et al., 2026), was also reduced in Dp16 female serum. Interestingly, 19-Nor-deoxycorticosterone (19-nor-DOC), a powerful mineralocorticoid hormone that increases blood pressure (Gomez-Sanchez et al., 1979; Gorsline and Morris, 1985), was elevated in Dp16 male serum, raising the possibility of altered blood pressure in these animals. In Dp16 female serum, we also observed elevated biliverdin (precursor of bilirubin), suggesting potential liver injury, which was confirmed by an increase in serum alanine transaminase (ALT) levels (Figure 4—figure supplement 4).

We showed using an ELISA method that estradiol levels are significantly higher in females and testosterone levels in males exhibited high variations (Figure 2—figure supplement 4). Our metabolite data, however, indicated that six androstane steroids, including testosterone, were significantly elevated in Dp16 male serum, whereas the precursors of female sex hormones (e.g., pregnenolone and 17α-hydroxyprogesterone) were reduced in female serum, presumably due to its greater conversion to estradiol. Given the pleiotropic systemic metabolic effects of testosterone and estradiol (Kelly and Jones, 2013; Hevener and Correa, 2025), changes in circulating sex hormones in Dp16 mice are likely contributing, at least in part, to the sex differences in metabolic phenotypes. Taken together, our metabolomic analyses reveal major sex-dependent and independent changes in liver and serum metabolites likely contributing to the systemic metabolic deficits in Dp16 mice.

### Transcriptomic signatures of immune activation, fibrosis, ER stress, and impaired metabolic processes in Dp16 mice

**Figure 5.**
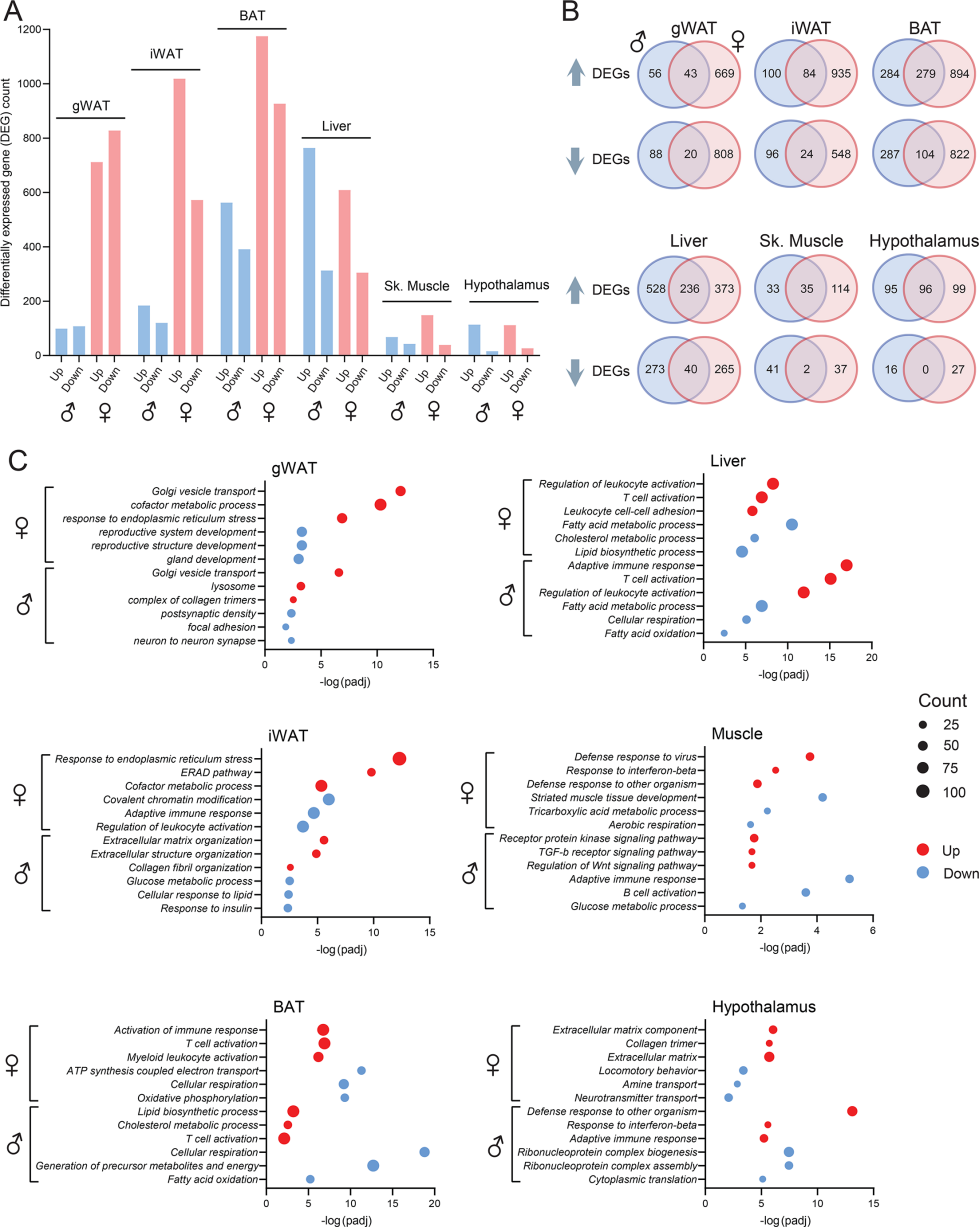
Transcriptomic changes and altered biological pathways across tissues in chow-fed Dp16 male and female mice. (**A**) Number of differentially expressed genes (DEGs) that up or down regulated across six tissues in male and female Dp16 mice and their WT littermate controls. DEG is defined as any gene with log2(FC)>0.5 and padj <0.05. N=6 per genotype per tissue. gWAT, gonadal white adipose tissue; iWAT, inguinal white adipose tissue; BAT, brown adipose tissue. (**B**) Overlap analysis showing DEGs that are shared between males and females, as well as those DEGs found in males or females only, across six tissues. (**C**) Gene ontology highlighting some of the top biological pathways altered across six tissues in male and female Dp16 mice. Figure 5—source data 1.Differentially expressed genes (DEGs) upregulated in the gonadal white adipose tissue (gWAT) of chow-fed Dp16 male mice relative to WT controls. Figure 5—source data 2.Differentially expressed genes (DEGs) down-regulated in the gonadal white adipose tissue (gWAT) of chow-fed Dp16 male mice relative to WT controls. Figure 5—source data 3.Differentially expressed genes (DEGs) upregulated in the inguinal white adipose tissue (iWAT) of chow-fed Dp16 male mice relative to WT controls. Figure 5—source data 4.Differentially expressed genes (DEGs) down-regulated in the inguinal white adipose tissue (iWAT) of chow-fed Dp16 male mice relative to WT controls. Figure 5—source data 5.Differentially expressed genes (DEGs) upregulated in the brown adipose tissue (BAT) of chow-fed Dp16 male mice relative to WT controls. Figure 5—source data 6.Differentially expressed genes (DEGs) down-regulated in the brown adipose tissue (BAT) of chow-fed Dp16 male mice relative to WT controls. Figure 5—source data 7.Differentially expressed genes (DEGs) upregulated in the liver of chow-fed Dp16 male mice relative to WT controls. Figure 5—source data 8.Differentially expressed genes (DEGs) down-regulated in the liver of chow-fed Dp16 male mice relative to WT controls. Figure 5—source data 9.Differentially expressed genes (DEGs) upregulated in the skeletal muscle (gastrocnemius) of chow-fed Dp16 male mice relative to WT controls. Figure 5—source data 10.Differentially expressed genes (DEGs) down-regulated in the skeletal muscle (gastrocnemius) of chow-fed Dp16 male mice relative to WT controls. Figure 5—source data 11.Differentially expressed genes (DEGs) upregulated in the hypothalamus of chow-fed Dp16 male mice relative to WT controls. Figure 5—source data 12.Differentially expressed genes (DEGs) down-regulated in the hypothalamus of chow-fed Dp16 male mice relative to WT controls. Figure 5—source data 13.Differentially expressed genes (DEGs) upregulated in the gonadal white adipose tissue (gWAT) of chow-fed Dp16 female mice relative to WT controls. Figure 5—source data 14.Differentially expressed genes (DEGs) down-regulated in the gonadal white adipose tissue (gWAT) of chow-fed Dp16 female mice relative to WT controls. Figure 5—source data 15.Differentially expressed genes (DEGs) upregulated in the inguinal white adipose tissue (iWAT) of chow-fed Dp16 female mice relative to WT controls. Figure 5—source data 16.Differentially expressed genes (DEGs) down-regulated in the inguinal white adipose tissue (iWAT) of chow-fed Dp16 female mice relative to WT controls. Figure 5—source data 17.Differentially expressed genes (DEGs) upregulated in the brown adipose tissue (BAT) of chow-fed Dp16 female mice relative to WT controls. Figure 5—source data 18.Differentially expressed genes (DEGs) down-regulated in the brown adipose tissue (BAT) of chow-fed Dp16 female mice relative to WT controls. Figure 5—source data 19.Differentially expressed genes (DEGs) upregulated in the liver of chow-fed Dp16 female mice relative to WT controls. Figure 5—source data 20.Differentially expressed genes (DEGs) down-regulated in the liver of chow-fed Dp16 female mice relative to WT controls. Figure 5—source data 21.Differentially expressed genes (DEGs) upregulated in the skeletal muscle (gastrocnemius) of chow-fed Dp16 female mice relative to WT controls. Figure 5—source data 22.Differentially expressed genes (DEGs) down-regulated in the skeletal muscle (gastrocnemius) of chow-fed Dp16 female mice relative to WT controls. Figure 5—source data 23.Differentially expressed genes (DEGs) upregulated in the hypothalamus of chow-fed Dp16 female mice relative to WT controls. Figure 5—source data 24.Differentially expressed genes (DEGs) down-regulated in the hypothalamus of chow-fed Dp16 female mice relative to WT controls.

To address the molecular underpinnings of the metabolic phenotypes seen in chow-fed Dp16 mice, we performed bulk RNA sequencing to assess global changes in the transcriptome and biological pathways across six metabolic tissues (Figure 5—source data 1–24). Except the liver, females have significantly more differentially expressed genes (DEGs) across tissues compared to males (Figure 5A). In Dp16 females, gWAT, iWAT, and BAT together accounted for the majority of DEGs, with the least number of DEGs seen in skeletal muscle (Figure 5A). In Dp16 males, BAT and liver have the highest number of DEGs, with skeletal muscle having the least DEGs. All tissues except gWAT have more upregulated than downregulated DEGs. Overlap analysis in males and females indicate shared DEGs across tissues, as well as DEGs that are seen only in males or females (Figure 5B). BAT and liver have the highest number of shared DEGs across sex, with skeletal muscle having the least shared DEGs. iWAT, gWAT and BAT have the highest number of DEGs that are female-specific, whereas liver and BAT having the highest number of DEGs that are male-specific (Figure 5B).

**Figure 5—figure supplement 1.**
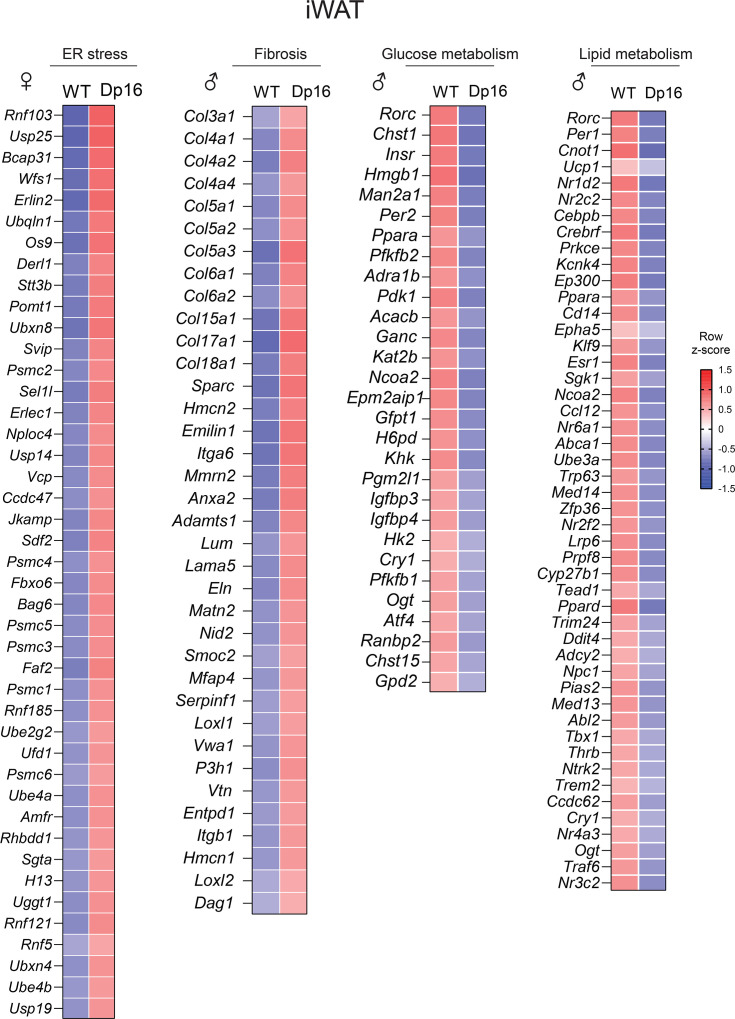
Differentially expressed genes (DEGs) involved in ER stress, fibrosis, glucose and lipid metabolism that are up- or down-regulated in the inguinal white adipose tissue (iWAT) of Dp16 mice.

**Figure 5—figure supplement 2.**
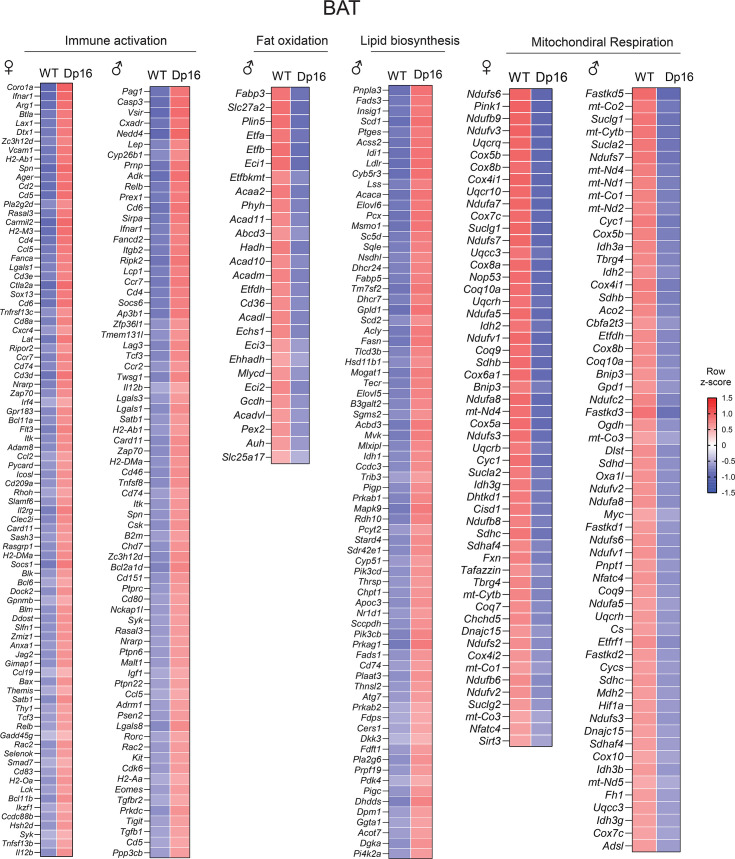
Differentially expressed genes (DEGs) involved in immune activation, lipid metabolism, and mitochondrial respiration that are up- or down-regulated in the brown adipose tissue (BAT) of Dp16 mice.

**Figure 5—figure supplement 3.**
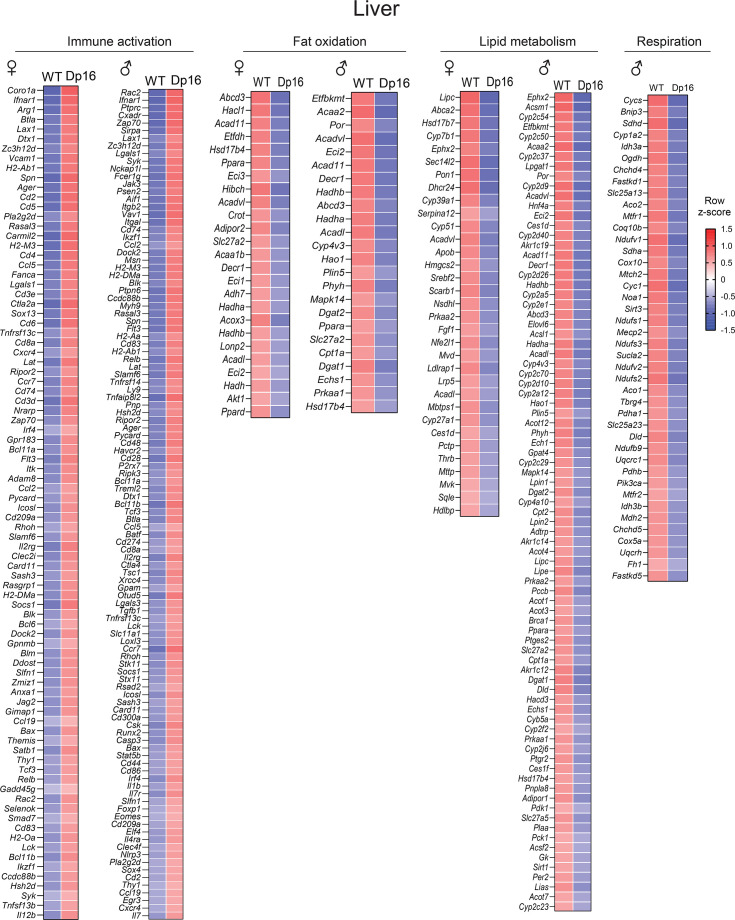
Differentially expressed genes (DEGs) involved in immune activation, lipid metabolism, and mitochondrial respiration that are up- or down-regulated in the liver of Dp16 mice.

**Figure 5—figure supplement 4.**
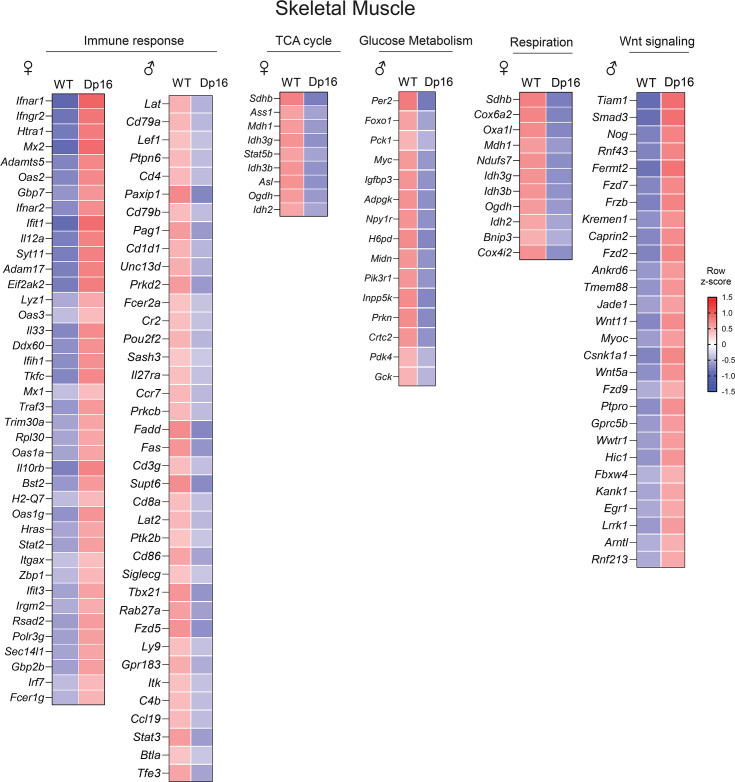
Differentially expressed genes (DEGs) involved in immune response, metabolism, mitochondrial respiration, and Wnt signaling that are up- or down-regulated in the skeletal muscle (gastrocnemius) of Dp16 mice.

**Figure 5—figure supplement 5.**
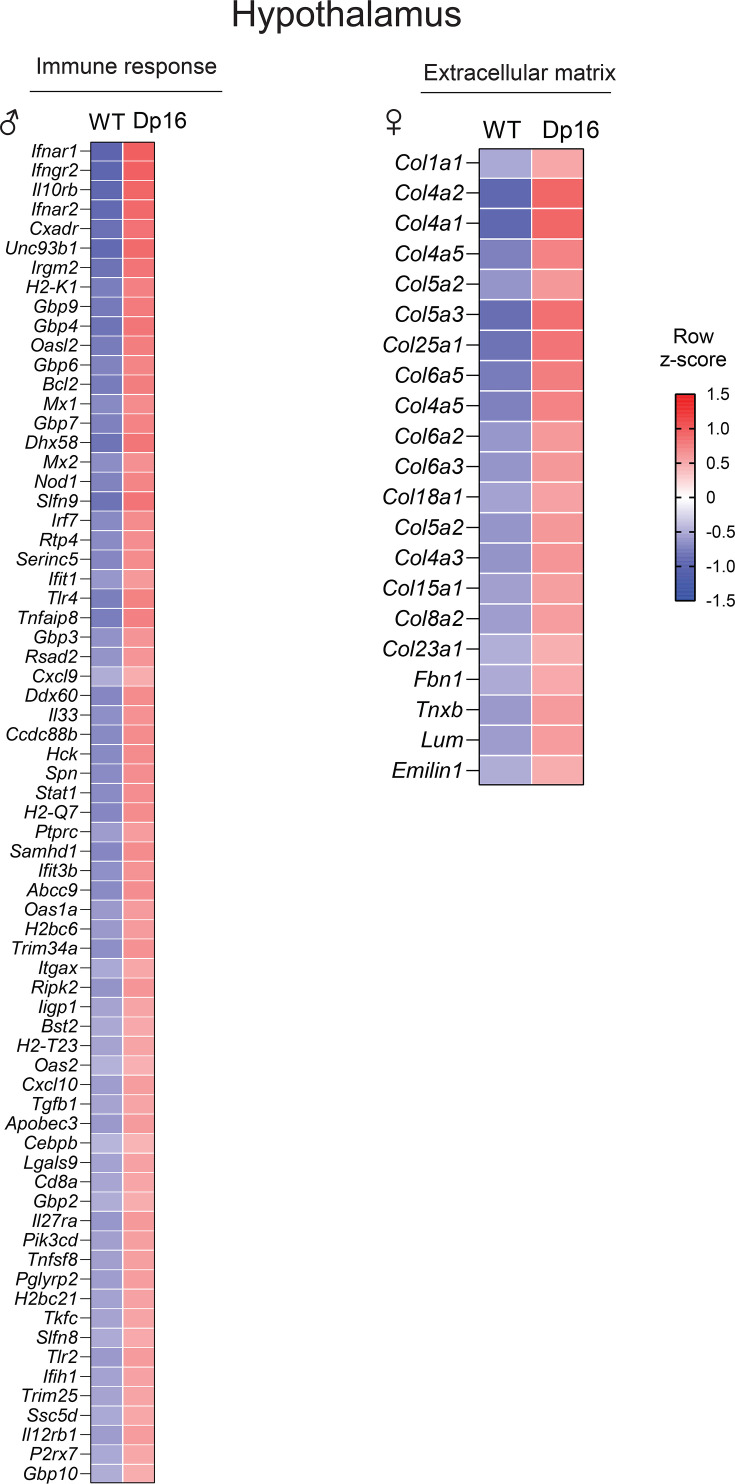
Differentially expressed genes (DEGs) involved in immune response and extracellular matrix that are upregulated in the hypothalamus of Dp16 mice.

**Figure 5—figure supplement 6.**
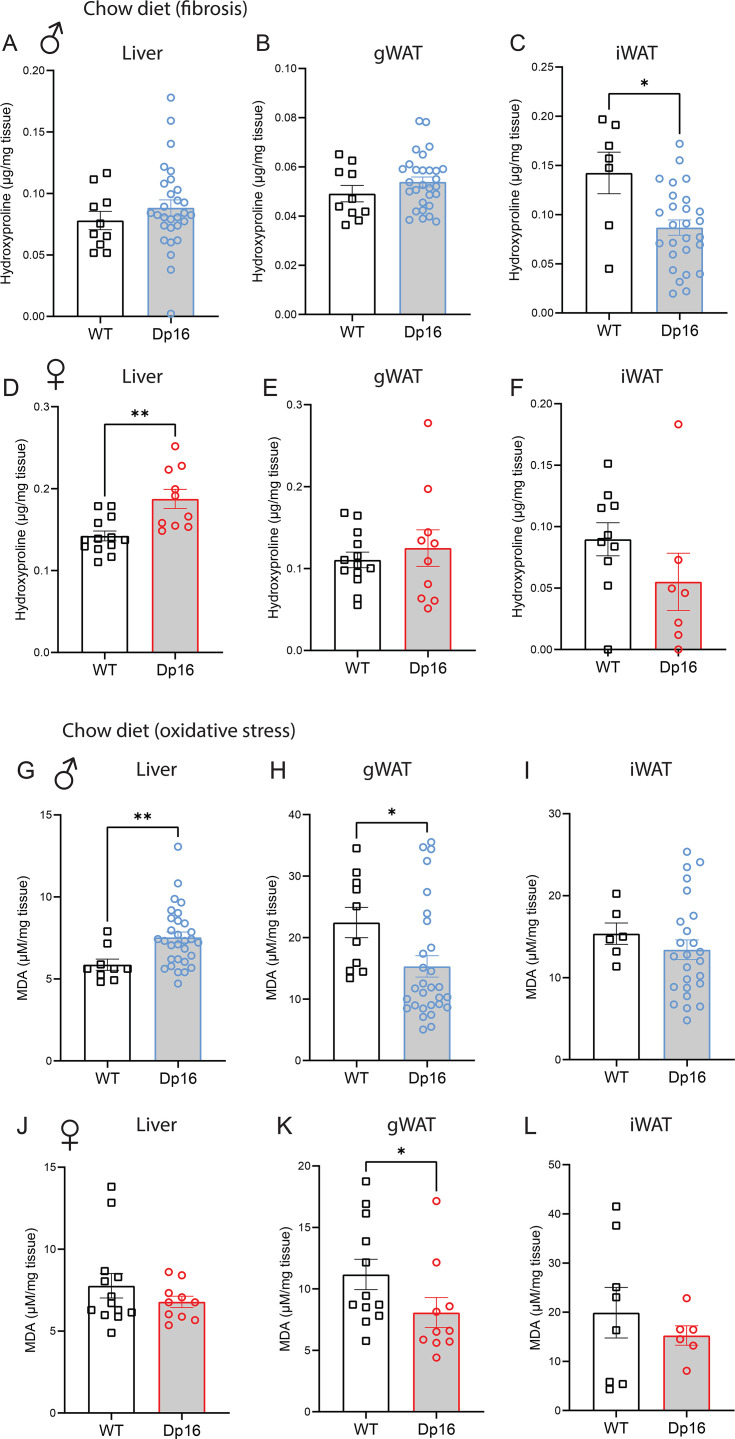
Hydroxyproline (marker of fibrosis) and malondialdehyde (marker of oxidative stress) levels in the liver, gWAT, and iWAT of chow-fed Dp16 mice. (**A–F**) Quantification of hydroxyproline content in the liver, gWAT, and iWAT of Dp16 male (**A–C**) and female (**D–F**) mice and their corresponding WT controls. gWAT, gonadal white adipose tissue; iWAT, inguinal white adipose tissue. Sample size: male WT = 7–10 and Dp16 = 27–29; female WT = 10–13 and Dp16 = 7–10. (**G–L**) Quantification of malondialdehyde (MDA) levels in the liver, gWAT, and iWAT of Dp16 male (**G–I**) and female (**J–L**) mice and their corresponding WT controls. gWAT, gonadal white adipose tissue; iWAT, inguinal white adipose tissue. Sample size: male WT = 6–10 and Dp16 = 25–30; female WT = 8–13 and Dp16 = 6–10. All data are presented as mean ± SEM. * *P*<0.05; ** *P*<0.01.

We performed gene ontology (GO) analysis to reveal which major biological pathways are altered in Dp16 mice that could contribute to their metabolic phenotypes. Among the top biological pathways upregulated in female mice are ER stress (gWAT, iWAT), immune activation (iWAT, BAT, liver, and muscle), fibrosis (iWAT and hypothalamus), and the top downregulated pathways are ATP synthesis and cellular respiration (BAT and skeletal muscle) and lipid metabolism (liver) (Figure 5C). In male mice, some of the top biological pathways upregulated include fibrosis (gWAT and iWAT), immune activation (BAT, liver, and hypothalamus), lipid metabolism (BAT), and receptor signaling (skeletal muscle), and the down-regulated pathways include glucose and lipid metabolism (iWAT, BAT, and liver) and cellular respiration (BAT and liver) (Figure 5C). We highlighted some of the DEGs involved in immune activation, ER stress, fibrosis, glucose and lipid metabolism, fat oxidation, mitochondrial respiration, and signaling in iWAT, BAT, liver, skeletal muscle, and hypothalamus (Figure 5—figure supplements 1–5). These transcriptomic changes parallel our metabolite data (Tables 1 and 2); both sets of data highlighted impaired lipid metabolism, a pro-inflammatory state, reduced mitochondrial health, and oxidative stress. Altogether, these data underscore major sex differences in tissue transcriptomes, but also revealed common and shared biological pathways affected in Dp16 males and females that underpin their shared metabolic phenotypes.

To confirm the biochemical correlates of our RNA-seq data, we examined markers of fibrosis (hydroxyproline) and oxidative stress (malondialdehyde) in liver, gWAT, and iWAT. Collagen content was significantly higher in Dp16 female liver and lower in male gWAT (Figure 5—figure supplement 6). Oxidative stress was significantly higher in Dp16 male liver and lower in gWAT; in females it was lower in gWAT (Figure 5—figure supplement 6). These results partly corroborate our transcriptomic and metabolomic data, and further indicate that fibrosis and oxidative stress occur in Dp16 mice in a sex- and tissue-dependent manner.

### Sexually dimorphic response of Dp16 mice to an obesogenic diet

**Figure 6.**
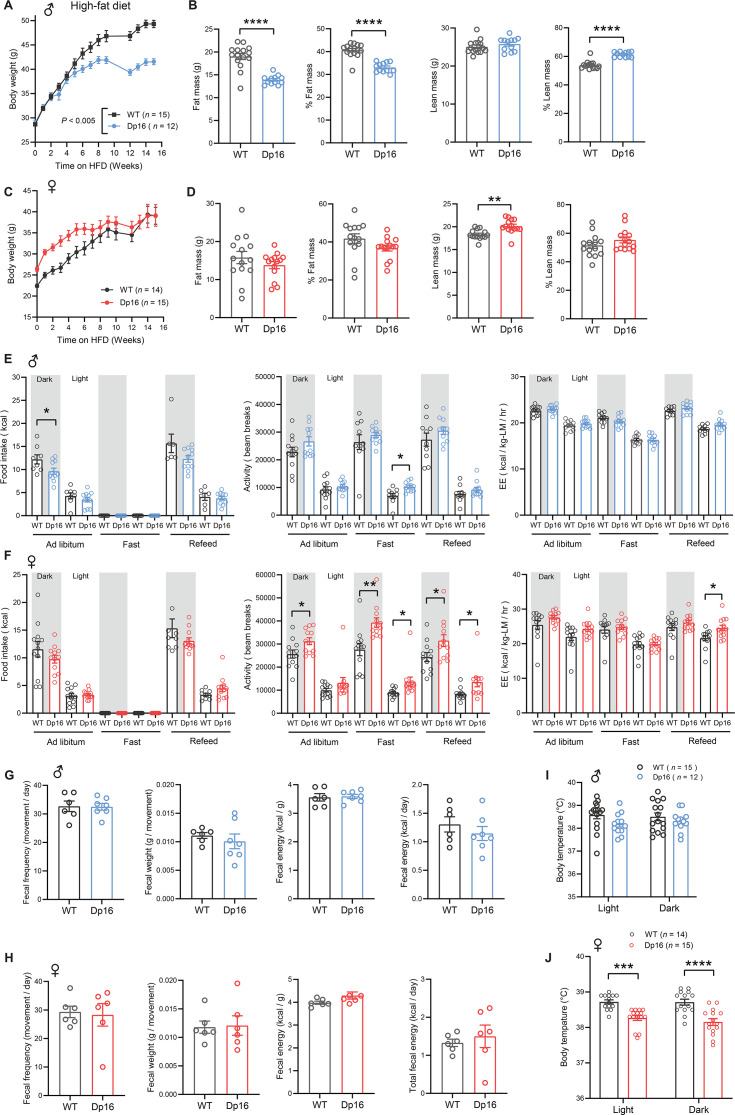
Sexually dimorphism in body weight, body temperature, food intake, and physical activity in Dp16 mice in response to a high-fat diet (HFD). (**A**) Body weight of HFD-fed male Dp16 and WT mice over time. (**B**) Absolute and relative (% of body weight) fat and lean mass in male mice (WT = 15; Dp16 = 12). (**C**) Body weight of HFD-fed female Dp16 and WT mice over time. (**D**) Absolute and relative (% of body weight) fat and lean mass in female mice (WT = 14; Dp16 = 14). (**E–F**) Food intake, total physical activity level, and energy expenditure of male (**E**) and female (**F**) Dp16 and WT mice across the circadian cycle (light and dark) and metabolic states (ad libitum fed, fast, refeed). Sample size for male (WT = 8; Dp16 = 11) and female (WT = 12; Dp16 = 12) mice. (**G–H**) Fecal frequency, average fecal weight, and fecal energy content (per gram and total) in male (**G**) and female (**H**) Dp16 and WT mice on HFD. Sample size for male (WT = 6; Dp16 = 7) and female (WT = 6; Dp16 = 6) mice. (**I–J**) Body temperature in the light and dark cycle of male (**I**) and female (**J**) Dp16 and WT mice on HFD. Sample size for male (WT = 15; Dp16 = 12) and female (WT = 14; Dp16 = 15) mice. All data are presented as mean ± SEM. * *P*<0.05; *** *P*<0.001; **** *P*<0.0001. For body weight over time, data were analyzed by 2-way ANOVA with Sidek post hoc tests.

Since the DS population is prone to developing obesity (Bertapelli et al., 2016; Aslam et al., 2022), we challenged the Dp16 mice with an obesogenic HFD and assessed how they handle metabolic stress associated with chronic high-fat feeding. There were striking sex differences in the response of Dp16 mice to an HFD. For the first five weeks on HFD, Dp16 males gained a similar amount of weight as the WT controls. From six weeks onward, their body weight diverged, with the WT males gaining significantly more weight compared to Dp16 males (Figure 6A), and this was reflected in much higher adiposity seen in the WT males (Figure 6B). Absolute lean mass was not different between genotypes in males, but % lean mass (when normalized to body weight) was higher in Dp16 males. In contrast to males, Dp16 females gained weight rapidly in the first 6 weeks on HFD, but by 7 weeks onward, the body weights of Dp16 females were no longer significantly different from WT controls (Figure 6C). Body composition analysis revealed no difference in fat mass, but modestly higher lean mass, in Dp16 females after 16 weeks on HFD (Figure 6D).

**Figure 6—figure supplement 1.**
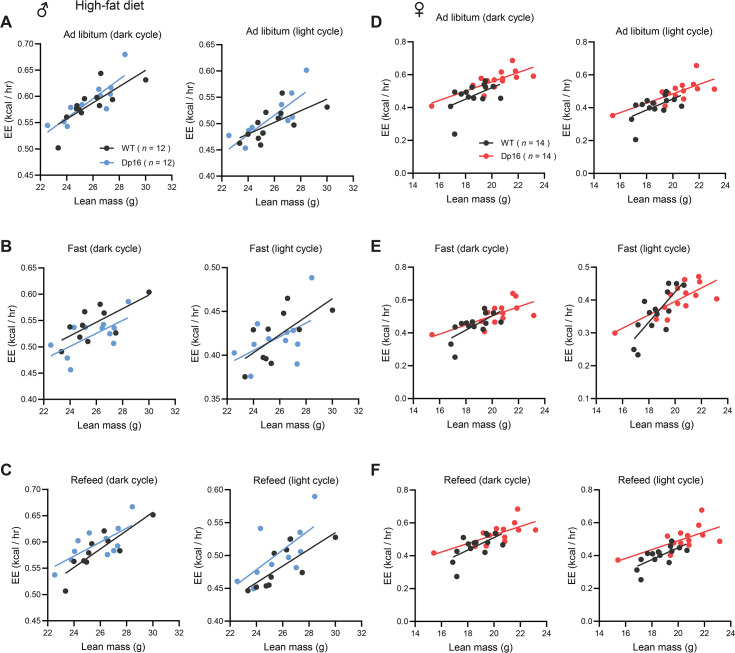
ANCOVA analysis of energy expenditure in HFD-fed mice where lean mass is used as a covariate. ANCOVA analysis of WT and Dp16 male mice across the circadian cycle (dark and light) in ad libitum fed (**A**), fasted (**B**), and refed (**C**) states. ANCOVA analysis of WT and Dp16 female mice across the circadian cycle (dark and light) in ad libitum fed (**D**), fasted (**E**), and refed (**F**) states. Male Sample size: WT = 12; Dp16 = 12. Female sample size: WT = 14; Dp16 = 14.

**Figure 6—figure supplement 2.**
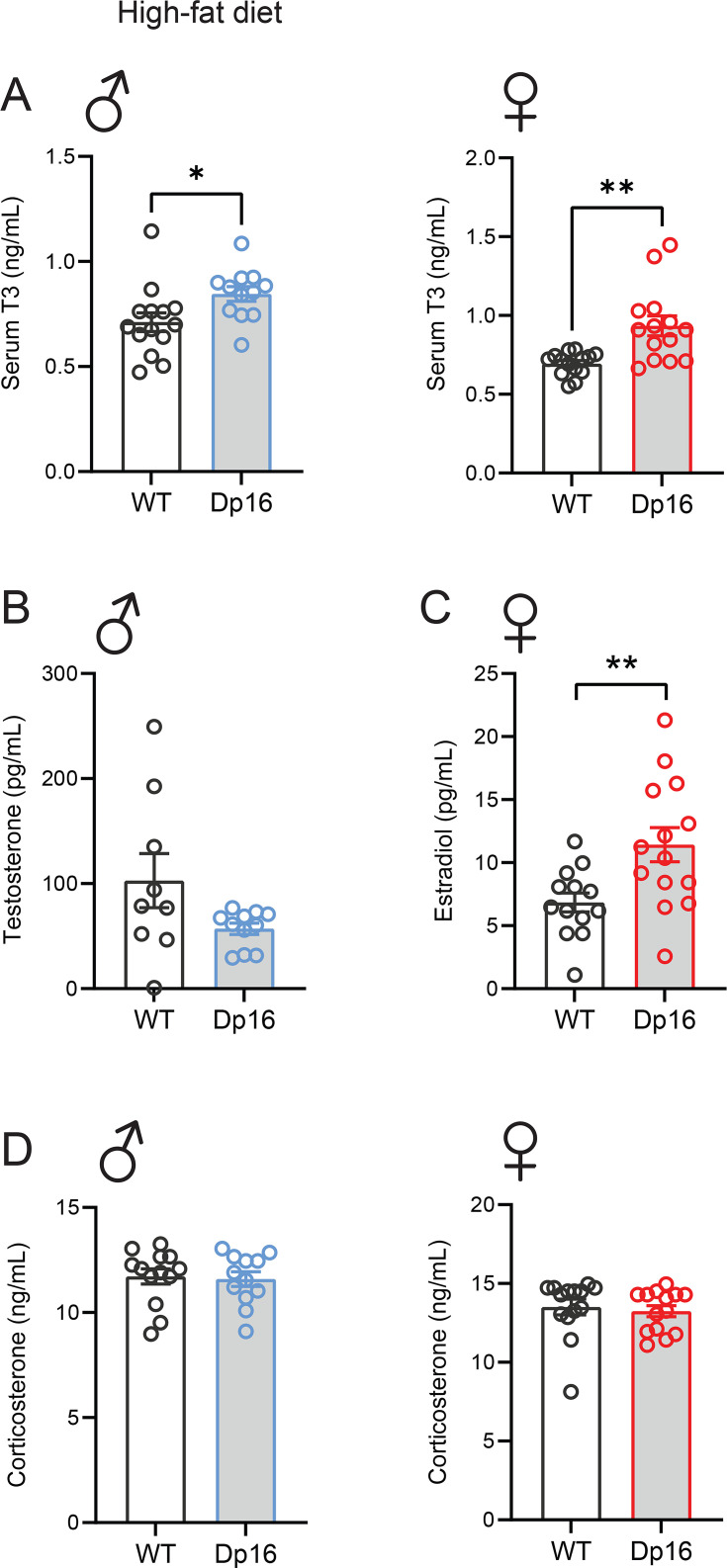
Serum Triiodothyronine (T3), sex and stress hormone levels in WT and Dp16 mice fed a high-fat diet. (**A**) Serum T3 levels in male and female mice. (**B**) Serum testosterone levels in male mice. (**C**) Serum estradiol levels in female mice. (**D**) Serum corticosterone in male and female mice. Sample size: male WT = 9–13; male Dp16 = 11–12; female WT = 14–15; female Dp16 = 14.

**Figure 6—figure supplement 3.**
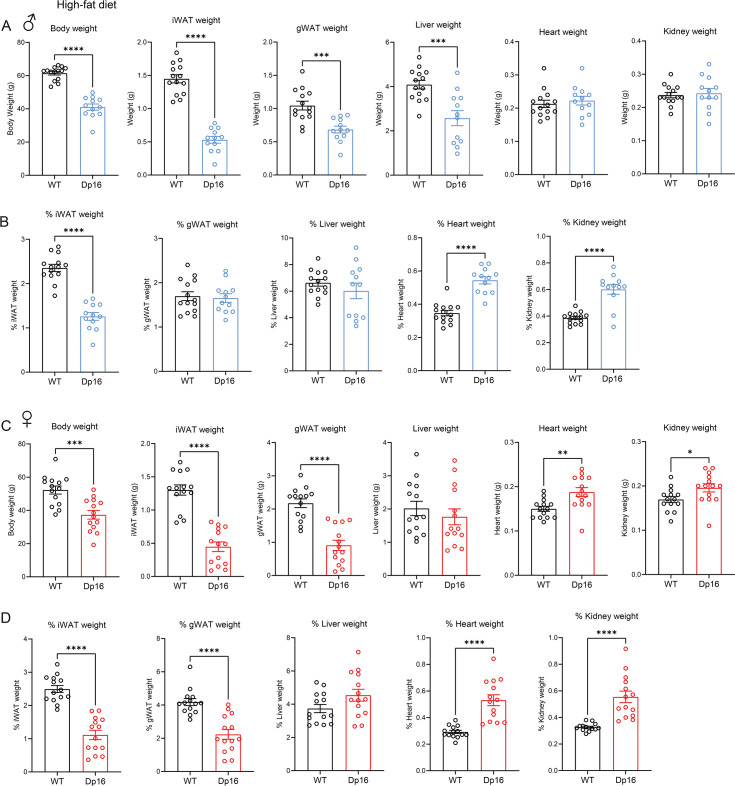
Body and tissue weights of high-fat diet (HFD)-fed male and female mice at termination of study. Tissues were collected from male mice (50 weeks old) after they had been fed a HFD for 34.5 weeks. Body weights and the absolute (**A**) and relative (**B**; % of body weight) weights of gWAT, iWAT, liver, and kidney in Dp16 and WT male mice. Female tissues (45 weeks old) were mice had been fed a HFD for 26 weeks. Body weights and the absolute (**A**) and relative (**B**; % of body weight) weights of gWAT, iWAT, liver, heart, and kidney in Dp16 and WT female mice. gWAT, gonadal white adipose tissue; iWAT, inguinal white adipose tissue. Sample size: WT male = 14; Dp16 male = 12; WT female = 14; Dp16 female = 12. All data are presented as mean ± SEM. * p<0.05; ** p<0.01; *** p<0.001; **** p<0.0001.

**Figure 6—figure supplement 4.**
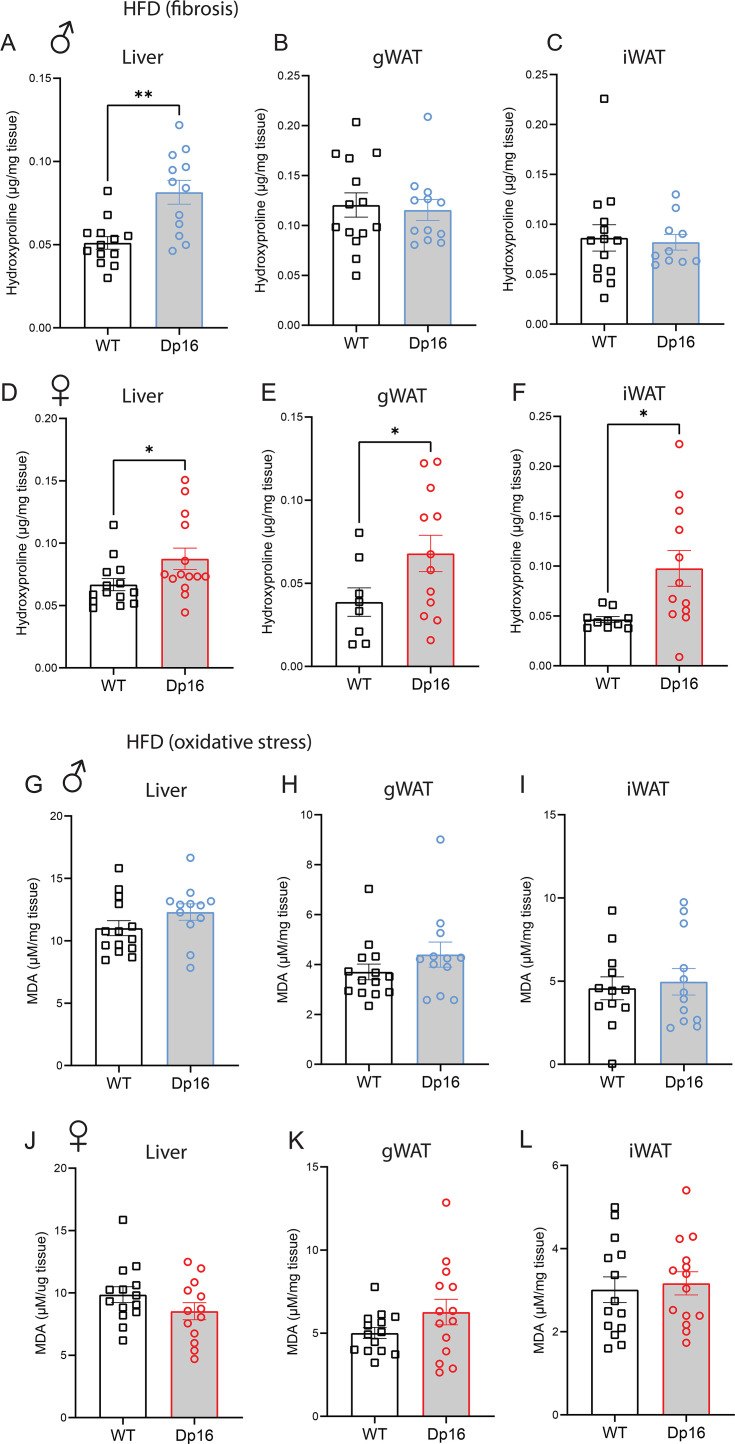
Hydroxyproline (marker of fibrosis) and malondialdehyde (marker of oxidative stress) levels in the liver, gWAT, and iWAT of Dp16 mice on high-fat diet (HFD). (**A–F**) Quantification of hydroxyproline content in the liver, gWAT, and iWAT of Dp16 male (**A–C**) and female (**D–F**) mice and their corresponding WT controls. gWAT, gonadal white adipose tissue; iWAT, inguinal white adipose tissue. Sample size: male WT = 13–14 and Dp16 = 10–12; female WT = 10–14 and Dp16 = 12–14. (**G–L**) Quantification of malondialdehyde (MDA) levels in the liver, gWAT, and iWAT of Dp16 male (**G–I**) and female (**J–L**) mice and their corresponding WT controls. gWAT, gonadal white adipose tissue; iWAT, inguinal white adipose tissue. Sample size: male WT = 13–14 and Dp16 = 12; female WT = 14 and Dp16 = 13–14. All data are presented as mean ± SEM. * *P*<0.05; ** *P*<0.01.

We performed indirect calorimetry analysis after the mice were on HFD for 12 weeks. We observed lower ad libitum food intake in Dp16 males in the dark/active cycle (Figure 6E), and this could contribute to lower weight gain over time. Dp16 males had modestly higher physical activity levels in the light cycle during fasting, but energy expenditure was not different from WT controls across the circadian cycles and metabolic states (fed, fasted, and refed) (Figure 6E). In contrast to the males, food intake was not different between genotypes in females (Figure 6F). However, Dp16 females had consistently higher physical activity levels in the dark cycle across different metabolic states (fed, fasted, refed). Although energy expenditure (normalized to lean mass) appeared to be slightly higher in Dp16 females, it was only significantly different in the light cycle during the refed period (Figure 6F). To rule out potential overestimation of energy expenditure, we performed ANCOVA analyses (using lean mass as a covariate of energy expenditure) (Tschöp et al., 2012). ANCOVA analyses indicated no differences in energy expenditure between genotypes of either sex across the circadian cycles and metabolic states (Figure 6—figure supplement 1).

Next, we determined whether there are any differences in nutrient absorption in the intestine by quantifying fecal output and frequency, as well as fecal energy content in mice on HFD. No differences in any of the fecal parameters were noted between genotypes of either sex (Figure 6G–H). Because we observed differences in the body temperature of chow-fed mice, we again measured the body temperature of mice on HFD. In contrast to higher body temperature of chow-fed males, Dp16 males on HFD appeared to have slightly lower body temperature in the light cycle, though not significant (Figure 6I). Like the chow-fed females, Dp16 females on HFD also had lower body temperature in both the light and dark cycles (Figure 6J). We measured serum triiodothyronine (T3) to determine whether altered thyroid hormone level contributes to lower body temperature. Contrary to expectation, both Dp16 males and females had elevated serum T3 levels (Figure 6—figure supplement 2). We also measured serum levels of sex and stress hormones, as these could contribute to our phenotypes. Corticosterone levels were not different between genotypes of either sex. Testosterone levels in males appeared lower but not significant; estradiol levels, however, were higher in Dp16 females (Figure 6—figure supplement 2), possibly contributing to their lower body temperature and higher physical activity (Krause et al., 2021; Correa et al., 2015; Zhang et al., 2020; Krajewski-Hall et al., 2018).

At termination of study, we assessed whether there are differences in tissue weight in Dp16 mice on HFD. Tissues were collected from male mice at 50 weeks of age when they were on HFD for 34.5 weeks. At the time of termination, body weights and tissue weights of gWAT, iWAT, and liver were significantly lower in Dp16 males relative to WT controls (Figure 6—figure supplement 3). Although the absolute weights of heart and kidney were not different between genotypes, the relative weights (% of body weight) of heart and kidney were significantly higher in Dp16 males. For females, tissues were collected at 45 weeks of age (on HFD for 26 weeks). Although the body weights of Dp16 females on HFD were not different from WT controls at 35 weeks of age (on HFD for 16 weeks), there were significantly lower at 45 weeks of age (on HFD for 26 weeks) (Figure 6—figure supplement 3). The absolute and relative weights of gWAT and iWAT were significantly lower in Dp16 females. The absolute and relative weights of heart and kidney, however, were higher in Dp16 females.

We also noted that a marker of fibrosis (hydroxyproline content) was significantly higher in Dp16 male liver, as well as in Dp16 female liver, gWAT, and iWAT. A marker of oxidative stress (malondialdehyde content) was not significantly different between genotypes of either sex in liver, gWAT, and iWAT (Figure 6—figure supplement 4). Taken together, these data indicate major sex differences in the physiological response of Dp16 mice to an obesogenic diet.

### High-fat diet exacerbates glucose intolerance and insulin resistance in Dp16 mice

**Figure 7.**
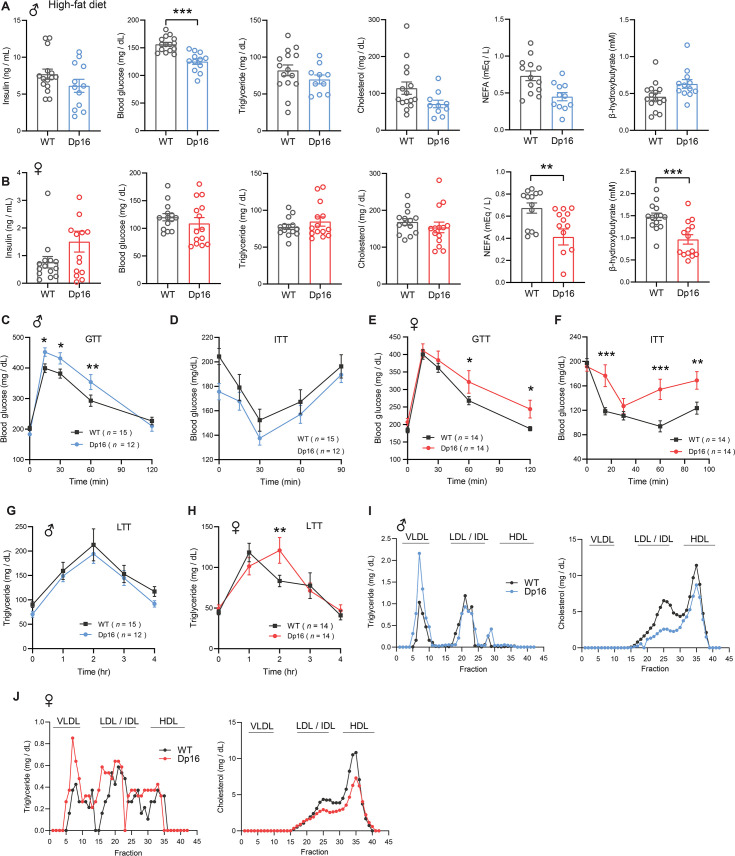
Exacerbated glucose intolerance and insulin resistance in Dp16 mice fed a high-fat diet (HFD). (**A–B**) Overnight fasting insulin, blood glucose, serum triglyceride, cholesterol, non-esterified free fatty acids (NEFA), and β-hydroxybutyrate (ketone) in male (**A**) and female (**B**) Dp16 and WT mice on HFD. Sample size for male mice (WT = 15; Dp16 = 12) and female mice (WT = 14; Dp16 = 14). (**C–F**) Exacerbated glucose intolerance as determined by the glucose tolerance test (GTT) in male (**C**) and female (**E**) Dp16 compared to WT controls on HFD. Exacerbated insulin resistance as determined by the insulin tolerance test (ITT) in male (**D**) and female (**F**) Dp16 compared to WT controls. Sample size for male mice (WT = 15; Dp16 = 12) and female mice (WT = 14; Dp16 = 14). (**G–H**) The rate of triglyceride clearance in response to lipid gavage as determined by the lipid tolerance test (LTT) in male (**G**) and female (**H**) Dp16 and WT mice. Sample size for male mice (WT = 15; Dp16 = 12) and female mice (WT = 14; Dp16 = 14). (**I–J**) Pooled mouse sera from male (**I**) and female (**J**) Dp16 and WT mice were fractionated by fast protein liquid chromatography (FPLC), and the triglyceride and cholesterol content of each fraction was quantified. Fractions corresponding to very-low density lipoprotein (VLDL), low-density lipoprotein (LDL), intermediate-density lipoprotein (IDL), and high-density lipoprotein (HDL) are indicated. All data are presented as mean ± SEM. * p<0.05; ** p<0.01; *** p<0.001; **** p<0.0001. For all tolerance tests, data were analyzed by two-way ANOVA with Sidek post hoc tests.

Since Dp16 mice on a standard chow diet developed overt insulin resistance and dyslipidemia, we determined whether HFD would further exacerbate these phenotypes. In overnight (16 h) fasted males, serum insulin, TG, cholesterol, NEFA, and β-hydroxybutyrate (ketone) levels were not different between genotypes (Figure 7A), even though Dp16 males had significantly lower body weight and adiposity. Fasting blood glucose levels, however, were lower in Dp16 males, likely reflecting lower adiposity. The Dp16 females appeared to have higher fasting insulin levels, though not significant (Figure 7B). Fasting blood glucose and serum TG and cholesterol levels were not different between genotypes in female mice. Serum NEFA and β-hydroxybutyrate (ketone) levels were significantly lower in Dp16 females (Figure 7B), suggesting lower fasting-induced lipolysis and hepatic fat oxidation.

Next, we subjected Dp16 mice on HFD to glucose tolerance test. Both Dp16 males and females showed exacerbated glucose intolerance compared to WT controls (Figure 7C and E). Direct assessment of insulin sensitivity showed that Dp16 males and females have significantly reduced glucose clearance in response to insulin injection compared to WT controls, with Dp16 females exhibiting a more pronounced insulin resistance phenotype (Figure 7D and F).

Given that Dp16 mice on a standard chow diet had marked deficit in lipid clearance, we again performed lipid tolerance tests to assess whether Dp16 mice on HFD have worsening lipid handling capacity. No differences were observed between genotypes in male mice, whereas Dp16 females had a modest impairment in lipid clearance compared to WT controls (Figure 7G–H). Analysis of lipoprotein profiles showed that both Dp16 males and females have higher VLDL-TG and lower LDL-C compared to WT controls, with the effect more pronounced in males (Figure 7I–J). Taken together, these data indicate that despite divergent weight gain in response to HFD, both Dp16 males and females show exacerbated insulin resistance and dyslipidemia.

## Discussion

In this study, we show that triplication of Hsa21 gene orthologs in the Dp16 mouse model causes profound disruption in systemic metabolism. By combining deep phenotyping with multi-omics approaches, we show that gene dosage imbalance contributes to major changes in transcriptomes, metabolomes, and biological pathways that link to systemic insulin resistance, glucose intolerance, impaired lipid clearance, dyslipidemia, and exacerbate metabolic stress induced by an obesogenic diet. Our findings provide valuable insights and plausible mechanistic explanations for the prevalence of obesity, dyslipidemia, and diabetes in the DS population.

We show that most triplicated Hsa21 gene orthologs are expressed at the expected ~1.5-fold or higher across six metabolic tissues, but in a striking sex- and tissue-specific manner. Variegated overexpression of Hsa21 genes has been previously noted in individuals with DS (Donovan et al., 2024). In Dp16 mice, female adipose depots exhibit the highest number of differentially expressed triplicated genes, and this is associated with the substantial weight gain observed in Dp16 females. Males, in contrast, have relatively stable body weight and adiposity under standard chow diet despite having similar gene dosage imbalance. These sex differences underscore the complex interactions between triplicated genes and sex-dependent regulatory mechanisms that influences tissue transcriptomes (Blencowe et al., 2022), fat mass expansion and systemic energy metabolism (Kelly and Jones, 2013; Hevener and Correa, 2025). Our data reinforces sex as an important biological determinant of metabolic risk in DS, consistent with documented sex differences in the susceptibility of individuals with DS to developing obesity and metabolic impairments (Aslam et al., 2022; González-Agüero et al., 2011; Hsieh et al., 2014; Pierce et al., 2019; Bhaumik et al., 2008; Rajkovic Vuletic et al., 2025).

Although their weight trajectories diverge, both male and female Dp16 mice exhibit hallmark features of metabolic dysfunction, including fasting hyperinsulinemia, glucose intolerance, insulin resistance, and impaired lipid clearance. These core phenotypes shared between the sexes arise in spite of divergent adiposity, indicating that dysregulated systemic metabolism is a primary consequence of triplicated gene dosage imbalance rather than a secondary effect of increased fat mass. The insulin resistance phenotype, as well as impaired TG clearance and alterations in lipoprotein profiles are consistent with the propensity of individuals with DS to developing type 2 diabetes, dyslipidemia and altered fasting lipid profiles (Van Goor et al., 1997; Bertapelli et al., 2016; Fonseca et al., 2005; Aslam et al., 2022; Valentini et al., 2017; Buonuomo et al., 2016; Adelekan et al., 2012; Gross et al., 2019; Garcia-de la Puente et al., 2021; de la Piedra et al., 2017). These parallels strengthen the translational relevance of the Dp16 model. Future studies are needed to determine which aspects of lipid metabolism—hepatic lipid export, adipose tissue lipolysis, lipoprotein turnover—are dysregulated in DS. Nevertheless, our assessments of mitochondrial function and biochemical markers of fibrosis and oxidative stress, as well as our transcriptomic, metabolomic, and pathway enrichments analyses have provided some mechanistic insights. Our data point to a combination of changes—impaired glucose and lipid metabolism, mitochondrial function, ER and oxidative stress, fibrosis, and low-grade inflammation—all of which are known drivers of adverse metabolic outcomes (Hotamisligil, 2010; Sun et al., 2013; Bhatti et al., 2017). These collective changes act in concert across major metabolic tissues to disrupt local and systemic energy metabolism in Dp16 mice.

Our metabolomic analyses highlighted extensive remodeling of the metabolome of liver and serum. Strikingly, the majority of differential metabolites are not shared between the sexes. This once again underscores the unexpected and complex interactions of triplicated genes and biological sex in determining phenotypic outcomes. The differential metabolites are clustered around pathways related to lipid, amino acid, and bile acid metabolism; some of these pathways have been previously documented in DS (Dunn et al., 2026; Powers et al., 2019). Despite sex differences, we noted that several classes of metabolites—bile acids, acylcarnitine, eicosanoids, phospholipids, fatty acid conjugates—are shared by Dp16 males and females and that the directionality of change is also broadly consistent between the sexes. Altered bile acid pools, including both primary and conjugated bile acids made in the liver (e.g. taurochenodeoxycholic acid, chenodeoxycholymethionine, hyocholic acid, glycohyocholic acid) and secondary bile acids made by gut bacteria (e.g. lithocholic acid, taurolithocholate sulfate, glycoursodeoxycholid acid, keto and diketo lithocholic acid), suggest potential changes in gut–liver axis and bile-acid–regulated metabolic signaling (Perino et al., 2021; Fleishman and Kumar, 2024; Wang et al., 2024) that can contribute to metabolic dysfunction. The directionality of change in multiple immuno-regulatory eicosanoids (Dennis and Norris, 2015) and fatty acids (Yore et al., 2014; Kuda et al., 2016; Serhan and Levy, 2018) appears to promote a pro-inflammatory state in Dp16 mice, a phenotype that is also supported by our multi-tissue transcriptomic data. Interestingly, several hepatic and serum phospholipid species (e.g. Lyo-PC, Lyso-PA, Lyso-PA) are either elevated or reduced in Dp16 mice. Given the complex signaling roles for some of these phospholipids (O’Donnell et al., 2018), we speculate that these changes may underline some aspects of the metabolic deficits in Dp16 mice. Thus, remodeling of tissue and serum metabolomes, in combination with major changes in transcriptomes across tissues, likely contributed to the pronounced metabolic dysfunction in Dp16 mice.

The introduction of an obesogenic diet helped us to reveal the complex interplay of gene and environment in the context of Hsa21 gene dosage imbalance. This becomes relevant as individuals with DS live increasingly longer lives, and can have varied lifestyles and diet. Despite opposite weight trajectories on HFD–males gaining less and females initially gaining more–both Dp16 male and female mice develop worsened glucose intolerance and insulin resistance. This dissociation between weight gain and metabolic impairment again point to gene dosage imbalance as the primary cause of metabolic dysfunction rather than a secondary effect of altered adiposity. The increased fibrosis observed in adipose tissue and liver in Dp16 mice on HFD suggests that chronic nutritional stress exacerbates extracellular matrix remodeling, a change in tissue architecture that is known to compromise adipose tissue and liver function (Sun et al., 2013; Koyama and Brenner, 2017).

Although not the focus of the present study, we unexpectedly discovered that an obesogenic diet causes heart enlargement in Dp16 mice; this phenotype was not observed in Dp16 mice fed a standard chow. The cause of heart enlargement in response to a HFD is presently unknown. Given that congenital heart defect is frequently seen in DS (Dimopoulos et al., 2023), prior studies in DS mouse models have been focused on the contribution of triplicated genes to developmental heart defects (e.g. atrial or ventricular septal defect; Lana-Elola et al., 2016; Li et al., 2016). Our data suggests that the complex interactions of genetics and diet may predispose adult individuals with DS to cardiovascular complications independent of congenital heart abnormalities.

Among the 115 Hsa21 triplicated gene orthologs located on the syntenic region of mouse chromosome 16, several are known to affect metabolism, oxidative stress, inflammatory response, and/or fibrosis. These include *Dyrk1a* (Bertrand et al., 2025), *Dscr1*/*Rcan1* (Peiris et al., 2016; Peiris et al., 2012), *AtpJ* (Ren et al., 2024), *Atp5o* (Rönn et al., 2009), *Nrip1* (Tsagkaraki et al., 2023), *Tiam1* (Kowluru et al., 2014; Syed et al., 2010), *Prdm15* (Mzoughi et al., 2020), *Ripk4* (Zhang et al., 2024), *Znf295* (Mirhafez et al., 2016), *Hmgn1* (Nanduri et al., 2022), *Cbr1* (Bell et al., 2021), *Bach1* (Jin et al., 2023), *Fam3b* (Wei et al., 2025), *Ets2* (Birsoy et al., 2011), *Adamts5* (Bauters et al., 2016; Bauters et al., 2018), *Usp16* (Gan et al., 2023), *Runx1* (Kilbey et al., 2017), *Sim2* (Wall et al., 2023), and the interferon receptor gene locus (*Ifnar2*, *Il10rb*, *Ifnar1*, *Ifngr2*) (Sullivan et al., 2016; Waugh et al., 2023). Interestingly, some of these triplicated genes show sexually dimorphic expression in gWAT (*Il10rb*, *Cbr1*, *Ets2*, *Nrip1*), iWAT (*Il10rb*, *Runx1*, *Adamts5*, *Tiam1*, *Rcan1*, *Ripk4*, *Bach1*, *Nrip1*, *Dyrk1a*, *Fam3b*), BAT (*Cbr1*, *Usp16*, *Ets2*, *Atp5o*, *Nrip1*), Liver (*Tiam1*, *Rcan1*, *Bach1*, *Prdm15*), skeletal muscle (*Adamts5*, *Ifnar2*, *Rcan1*, *Ets2*, *Atp5o*, *Nrip1*), and hypothalamus (*Ripk4*, *Sim2*); these sex-biased expression patterns may contribute to the sex differences in Dp16 metabolic phenotypes. In striking contrast to other DS phenotypes, normalizing the expression dosage of the interferon receptor gene locus does not reverse the hepatic lipid metabolism profile (Dunn et al., 2026). None of the other triplicated candidate genes were studied in the context of trisomy; thus, it is unknown whether normalizing these triplicated genes–individually or in combination–could reverse some or all of the metabolic phenotypes described. Systematic genetic dissection of dosage-sensitive genes in the context of trisomy—an approach successfully used for other DS phenotypes (Lana-Elola et al., 2016; Jiang et al., 2015; Sloan et al., 2023; Ahumada Saavedra et al., 2025)–are required to establish their necessity and sufficiency in promoting metabolic dysfunction. Given the complex metabolic phenotypes of Dp16, we anticipate that multiple dosage-sensitive genes are likely to work additively or synergistically to disrupt systemic glucose and lipid metabolism.

Several limitations of the study, however, are noted. First, while Dp16 mouse model contains ~58% of the Hsa21 gene orthologs (Li et al., 2007; Lana-Elola et al., 2016), it does not contain the full complement of Hsa21 gene orthologs. Although no single mouse model fully recapitulates all DS phenotypes (Herault et al., 2017), in light of the impact and complex combinatorial effects of the triplicated genes on phenotypic outcomes and tissue transcriptomes (Duchon et al., 2021; Pereira et al., 2009), it is worthwhile in future studies to comprehensively examine the metabolic phenotypes of the triple compound model (Dp16;Dp10;Dp17) carrying the full complement of the Hsa21 gene orthologs (Yu et al., 2010). The large-scale breeding, cost, and labor associated with generating the compound mice have been a major challenge; however, progress has recently been made to overcome this hurdle (Li et al., 2021). Secondly, Dp16 is a segmental duplication model and not a trisomic model with an independently segregating chromosome. Recent studies have suggested that the presence of an extra chromosome (i.e. trisomy) can affect phenotypes and disomic gene expressions beyond the gene dosage effect of triplicated genes (Xing et al., 2023). Disentangling dosage-dependent versus chromosome-dependent effects will be an important future direction. Thirdly, although we observed major perturbations across tissue transcriptomes, not all changes in mRNAs would translate into corresponding changes in protein levels, and vice versa (Liu et al., 2016; Liu et al., 2017). Future studies incorporating proteomics data would enhance and complement our transcriptomic results. Fourthly, while our studies were ongoing, Tolu and co-workers also reported the glucose intolerance and insulin resistance phenotype in Dp16 mice (Tolu et al., 2025). The authors also showed reduced insulin content in the pancreatic β-cell without changes in β-cell mass. Notably, in our study, we did not measure pancreatic insulin content. We also did not include transcriptomic data from the pancreas, as none of the RNA samples passed the quality control needed for RNA sequencing.

In summary, our data show that the triplication of Hsa21 gene orthologs severely disrupts metabolic homeostasis through concerted perturbations in transcriptome, metabolome, tissue remodeling, mitochondrial function, and glucose and lipid metabolism. Our findings provide physiological contexts for ongoing studies aiming to understand the vulnerability of DS population to developing metabolic disorders. The striking and extensive sex differences uncovered here argue that metabolic studies and therapeutic strategies in DS should account for sex as an important biological variable. Our study highlighted both shared and sex-specific mechanisms of metabolic dysfunction in a DS mouse model, and the impact of gene dosage imbalance on altering whole-body metabolism. The wealth of molecular, biochemical, and physiological data help lay the crucial groundwork for genetic dissection of dosage-sensitive genes causally linked to metabolic dysfunction, and to inform efforts at identifying actionable therapeutic targets that can mitigate one or more aspects of metabolic deficits seen in individuals with DS.

## Materials and methods

### Mouse model

Dp(16)1Yey/+ (abbreviated Dp16) mice and wild-type (WT) littermate controls, on C57BL/6 J genetic background, were obtained from the Jackson Laboratory (Strain # 013530). Mice were fed a standard chow (Envigo; 2018 SX) or a HFD (60% kcal derived from fat, #D12492, Research Diets, New Brunswick, NJ). Mice were housed in polyethylene terephthalate (PET) cages on a 12 hr:12 hr light-dark photocycle (lights on at 6 am, lights off at 6 pm) with ad libitum access to water and food. For the HFD-fed group, HFD was provided beginning for 26–34 weeks. At termination of the study, all mice were fasted for 2 hr and euthanized. The age of mice at the time of tissue harvest: male and female mice fed a standard chow were 27.5 weeks old; male mice fed an HFD were 50 weeks old (on HFD for 34.5 weeks); female mice fed an HFD were 45 weeks old (on HFD for 26 weeks). Tissues were collected, snap-frozen in liquid nitrogen, and kept at -80 °C until analysis.

All mouse protocols were approved by the Institutional Animal Care and Use Committee of the Johns Hopkins University School of Medicine (animal protocol # MO22M367). All animal experiments were conducted in accordance with the National Institute of Health guidelines and followed the standards established by the Animal Welfare Acts.

### Body composition analysis

Body composition analyses for total fat mass, lean mass, and water content were determined using a quantitative magnetic resonance instrument (Echo-MRI-100, Echo Medical Systems, Waco, TX) at the Mouse Phenotyping Core facility at Johns Hopkins University School of Medicine.

### Indirect calorimetry

Chow- or HFD-fed Dp16 male and female mice and WT littermates were used for simultaneous assessments of daily body weight change, food intake (corrected for spillage), physical activity, and whole-body metabolic profile in an open flow indirect calorimeter (Comprehensive Laboratory Animal Monitoring System, CLAMS; Columbus Instruments, Columbus, OH) as previously described (Sarver et al., 2020). In brief, data were collected for three days to confirm mice were acclimatized to the calorimetry chambers (indicated by stable body weights, food intakes, and diurnal metabolic patterns), then data were analyzed for the subsequent three days. Mice were observed with ad libitum access to food, throughout the fasting process, and in response to refeeding. Rates of oxygen consumption ($\overset{˙}{V}$_O2_; mL·kg^–1^·hr^–1^) and carbon dioxide production ($\overset{˙}{V}$_CO2_; mL·kg^-1·^hr^–1^) in each chamber were measured every 24 min. Respiratory exchange ratio (RER = $\overset{˙}{V}$_CO2_/$\overset{˙}{V}$_O2_) was calculated by CLAMS software (version 5.18) to estimate relative oxidation of carbohydrates (RER = 1.0) versus fats (RER = 0.7), not accounting for protein oxidation. Energy expenditure (EE) was calculated as EE = $\overset{˙}{V}$_O2_× [3.815 + (1.232×RER)] and normalized to lean mass. We also performed ANCOVA analysis on EE using body weight as a covariate (Tschöp et al., 2012). Physical activities (total and ambulatory) were measured by infrared beam breaks in the metabolic chamber. Average metabolic values and summed intake and activity values were calculated per subject and averaged across subjects for statistical analysis by Student’s t-test.

### Body temperature

Deep colonic temperature was measured by inserting a lubricated (Medline, water soluble lubricating jelly, MDS032280) probe (Physitemp, BAT-12 Microprobe Thermometer) into the anus of mice at a depth of 2 cm. Stable numbers were recorded in both the dark and light cycle for each mouse.

### Fecal bomb calorimetry and assessment of fecal parameters

Fecal pellet frequency and average fecal pellet weight were monitored by housing each mouse singly in clean cages and counting the number of fecal pellets and recording their weight at the end of a 24 hr period. Fecal pellets were shipped to the University of Michigan Animal Phenotyping Core for fecal bomb calorimetry. Briefly, fecal samples were dried overnight at 50 °C prior to weighing and grinding them to powder. Each sample was mixed with wheat flour (90% wheat flour, 10% sample) and formed into 1.0 g pellet, which was then secured into the firing platform and surrounded by 100% oxygen. The bomb was lowered into a water reservoir and ignited to release heat into the surrounding water. Together these data were used to calculate fecal pellet frequency (bowel movements/day), average fecal pellet weight (g/bowel movement), fecal energy (cal/g feces), and total fecal energy (kcal/day).

### Glucose, insulin, and lipid tolerance tests

All tolerance tests were conducted as previously described (Rodriguez et al., 2016; Lei and Wong, 2019; Tan et al., 2016). For glucose tolerance tests (GTTs), mice were fasted for 6 hr before glucose injection. Glucose (Sigma, St. Louis, MO) was reconstituted in saline (0.9 g NaCl/L) to a final concentration of 1 g/10 mL (for the chow-fed mice) or 2 g/10 mL (for the HFD-fed mice), sterile-filtered, and injected intraperitoneally (i.p.) at 1 mg/g body weight (i.e. 10 μL/g body weight for chow-fed mice or 5 μL/g body weight of HFD-fed mice). Blood glucose was measured at 0, 15, 30, 60, and 120 min after glucose injection using a glucometer (NovaMax Plus, Billerica, MA). For ITTs, food was removed 2 hr before insulin injection. 6.5 μL of insulin stock (4 mg/mL; Gibco) was diluted in 10 mL of saline, sterile-filtered, and injected i.p. at 0.75 U/kg body weight (i.e. 10 μL/g body weight). Blood glucose was measured at 0, 15, 30, 60, and 90 min after insulin injection using a glucometer (NovaMax Plus). For lipid tolerance tests (LTTs), mice were fasted for 12 hr and then injected i.p. with 20% emulsified Intralipid (soybean oil; Sigma; 10 μL/g of body weight). Sera were collected via tail bleed using a Microvette CB 300 (Sarstedt) at 0, 1, 2, 3, and 4 hr post-injection. Serum TG levels were quantified using kits from Infinity Triglycerides (Thermo Fisher Scientific).

### Fasting glucose, insulin, and lipid profile

Mice were fasted overnight (~16 hr), beginning at 1 hr before the dark cycle (around 5 pm). Clean cages were provided before food withdrawal. Overnight fasting blood glucose levels from tail bleed were measured using a glucometer. Serum was collected at the 16 hr fast (around 10 am in the morning) for insulin ELISA, as well as for the quantification of TG, cholesterol, NEFAs, and β-hydroxybutyrate concentrations.

### Blood and tissue chemistry analysis

Tail vein blood samples were allowed to clot on ice and then centrifuged for 10 min at 10,000 × *g*. Serum samples were stored at –80 °C until analyzed. Serum TGs and cholesterol were measured according to manufacturer’s instructions using an Infinity kit (Thermo Fisher Scientific, Middletown, VA). NEFAs were measured using a Wako kit (Wako Chemicals, Richmond, VA). Serum β-hydroxybutyrate (ketone) concentrations were measured with a StanBio Liquicolor kit (StanBio Laboratory, Boerne, TX). Serum insulin (Crystal Chem, 90080), T3 (Calbiotech, T3043T-100), testosterone (Cayman, 582701), estradiol (Cayman, 501890), corticosterone (Cayman, 501320), and alanine aminotransferase (ALT; abcam, ab282882) levels were measured using commercial kits according to manufacturer’s instructions.

Hydroxyproline assay (Sigma Aldrich, MAK569) was used to quantify total collagen content in liver, hypothalamus, and adipose tissues according to the manufacturer’s instructions, with specific optimizations for each tissue type. Tissues were homogenized in deionized water using a bead mill homogenizer. Liver, iWAT, and gWAT were homogenized to a concentration of 0.1 mg tissue/µL. Due to the small and variable tissue mass, hypothalami were homogenized in a fixed volume of 110–120 µL to yield sufficient volume for processing. All samples were hydrolyzed with an equal volume of HCl at 120 °C for 3 hr. The volume of hydrolyzed tissue samples plated for the dehydration step was optimized for each tissue type and experimental group to ensure all measurements fell within the linear range of the standard curve. Hydroxyproline content was calculated based on a standard curve and normalized to tissue weight.

Lipid peroxidation levels (marker of oxidative stress) in liver, hypothalamus, and adipose tissues were assessed by the quantification of malondialdehyde (MDA) levels via the Thiobarbituric Acid Reactive Substances (TBARS) assay (Cayman Chemical, 700870) according to the manufacturer’s instructions.

### Serum lipoprotein-triglyceride and cholesterol analysis by FPLC

Food was removed for ~2 hr (in the light cycle) prior to blood collection. Sera collected from mice were pooled (n=10–14/group) and sent to the Mouse Metabolism Core at Baylor College of Medicine for fast protein liquid chromatography (FPLC) separation. A total of 45 fractions were collected, and TG and cholesterol in each fraction were quantified.

### Extraction of hepatic lipids

Lipid extraction from frozen liver samples was performed using a modified Folch method (Folch et al., 1957). Briefly, approximately 25 mg of frozen liver tissue was weighed and homogenized in 400 µL of cold sucrose buffer (250 mM sucrose, 10 mM Tris, 1 mM EDTA) using a bead beater (FastPrep-24, MP Biomedical). The samples underwent three rounds of 20 s bead beating cycles. Lipids were then extracted from the liver homogenate by adding 1.5 mL of a chloroform:methanol (2:1, v/v) solution to the mixture. The sample was vortexed thoroughly and centrifuged at 1700 rpm for 5 min at 4℃ to separate the phases. The lower chloroform phase, containing lipids, was carefully transferred to a new tube and split equally into two separate tubes. Each tube was then dried in a speed vacuum to dehydrate samples. The dried lipid extracts were reconstituted in 50 µL of chloroform for lipid analysis and quantification by thin-layer chromatography (TLC) or in 50 µL of tert-butanol:methanol:TritonX-100 solution (3:1:1, v/v/v) for cholesterol quantification. Reconstituted samples were either used immediately or stored at –80 °C until analysis.

### Separation of lipid classes and quantification by thin-layer chromatography

TLC was performed as described by Ring et al., 2002 to separate specific classes of lipids. To separate and quantify lipid classes from liver tissue, silica gel (Analtech, Preadsorbent Silica Gel G UNIPLATES Channeled, 20×20 cm, 250 µm, Cat #P31911) plates were pre-washed in methanol, air-dried, and then subsequently equilibrated by pre-washing in hexane:diethyl ether:acetic acid (H:D:A; 80:20:1, v/v/v) solvent system. The plates were allowed to air-dry completely before lipid spotting. Lipid extracts (2 µL) and lipid standards (2 µL, 5 mg/mL in chloroform, Millipore Sigma, Cat# 1787-1AMP, Supelco) were spotted onto the plates, and lipids were separated in H:D:A (80:20:1, v/v/v) to resolve triacylglycerols (TAG) and DAGs. After development, the plates were air-dried and exposed to iodine vapor in a pre-equilibrated tank overnight to visualize lipid spots. For quantification, the plates were imaged, and lipid spots were analyzed using ImageJ software (https://imagej.net/) to determine the intensity of each lipid spot relative to the known lipid standards and normalized to tissue weight.

### Hepatic cholesterol quantification

Following tissue extraction, total cholesterol content was quantified using the Infinity Cholesterol Reagent kit (Thermo Fisher Scientific, Middletown, VA) according to the manufacturer’s instructions. Quantified cholesterol values were normalized to tissue weight.

### Untargeted serum and liver metabolomic analyses

Serum and liver metabolites were extracted and subjected to LC-MS/MS detection on a Q Exactive HF-X Quadrupole-Orbitrap mass spectrometer system at Novogene (Sacramento, CA; n=6 mice per genotype per sex). Data were processed using Novogene in-house analysis pipeline. In brief, the raw mass spectrometry data were first converted to mzXML format using ProteoWizard (Chambers et al., 2012). Peak extraction, alignment, and retention time correction were then performed with XCMS software (Smith et al., 2006). The total peak area within each sample was normalized, and peaks with a missing rate greater than 50% across sample groups were filtered out. The corrected and filtered results were matched with the Novogene local database to obtain metabolite identification information. A total of 4182 metabolites were identified from the 48 samples. Multivariate statistical analysis was conducted on the metabolites, including Principal Component Analysis (PCA) and Partial Least Squares Discriminant Analysis (PLS-DA). PLS-DA is a supervised discriminant analysis statistical method. This method uses partial least squares regression (Boulesteix and Strimmer, 2007) to establish the relationship model between the relative quantitative value of metabolites and the sample category to realize the prediction of the sample category. The PLS-DA model of each comparison group was established, and the model evaluation parameters (R2, Q2) obtained by 7-cycle cross-validation. Differential metabolites were screened according to the criteria: VIP >1.0, fold change (FC)>1.2 or FC <0.833 and p-value <0.05. VIP refers to the variable importance in the projection of the first principal component of the PLS-DA model, and the VIP value represents the contribution of the metabolites to the grouping. KEGG enrichment (FDR correction by Benjamini and Hochberg method) and GSEA analysis was performed on KEGG entries based on the changes in quantitative values of metabolites. All metabolomics data, raw spectral files, and details of experimental protocol and data analyses have been deposited in a public repository, the Metabolomics Workbench (Sud et al., 2016).

### Mitochondrial respirometry

Respirometry was conducted on frozen tissue samples to assay for mitochondrial activity as described previously (Acin-Perez et al., 2020; Sarver et al., 2024). Briefly, liver and BAT were dissected, snapped frozen in liquid nitrogen, and stored at –80 °C for later analysis. Samples were thawed in MAS buffer (70 mM sucrose, 220 mM mannitol, 5 mM KH_2_PO_4_, 5 mM MgCl_2_, 1 mM EGTA, 2 mM HEPES pH 7.4), finely minced with scissors, and then homogenized with a glass Dounce homogenizer. The resulting homogenate was spun at 1000 × *g* for 10 min at 4 °C. The supernatant was collected and immediately used for protein quantification by BCA assay (Thermo Fisher Scientific, 23225). Each well of the Seahorse microplate was loaded with 4 µg (BAT) or 8 µg (liver) homogenate protein. Each biological replicate consists of three technical replicates. Samples from all tissues were treated separately with NADH (1 mM) as a complex I substrate or Succinate (a complex II substrate, 5 mM) in the presence of rotenone (a complex I inhibitor, 2 µM), then with the inhibitors rotenone (2 µM) and Antimycin A (4 µM), followed by TMPD (0.45 mM) and Ascorbate (1 mM) to activate complex IV, and finally treated with Azide (40 mM) to assess non-mitochondrial respiration. All mitochondrial respiration data were normalized to mitochondrial content, quantified using MitoTracker Deep Red (MTDR, Thermo Fisher, M22426) as described (Acin-Perez et al., 2020; Sarver et al., 2024). Briefly, lysates were incubated with MTDR (1 µM) for 10 min at 37 °C, then centrifuged at 2000 × *g* for 5 min at 4 °C. The supernatant was carefully removed and replaced with 1 x MAS solution and fluorescence was read with excitation and emission wavelengths of 625 and 670 nm, respectively. To minimize non-specific background signal contribution, control wells were loaded with MTDR and 1 x MAS and subtracted from all sample values.

### RNA-sequencing and bioinformatics analysis

Bulk RNA sequencing of Dp16 (n=6) and WT (n=6) mouse liver, gWAT, iWAT, BAT, skeletal muscle (gastrocnemius), pancreas, and hypothalamus were performed by Novogene (Sacramento, California, USA) on a NovaSeq X Plus platform and pair-end reads (2×150 bp) were generated, with 6 G raw data per sample. One gWAT sample from the Dp16 female mice failed the initial quality control test and was excluded from subsequent RNA sequencing. Sequencing data was analyzed using the standard Novogene Analysis Pipeline. Sequencing reads were aligned to *Mus musculus* reference genome (GRCm39/mm39). Data analysis was performed using a combination of programs, including Fastp, Hisat2, and FeatureCounts. Differential expressions were determined through DESeq2. The resulting p-values were adjusted using the Benjamini and Hochberg’s approach for controlling the false discovery rate. Genes with an adjusted p-value <0.05 and log2(FC)>0.5 were assigned as differentially expressed. GO, KEGG, and Reactome (http://www.reactome.org) enrichment were implemented by ClusterProfiler. All volcano plots and heat maps were generated in Graphpad Prism 10 software. All statistics were performed on log transformed data. All heat maps were generated from column z-score transformed data. The z-score of each column was determined by taking the column average, subtracting each sample’s individual expression value by said average then dividing that difference by the column standard deviation. Z-score = (value – column average)/column standard deviation. High-throughput sequencing data from this study have been submitted to the NCBI Sequence Read Archive (SRA) under accession number # PRJNA1160420.

### Statistical analyses

All results are expressed as mean ± SEM. Statistical analysis was performed with GraphPad Prism 10 software (GraphPad Software, San Diego, CA). Data were analyzed with two-tailed Student’s *t*-tests or by repeated measures ANOVA. For two-way ANOVA, we performed Sidek or Bonferroni post hoc tests. p<0.05 was considered statistically significant.

## Data Availability

All RNA-seq data have been deposited in NCBI Sequence Read Archive (SRA), with the accession # PRJNA1160420. The metabolomics data is available at the NIH Common Fund's National Metabolomics Data Repository (NMDR) website, the Metabolomics Workbench (https://www.metabolomicsworkbench.org) where it has been assigned Study ID (ST004905, ST004915, ST004916, and ST004918). The data can be accessed directly via it's Project DOI: https://doi.org/10.21228/M8SZ8Q. The following datasets were generated: ChenF SaqibM NguyenCM SarverDC YuYE AjaS SeldinMM WongGW 2024RNA-seq from multiple tissues from DP16 vs WT miceNCBI BioProjectPRJNA1160420 WongGW 2026Metabolomics analysis of serum samples from female wild-type (WT) and Dp16 Down syndrome miceNational Metabolomics Data RepositoryST004915 WongGW 2026Metabolomics analysis of serum samples from male wild-type (WT) and Dp16 Down syndrome miceNational Metabolomics Data RepositoryST004916 WongGW 2026Metabolomics analysis of liver samples from female wild-type (WT) and Dp16 Down syndrome miceNational Metabolomics Data RepositoryST004918 WongGW 2026Metabolomics analysis of liver samples from male wild-type (WT) and Dp16 Down syndrome miceNational Metabolomics Data RepositoryST004905 WongGW 2026Gene dosage imbalance disrupts systemic metabolism in the Dp16 Down syndrome mouse modelMetabolics Workbench10.21228/M8SZ8QPMC1343295142544450

## Additional files

MDAR checklist

## References

[bib1] Acin-Perez R, Benador IY, Petcherski A, Veliova M, Benavides GA, Lagarrigue S, Caudal A, Vergnes L, Murphy AN, Karamanlidis G, Tian R, Reue K, Wanagat J, Sacks H, Amati F, Darley-Usmar VM, Liesa M, Divakaruni AS, Stiles L, Shirihai OS (2020). A novel approach to measure mitochondrial respiration in frozen biological samples. The EMBO Journal.

[bib2] Adelekan T, Magge S, Shults J, Stallings V, Stettler N (2012). Lipid profiles of children with Down syndrome compared with their siblings. Pediatrics.

[bib3] Ahumada Saavedra JT, Chevalier C, Bloch Zupan A, Herault Y (2025). Ripply3 overdosage induces mid-face shortening through Tbx1 downregulation in Down syndrome models. PLOS Genetics.

[bib4] Allison DB, Gomez JE, Heshka S, Babbitt RL, Geliebter A, Kreibich K, Heymsfield SB (1995). Decreased resting metabolic rate among persons with Down Syndrome. International Journal of Obesity and Related Metabolic Disorders.

[bib5] Anderson CC, Marentette JO, Prutton KM, Rauniyar AK, Reisz JA, D’Alessandro A, Maclean KN, Saba LM, Roede JR (2021). Trisomy 21 results in modest impacts on mitochondrial function and central carbon metabolism. Free Radical Biology & Medicine.

[bib6] Antonarakis SE (2017). Down syndrome and the complexity of genome dosage imbalance. Nature Reviews. Genetics.

[bib7] Antonarakis SE, Skotko BG, Rafii MS, Strydom A, Pape SE, Bianchi DW, Sherman SL, Reeves RH (2020). Down syndrome. Nature Reviews. Disease Primers.

[bib8] Aslam AA, Baksh RA, Pape SE, Strydom A, Gulliford MC, Chan LF, GO-DS21 Consortium (2022). Diabetes and obesity in down syndrome across the lifespan: a retrospective cohort study using U.K. electronic health records. Diabetes Care.

[bib9] Bauters D, Spincemaille P, Geys L, Cassiman D, Vermeersch P, Bedossa P, Scroyen I, Lijnen HR (2016). ADAMTS5 deficiency protects against non-alcoholic steatohepatitis in obesity. Liver International.

[bib10] Bauters D, Bedossa P, Lijnen HR, Hemmeryckx B (2018). Functional role of ADAMTS5 in adiposity and metabolic health. PLOS ONE.

[bib11] Bell RMB, Villalobos E, Nixon M, Miguelez-Crespo A, Murphy L, Fawkes A, Coutts A, Sharp MGF, Koerner MV, Allan E, Meijer OC, Houtman R, Odermatt A, Beck KR, Denham SG, Lee P, Homer NZM, Walker BR, Morgan RA (2021). Carbonyl reductase 1 amplifies glucocorticoid action in adipose tissue and impairs glucose tolerance in lean mice. Molecular Metabolism.

[bib12] Bertapelli F, Pitetti K, Agiovlasitis S, Guerra-Junior G (2016). Overweight and obesity in children and adolescents with Down syndrome-prevalence, determinants, consequences, and interventions: a literature review. Research in Developmental Disabilities.

[bib13] Bertrand R, Tolu S, Picot D, Tourrel-Cuzin C, Ouahab A, Dairou J, Deau E, Lindberg MF, Meijer L, Movassat J, Uzan B (2025). DYRK1A inhibition restores pancreatic functions and improves glucose metabolism in a preclinical model of type 2 diabetes. Molecular Metabolism.

[bib14] Bhatti JS, Bhatti GK, Reddy PH (2017). Mitochondrial dysfunction and oxidative stress in metabolic disorders - A step towards mitochondria based therapeutic strategies. Biochimica et Biophysica Acta. Molecular Basis of Disease.

[bib15] Bhaumik S, Watson JM, Thorp CF, Tyrer F, McGrother CW (2008). Body mass index in adults with intellectual disability: distribution, associations and service implications: a population-based prevalence study. Journal of Intellectual Disability Research.

[bib16] Birsoy K, Berry R, Wang T, Ceyhan O, Tavazoie S, Friedman JM, Rodeheffer MS (2011). Analysis of gene networks in white adipose tissue development reveals a role for ETS2 in adipogenesis. Development.

[bib17] Blencowe M, Chen X, Zhao Y, Itoh Y, McQuillen CN, Han Y, Shou BL, McClusky R, Reue K, Arnold AP, Yang X (2022). Relative contributions of sex hormones, sex chromosomes, and gonads to sex differences in tissue gene regulation. Genome Research.

[bib18] Boulesteix AL, Strimmer K (2007). Partial least squares: a versatile tool for the analysis of high-dimensional genomic data. Briefings in Bioinformatics.

[bib19] Buonuomo PS, Bartuli A, Mastrogiorgio G, Vittucci A, Di Camillo C, Bianchi S, Pires Marafon D, Villani A, Valentini D (2016). Lipid profiles in a large cohort of italian children with down syndrome. European Journal of Medical Genetics.

[bib20] Chambers MC, Maclean B, Burke R, Amodei D, Ruderman DL, Neumann S, Gatto L, Fischer B, Pratt B, Egertson J, Hoff K, Kessner D, Tasman N, Shulman N, Frewen B, Baker TA, Brusniak MY, Paulse C, Creasy D, Flashner L, Kani K, Moulding C, Seymour SL, Nuwaysir LM, Lefebvre B, Kuhlmann F, Roark J, Rainer P, Detlev S, Hemenway T, Huhmer A, Langridge J, Connolly B, Chadick T, Holly K, Eckels J, Deutsch EW, Moritz RL, Katz JE, Agus DB, MacCoss M, Tabb DL, Mallick P (2012). A cross-platform toolkit for mass spectrometry and proteomics. Nature Biotechnology.

[bib21] Correa SM, Newstrom DW, Warne JP, Flandin P, Cheung CC, Lin-Moore AT, Pierce AA, Xu AW, Rubenstein JL, Ingraham HA (2015). An estrogen-responsive module in the ventromedial hypothalamus selectively drives sex-specific activity in females. Cell Reports.

[bib22] Davisson MT, Schmidt C, Akeson EC (1990). Segmental trisomy of murine chromosome 16: a new model system for studying Down syndrome. Progress in Clinical and Biological Research.

[bib23] Davisson MT, Schmidt C, Reeves RH, Irving NG, Akeson EC, Harris BS, Bronson RT (1993). Segmental trisomy as a mouse model for Down syndrome. Progress in Clinical and Biological Research.

[bib24] de la Piedra MJ, Alberti G, Cerda J, Cárdenas A, Paul MA, Lizama M (2017). High frequency of dyslipidemia in children and adolescents with Down Syndrome. Revista Chilena de Pediatria.

[bib25] Dennis EA, Norris PC (2015). Eicosanoid storm in infection and inflammation. Nature Reviews. Immunology.

[bib26] Dimopoulos K, Constantine A, Clift P, Condliffe R, Moledina S, Jansen K, Inuzuka R, Veldtman GR, Cua CL, Tay ELW, Opotowsky AR, Giannakoulas G, Alonso-Gonzalez R, Cordina R, Capone G, Namuyonga J, Scott CH, D’Alto M, Gamero FJ, Chicoine B, Gu H, Limsuwan A, Majekodunmi T, Budts W, Coghlan G, Broberg CS (2023). Cardiovascular complications of down syndrome. And for Down Syndrome.

[bib27] Donovan MG, Eduthan NP, Smith KP, Britton EC, Lyford HR, Araya P, Granrath RE, Waugh KA, Enriquez Estrada B, Rachubinski AL, Sullivan KD, Galbraith MD, Espinosa JM (2024). Variegated overexpression of chromosome 21 genes reveals molecular and immune subtypes of Down syndrome. Nature Communications.

[bib28] Duchon A, Raveau M, Chevalier C, Nalesso V, Sharp AJ, Herault Y (2011). Identification of the translocation breakpoints in the Ts65Dn and Ts1Cje mouse lines: relevance for modeling Down syndrome. Mammalian Genome.

[bib29] Duchon A, Del Mar Muniz Moreno M, Martin Lorenzo S, Silva de Souza MP, Chevalier C, Nalesso V, Meziane H, Loureiro de Sousa P, Noblet V, Armspach JP, Brault V, Herault Y (2021). Multi-influential genetic interactions alter behaviour and cognition through six main biological cascades in Down syndrome mouse models. Human Molecular Genetics.

[bib30] Duchon A, del Mar Muñiz Moreno M, Chevalier C, Nalesso V, Andre P, Fructuoso-Castellar M, Mondino M, Po C, Noblet V, Birling MC, Potier MC, Herault Y (2022). Ts66Yah, a mouse model of Down syndrome with improved construct and face validity. Disease Models & Mechanisms.

[bib31] Dunn LN, Niemeyer BF, Eduthan NP, Schade KA, Waugh KA, Brown C, Rachubinski AL, Timkovich AE, Orlicky DJ, Galbraith MD, Espinosa JM, Sullivan KD (2026). Altered hepatic metabolism in Down syndrome. Cell Reports.

[bib32] Fernhall B, Figueroa A, Collier S, Goulopoulou S, Giannopoulou I, Baynard T (2005). Resting metabolic rate is not reduced in obese adults with Down syndrome. Mental Retardation.

[bib33] Fleishman JS, Kumar S (2024). Bile acid metabolism and signaling in health and disease: molecular mechanisms and therapeutic targets. Signal Transduction and Targeted Therapy.

[bib34] Folch J, Lees M, Sloane Stanley GH (1957). A simple method for the isolation and purification of total lipides from animal tissues. The Journal of Biological Chemistry.

[bib35] Fonseca CT, Amaral DM, Ribeiro MG, Beserra ICR, Guimarães MM (2005). Insulin resistance in adolescents with Down syndrome: a cross-sectional study. BMC Endocrine Disorders.

[bib36] Fox B, Moffett GE, Kinnison C, Brooks G, Case LE (2019). Physical activity levels of children with down syndrome. Pediatric Physical Therapy.

[bib37] Fuchs CD, Simbrunner B, Baumgartner M, Campbell C, Reiberger T, Trauner M (2025). Bile acid metabolism and signalling in liver disease. Journal of Hepatology.

[bib38] Gan J, Pinto-Fernández A, Flierman D, Akkermans J, O’Brien DP, Greenwood H, Scott HC, Fritz G, Knobeloch KP, Neefjes J, van Dam H, Ovaa H, Ploegh HL, Kessler BM, Geurink PP, Sapmaz A (2023). USP16 is an ISG15 cross-reactive deubiquitinase that targets pro-ISG15 and ISGylated proteins involved in metabolism. PNAS.

[bib39] Garcia-de la Puente S, Flores-Arizmendi KA, Delgado-Montemayor MJ, Vargas-Robledo TT (2021). Lipid profile of mexican children with down syndrome. BMC Pediatrics.

[bib40] Gomez-Larrauri A, Larrea-Sebal A, Martín C, Gomez-Muñoz A (2025). The critical roles of bioactive sphingolipids in inflammation. The Journal of Biological Chemistry.

[bib41] Gomez-Sanchez CE, Holland OB, Murry BA, Lloyd HA, Milewich L (1979). 19-nor-deoxycorticosterone: a potent mineralcocorticoid isolated from the urine of rats with regenerating adrenals. Endocrinology.

[bib42] González-Agüero A, Ara I, Moreno LA, Vicente-Rodríguez G, Casajús JA (2011). Fat and lean masses in youths with Down syndrome: gender differences. Research in Developmental Disabilities.

[bib43] Gorsline J, Morris DJ (1985). The hypertensinogenic activity of 19-nor-deoxycorticosterone in the adrenalectomized spontaneously hypertensive rat. Journal of Steroid Biochemistry.

[bib44] Gross TJ, Doran E, Cheema AK, Head E, Lott IT, Mapstone M (2019). Plasma metabolites related to cellular energy metabolism are altered in adults with Down syndrome and Alzheimer’s disease. Developmental Neurobiology.

[bib45] Guedj F, Kane E, Bishop LA, Pennings JLA, Herault Y, Bianchi DW (2023). The impact of Mmu17 Non-Hsa21 orthologous genes in the Ts65Dn mouse model of down syndrome: the gold standard refuted. Biological Psychiatry.

[bib46] Gupta M, Dhanasekaran AR, Gardiner KJ (2016). Mouse models of Down syndrome: gene content and consequences. Mammalian Genome.

[bib47] Gutierrez-Hervas A, Gómez-Martínez S, Izquierdo-Gómez R, Veiga OL, Perez-Bey A, Castro-Piñero J, Marcos A (2020). Inflammation and fatness in adolescents with and without DOWN syndrome: UP & DOWN study. Journal of Intellectual Disability Research.

[bib48] Haydar TF, Reeves RH (2012). Trisomy 21 and early brain development. Trends in Neurosciences.

[bib49] Helguera P, Seiglie J, Rodriguez J, Hanna M, Helguera G, Busciglio J (2013). Adaptive downregulation of mitochondrial function in down syndrome. Cell Metabolism.

[bib50] Herault Y, Delabar JM, Fisher EMC, Tybulewicz VLJ, Yu E, Brault V (2017). Rodent models in Down syndrome research: impact and future opportunities. Disease Models & Mechanisms.

[bib51] Hevener AL, Correa SM (2025). Metabolic messengers: oestradiol. Nature Metabolism.

[bib52] Hill DL, Parks EP, Zemel BS, Shults J, Stallings VA, Stettler N (2013). Resting energy expenditure and adiposity accretion among children with Down syndrome: a 3-year prospective study. European Journal of Clinical Nutrition.

[bib53] Hotamisligil GS (2010). Endoplasmic reticulum stress and the inflammatory basis of metabolic disease. Cell.

[bib54] Hsieh K, Rimmer JH, Heller T (2014). Obesity and associated factors in adults with intellectual disability. Journal of Intellectual Disability Research.

[bib55] Huang YS, Huang WC, Li CW, Chuang LT (2011). Eicosadienoic acid differentially modulates production of pro-inflammatory modulators in murine macrophages. Molecular and Cellular Biochemistry.

[bib56] Hunter S, Hendrix J, Freeman J, Dowell RD, Allen MA (2023). Transcription dosage compensation does not occur in Down syndrome. BMC Biology.

[bib57] Izzo A, Manco R, Bonfiglio F, Calì G, De Cristofaro T, Patergnani S, Cicatiello R, Scrima R, Zannini M, Pinton P, Conti A, Nitsch L (2014). NRIP1/RIP140 siRNA-mediated attenuation counteracts mitochondrial dysfunction in Down syndrome. Human Molecular Genetics.

[bib58] Izzo A, Nitti M, Mollo N, Paladino S, Procaccini C, Faicchia D, Calì G, Genesio R, Bonfiglio F, Cicatiello R, Polishchuk E, Polishchuk R, Pinton P, Matarese G, Conti A, Nitsch L (2017). Metformin restores the mitochondrial network and reverses mitochondrial dysfunction in Down syndrome cells. Human Molecular Genetics.

[bib59] Jiang X, Liu C, Yu T, Zhang L, Meng K, Xing Z, Belichenko PV, Kleschevnikov AM, Pao A, Peresie J, Wie S, Mobley WC, Yu YE (2015). Genetic dissection of the Down syndrome critical region. Human Molecular Genetics.

[bib60] Jin J, He Y, Guo J, Pan Q, Wei X, Xu C, Qi Z, Li Q, Ma S, Lin J, Jiang N, Ma J, Wang X, Jiang L, Ding Q, Osto E, Zhi X, Meng D (2023). BACH1 controls hepatic insulin signaling and glucose homeostasis in mice. Nature Communications.

[bib61] Kazuki Y, Gao FJ, Li Y, Moyer AJ, Devenney B, Hiramatsu K, Miyagawa-Tomita S, Abe S, Kazuki K, Kajitani N, Uno N, Takehara S, Takiguchi M, Yamakawa M, Hasegawa A, Shimizu R, Matsukura S, Noda N, Ogonuki N, Inoue K, Matoba S, Ogura A, Florea LD, Savonenko A, Xiao M, Wu D, Batista DA, Yang J, Qiu Z, Singh N, Richtsmeier JT, Takeuchi T, Oshimura M, Reeves RH (2020). A non-mosaic transchromosomic mouse model of down syndrome carrying the long arm of human chromosome 21. eLife.

[bib62] Kelly DM, Jones TH (2013). Testosterone: a metabolic hormone in health and disease. The Journal of Endocrinology.

[bib63] Kilbey A, Terry A, Wotton S, Borland G, Zhang Q, Mackay N, McDonald A, Bell M, Wakelam MJO, Cameron ER, Neil JC (2017). Runx1 orchestrates sphingolipid metabolism and glucocorticoid resistance in lymphomagenesis. Journal of Cellular Biochemistry.

[bib64] Korbel JO, Tirosh-Wagner T, Urban AE, Chen X-N, Kasowski M, Dai L, Grubert F, Erdman C, Gao MC, Lange K, Sobel EM, Barlow GM, Aylsworth AS, Carpenter NJ, Clark RD, Cohen MY, Doran E, Falik-Zaccai T, Lewin SO, Lott IT, McGillivray BC, Moeschler JB, Pettenati MJ, Pueschel SM, Rao KW, Shaffer LG, Shohat M, Van Riper AJ, Warburton D, Weissman S, Gerstein MB, Snyder M, Korenberg JR (2009). The genetic architecture of Down syndrome phenotypes revealed by high-resolution analysis of human segmental trisomies. PNAS.

[bib65] Korenberg JR, Chen XN, Schipper R, Sun Z, Gonsky R, Gerwehr S, Carpenter N, Daumer C, Dignan P, Disteche C (1994). Down syndrome phenotypes: the consequences of chromosomal imbalance. PNAS.

[bib66] Kowluru RA, Kowluru A, Veluthakal R, Mohammad G, Syed I, Santos JM, Mishra M (2014). TIAM1-RAC1 signalling axis-mediated activation of NADPH oxidase-2 initiates mitochondrial damage in the development of diabetic retinopathy. Diabetologia.

[bib67] Koyama Y, Brenner DA (2017). Liver inflammation and fibrosis. The Journal of Clinical Investigation.

[bib68] Krajewski-Hall SJ, Blackmore EM, McMinn JR, Rance NE (2018). Estradiol alters body temperature regulation in the female mouse. Temperature.

[bib69] Krause WC, Rodriguez R, Gegenhuber B, Matharu N, Rodriguez AN, Padilla-Roger AM, Toma K, Herber CB, Correa SM, Duan X, Ahituv N, Tollkuhn J, Ingraham HA (2021). Oestrogen engages brain MC4R signalling to drive physical activity in female mice. Nature.

[bib70] Kuda O, Brezinova M, Rombaldova M, Slavikova B, Posta M, Beier P, Janovska P, Veleba J, Kopecky J, Kudova E, Pelikanova T, Kopecky J (2016). Docosahexaenoic acid-derived fatty acid esters of hydroxy fatty acids (FAHFAs) with anti-inflammatory properties. Diabetes.

[bib71] LaCombe JM, Roper RJ (2020). Skeletal dynamics of down syndrome: a developing perspective. Bone.

[bib72] Lana-Elola E, Watson-Scales S, Slender A, Gibbins D, Martineau A, Douglas C, Mohun T, Fisher EM, Tybulewicz VL (2016). Genetic dissection of Down syndrome-associated congenital heart defects using a new mouse mapping panel. eLife.

[bib73] Lei X, Wong GW (2019). C1q/TNF-related protein 2 (CTRP2) deletion promotes adipose tissue lipolysis and hepatic triglyceride secretion. The Journal of Biological Chemistry.

[bib74] Li Z, Yu T, Morishima M, Pao A, LaDuca J, Conroy J, Nowak N, Matsui SI, Shiraishi I, Yu YE (2007). Duplication of the entire 22.9 Mb human chromosome 21 syntenic region on mouse chromosome 16 causes cardiovascular and gastrointestinal abnormalities. Human Molecular Genetics.

[bib75] Li X, Liu Z, Luo C, Jia H, Sun L, Hou B, Shen W, Packer L, Cotman CW, Liu J (2008). Lipoamide protects retinal pigment epithelial cells from oxidative stress and mitochondrial dysfunction. Free Radical Biology & Medicine.

[bib76] Li H, Edie S, Klinedinst D, Jeong JS, Blackshaw S, Maslen CL, Reeves RH (2016). Penetrance of congenital heart disease in a mouse model of down syndrome depends on a trisomic potentiator of a disomic modifier. Genetics.

[bib77] Li Y, Xing Z, Yu T, Pao A, Daadi M, Yu YE (2021). Coat color-facilitated efficient generation and analysis of a mouse model of down syndrome triplicated for all human chromosome 21 orthologous regions. Genes.

[bib78] Liu Y, Beyer A, Aebersold R (2016). On the dependency of cellular protein levels on mRNA abundance. Cell.

[bib79] Liu Y, Borel C, Li L, Müller T, Williams EG, Germain PL, Buljan M, Sajic T, Boersema PJ, Shao W, Faini M, Testa G, Beyer A, Antonarakis SE, Aebersold R (2017). Systematic proteome and proteostasis profiling in human Trisomy 21 fibroblast cells. Nature Communications.

[bib80] Luke A, Sutton M, Schoeller DA, Roizen NJ (1996). Nutrient intake and obesity in prepubescent children with Down syndrome. Journal of the American Dietetic Association.

[bib81] Magenis ML, Machado AG, Bongiolo AM, Silva M da, Castro K, Perry IDS (2018). Dietary practices of children and adolescents with Down syndrome. Journal of Intellectual Disabilities.

[bib82] Magge SN, Zemel BS, Pipan ME, Gidding SS, Kelly A (2019). Cardiometabolic risk and body composition in youth with down syndrome. Pediatrics.

[bib83] Milunsky A, Neurath PW (1968). Diabetes mellitus in Down’s Syndrome. Archives of Environmental Health.

[bib84] Mirhafez SR, Avan A, Pasdar A, Khatamianfar S, Hosseinzadeh L, Ganjali S, Movahedi A, Pirhoushiaran M, Mellado VG, Rosace D, van Krieken A, Nohtani M, Ferns GA, Ghayour-Mobarhan M (2016). Zinc finger 259 gene polymorphism rs964184 is associated with serum triglyceride levels and metabolic syndrome. International Journal of Molecular and Cellular Medicine.

[bib85] Mollo N, Esposito M, Aurilia M, Scognamiglio R, Accarino R, Bonfiglio F, Cicatiello R, Charalambous M, Procaccini C, Micillo T, Genesio R, Calì G, Secondo A, Paladino S, Matarese G, De Vita G, Conti A, Nitsch L, Izzo A (2021). Human trisomic iPSCs from down syndrome fibroblasts manifest mitochondrial alterations early during neuronal differentiation. Biology.

[bib86] Mzoughi S, Fong JY, Papadopoli D, Koh CM, Hulea L, Pigini P, Di Tullio F, Andreacchio G, Hoppe MM, Wollmann H, Low D, Caldez MJ, Peng Y, Torre D, Zhao JN, Uchenunu O, Varano G, Motofeanu CM, Lakshmanan M, Teo SX, Wun CM, Perini G, Tan SY, Ong CB, Al-Haddawi M, Rajarethinam R, Hue SSS, Lim ST, Ong CK, Huang D, Ng SB, Bernstein E, Hasson D, Wee KB, Kaldis P, Jeyasekharan A, Dominguez-Sola D, Topisirovic I, Guccione E (2020). PRDM15 is a key regulator of metabolism critical to sustain B-cell lymphomagenesis. Nature Communications.

[bib87] Nanduri R, Furusawa T, Lobanov A, He B, Xie C, Dadkhah K, Kelly MC, Gavrilova O, Gonzalez FJ, Bustin M (2022). Epigenetic regulation of white adipose tissue plasticity and energy metabolism by nucleosome binding HMGN proteins. Nature Communications.

[bib88] O’Donnell VB, Rossjohn J, Wakelam MJ (2018). Phospholipid signaling in innate immune cells. The Journal of Clinical Investigation.

[bib89] Panagaki T, Randi EB, Augsburger F, Szabo C (2019). Overproduction of H_2_S, generated by CBS, inhibits mitochondrial Complex IV and suppresses oxidative phosphorylation in Down syndrome. PNAS.

[bib90] Parra V, Altamirano F, Hernández-Fuentes CP, Tong D, Kyrychenko V, Rotter D, Pedrozo Z, Hill JA, Eisner V, Lavandero S, Schneider JW, Rothermel BA (2018). Down syndrome critical region 1 Gene, *Rcan1*, helps maintain a more fused mitochondrial network. Circulation Research.

[bib91] Peiris H, Raghupathi R, Jessup CF, Zanin MP, Mohanasundaram D, Mackenzie KD, Chataway T, Clarke JN, Brealey J, Coates PT, Pritchard MA, Keating DJ (2012). Increased expression of the glucose-responsive gene, RCAN1, causes hypoinsulinemia, β-cell dysfunction, and diabetes. Endocrinology.

[bib92] Peiris H, Duffield MD, Fadista J, Jessup CF, Kashmir V, Genders AJ, McGee SL, Martin AM, Saiedi M, Morton N, Carter R, Cousin MA, Kokotos AC, Oskolkov N, Volkov P, Hough TA, Fisher EMC, Tybulewicz VLJ, Busciglio J, Coskun PE, Becker A, Belichenko PV, Mobley WC, Ryan MT, Chan JY, Laybutt DR, Coates PT, Yang S, Ling C, Groop L, Pritchard MA, Keating DJ (2016). A syntenic cross species aneuploidy genetic screen links RCAN1 expression to β-cell mitochondrial dysfunction in type 2 Diabetes. PLOS Genetics.

[bib93] Pereira PL, Magnol L, Sahún I, Brault V, Duchon A, Prandini P, Gruart A, Bizot JC, Chadefaux-Vekemans B, Deutsch S, Trovero F, Delgado-García JM, Antonarakis SE, Dierssen M, Herault Y (2009). A new mouse model for the trisomy of the Abcg1-U2af1 region reveals the complexity of the combinatorial genetic code of down syndrome. Human Molecular Genetics.

[bib94] Perino A, Demagny H, Velazquez-Villegas L, Schoonjans K (2021). Molecular physiology of bile acid signaling in health, disease, and aging. Physiological Reviews.

[bib95] Phillips AC, Sleigh A, McAllister CJ, Brage S, Carpenter TA, Kemp GJ, Holland AJ (2013). Defective mitochondrial function in vivo in skeletal muscle in adults with Down’s syndrome: a 31P-MRS study. PLOS ONE.

[bib96] Piccoli C, Izzo A, Scrima R, Bonfiglio F, Manco R, Negri R, Quarato G, Cela O, Ripoli M, Prisco M, Gentile F, Calì G, Pinton P, Conti A, Nitsch L, Capitanio N (2013). Chronic pro-oxidative state and mitochondrial dysfunctions are more pronounced in fibroblasts from Down syndrome foeti with congenital heart defects. Human Molecular Genetics.

[bib97] Pierce M, Ramsey K, Pinter J (2019). Trends in obesity and overweight in oregon children with down syndrome. Global Pediatric Health.

[bib98] Powers RK, Culp-Hill R, Ludwig MP, Smith KP, Waugh KA, Minter R, Tuttle KD, Lewis HC, Rachubinski AL, Granrath RE, Carmona-Iragui M, Wilkerson RB, Kahn DE, Joshi M, Lleó A, Blesa R, Fortea J, D’Alessandro A, Costello JC, Sullivan KD, Espinosa JM (2019). Trisomy 21 activates the kynurenine pathway via increased dosage of interferon receptors. Nature Communications.

[bib99] Ptomey LT, Willis EA, Sherman JR, White DA, Donnelly JE (2020). Exploring the effectiveness of an 18-month weight management intervention in adults with Down syndrome using propensity score matching. Journal of Intellectual Disability Research.

[bib100] Rajkovic Vuletic P, Geets-Kesic M, Jurcev-Savicevic A, Nurjanah N, Gilic B (2025). Metabolic, hematological, and functional health in adults with down syndrome and significance of parental health literacy: a cross-sectional study. Healthcare.

[bib101] Real de Asua D, Parra P, Costa R, Moldenhauer F, Suarez C (2014). Evaluation of the impact of abdominal obesity on glucose and lipid metabolism disorders in adults with Down syndrome. Research in Developmental Disabilities.

[bib102] Reeves RH, Irving NG, Moran TH, Wohn A, Kitt C, Sisodia SS, Schmidt C, Bronson RT, Davisson MT (1995). A mouse model for Down syndrome exhibits learning and behaviour deficits. Nature Genetics.

[bib103] Reinholdt LG, Ding Y, Gilbert GJ, Czechanski A, Solzak JP, Roper RJ, Johnson MT, Donahue LR, Lutz C, Davisson MT (2011). Molecular characterization of the translocation breakpoints in the Down syndrome mouse model Ts65Dn. Mammalian Genome.

[bib104] Ren N, Zhang H, Li T, Ji H, Zhang Z, Wu H (2024). ATP5J regulates microglial activation via mitochondrial dysfunction, exacerbating neuroinflammation in intracerebral hemorrhage. Frontiers in Immunology.

[bib105] Ring A, Pohl J, Völkl A, Stremmel W (2002). Evidence for vesicles that mediate long-chain fatty acid uptake by human microvascular endothelial cells. Journal of Lipid Research.

[bib106] Robbins JM, Benson M, Verkerke ARP, Tiwari G, Deng S, Rao P, Tahir UA, Avila-Pacheco J, Shi X, Guan Y, Tendoh FG, Barber JL, Miller PE, Perry AS, Hall ME, Wood AC, Taylor KD, Post WS, Rich SS, Nayor M, Wilson JG, Lewis GD, Shah RV, Rotter JI, Summers SA, Raffield LM, Kajimura S, Bouchard C, Clish CB, Sarzynski MA, Gerszten RE (2026). N-palmitoyl glutamine is a candidate mediator of cardiorespiratory fitness. Circulation.

[bib107] Roberts LJ, Morrow JD (2000). Measurement of F(2)-isoprostanes as an index of oxidative stress in vivo. Free Radical Biology & Medicine.

[bib108] Rodriguez S, Lei X, Petersen PS, Tan SY, Little HC, Wong GW (2016). Loss of CTRP1 disrupts glucose and lipid homeostasis. American Journal of Physiology. Endocrinology and Metabolism.

[bib109] Rönn T, Poulsen P, Tuomi T, Isomaa B, Groop L, Vaag A, Ling C (2009). Genetic variation in ATP5O is associated with skeletal muscle ATP50 mRNA expression and glucose uptake in young twins. PLOS ONE.

[bib110] Sarver DC, Stewart AN, Rodriguez S, Little HC, Aja S, Wong GW (2020). Loss of CTRP4 alters adiposity and food intake behaviors in obese mice. American Journal of Physiology. Endocrinology and Metabolism.

[bib111] Sarver DC, Xu C, Rodriguez S, Aja S, Jaffe AE, Gao FJ, Delannoy M, Periasamy M, Kazuki Y, Oshimura M, Reeves RH, Wong GW (2023a). Hypermetabolism in mice carrying a near-complete human chromosome 21. eLife.

[bib112] Sarver DC, Xu C, Velez LM, Aja S, Jaffe AE, Seldin MM, Reeves RH, Wong GW (2023b). Dysregulated systemic metabolism in a Down syndrome mouse model. Molecular Metabolism.

[bib113] Sarver DC, Saqib M, Chen F, Wong GW (2024). Mitochondrial respiration atlas reveals differential changes in mitochondrial function across sex and age. eLife.

[bib114] Serhan CN, Levy BD (2018). Resolvins in inflammation: emergence of the pro-resolving superfamily of mediators. The Journal of Clinical Investigation.

[bib115] Sherman SL, Allen EG, Bean LH, Freeman SB (2007). Epidemiology of Down syndrome. Mental Retardation and Developmental Disabilities Research Reviews.

[bib116] Sloan K, Thomas J, Blackwell M, Voisard D, Lana-Elola E, Watson-Scales S, Roper DL, Wallace JM, Fisher EMC, Tybulewicz VLJ, Roper RJ (2023). Genetic dissection of triplicated chromosome 21 orthologs yields varying skeletal traits in Down syndrome model mice. Disease Models & Mechanisms.

[bib117] Smith CA, Want EJ, O’Maille G, Abagyan R, Siuzdak G (2006). XCMS: processing mass spectrometry data for metabolite profiling using nonlinear peak alignment, matching, and identification. Analytical Chemistry.

[bib118] Sud M, Fahy E, Cotter D, Azam K, Vadivelu I, Burant C, Edison A, Fiehn O, Higashi R, Nair KS, Sumner S, Subramaniam S (2016). Metabolomics workbench: an international repository for metabolomics data and metadata, metabolite standards, protocols, tutorials and training, and analysis tools. Nucleic Acids Research.

[bib119] Sullivan KD, Lewis HC, Hill AA, Pandey A, Jackson LP, Cabral JM, Smith KP, Liggett LA, Gomez EB, Galbraith MD, DeGregori J, Espinosa JM (2016). Trisomy 21 consistently activates the interferon response. eLife.

[bib120] Sun K, Tordjman J, Clément K, Scherer PE (2013). Fibrosis and adipose tissue dysfunction. Cell Metabolism.

[bib121] Syed I, Jayaram B, Subasinghe W, Kowluru A (2010). Tiam1/Rac1 signaling pathway mediates palmitate-induced, ceramide-sensitive generation of superoxides and lipid peroxides and the loss of mitochondrial membrane potential in pancreatic beta-cells. Biochemical Pharmacology.

[bib122] Tan SY, Little HC, Lei X, Li S, Rodriguez S, Wong GW (2016). Partial deficiency of CTRP12 alters hepatic lipid metabolism. Physiological Genomics.

[bib123] Tolu S, Hamzé R, Moreau M, Bertrand R, Janel N, Movassat J (2025). Beta cell function and global glucose metabolism are impaired in Dp(16)1Yey mouse model of Down syndrome. Diabetes, Obesity & Metabolism.

[bib124] Tsagkaraki E, Guilherme A, Nicoloro SM, Kelly M, Lifshitz LM, Wang H, Min K, Rowland LA, Santos KB, Wetoska N, Friedline RH, Maitland SA, Chen M, Weinstein LS, Wolfe SA, Kim JK, Czech MP (2023). Crosstalk between corepressor NRIP1 and cAMP signaling on adipocyte thermogenic programming. Molecular Metabolism.

[bib125] Tschöp MH, Speakman JR, Arch JRS, Auwerx J, Brüning JC, Chan L, Eckel RH, Farese RV, Galgani JE, Hambly C, Herman MA, Horvath TL, Kahn BB, Kozma SC, Maratos-Flier E, Müller TD, Münzberg H, Pfluger PT, Plum L, Reitman ML, Rahmouni K, Shulman GI, Thomas G, Kahn CR, Ravussin E (2012). A guide to analysis of mouse energy metabolism. Nature Methods.

[bib126] Valenti D, Tullo A, Caratozzolo MF, Merafina RS, Scartezzini P, Marra E, Vacca RA (2010). Impairment of F1F0-ATPase, adenine nucleotide translocator and adenylate kinase causes mitochondrial energy deficit in human skin fibroblasts with chromosome 21 trisomy. The Biochemical Journal.

[bib127] Valenti D, Manente GA, Moro L, Marra E, Vacca RA (2011). Deficit of complex I activity in human skin fibroblasts with chromosome 21 trisomy and overproduction of reactive oxygen species by mitochondria: involvement of the cAMP/PKA signalling pathway. The Biochemical Journal.

[bib128] Valentini D, Alisi A, di Camillo C, Sartorelli MR, Crudele A, Bartuli A, Nobili V, Villani A (2017). Nonalcoholic fatty liver disease in italian children with down syndrome: Prevalence and correlation with obesity-related features. The Journal of Pediatrics.

[bib129] Van Goor JC, Massa GG, Hirasing R (1997). Increased incidence and prevalence of diabetes mellitus in Down’s syndrome. Archives of Disease in Childhood.

[bib130] Wall SW, Sanchez L, Tuttle KS, Pearson SJ, Soma S, Wyatt GL, Carter HN, Jenschke RM, Tan L, Martinez SA, Lorenzi PL, Gohil VM, Rijnkels M, Porter WW (2023). Noncanonical role of singleminded-2s in mitochondrial respiratory chain formation in breast cancer. Experimental & Molecular Medicine.

[bib131] Wang Y, Xu H, Zhou X, Chen W, Zhou H (2024). Dysregulated bile acid homeostasis: unveiling its role in metabolic diseases. Medical Review.

[bib132] Waugh KA, Minter R, Baxter J, Chi C, Galbraith MD, Tuttle KD, Eduthan NP, Kinning KT, Andrysik Z, Araya P, Dougherty H, Dunn LN, Ludwig M, Schade KA, Tracy D, Smith KP, Granrath RE, Busquet N, Khanal S, Anderson RD, Cox LL, Estrada BE, Rachubinski AL, Lyford HR, Britton EC, Fantauzzo KA, Orlicky DJ, Matsuda JL, Song K, Cox TC, Sullivan KD, Espinosa JM (2023). Triplication of the interferon receptor locus contributes to hallmarks of Down syndrome in a mouse model. Nature Genetics.

[bib133] Wei WJ, Lin C, Luo SH, Huang JJ, Hu RT, Li JX, Ma HZ, Wei JZ, Wang CF, Lim CSY, Deng YB (2025). FAM3B activates hepatic stellate cells to accelerate hepatic fibrosis by promoting glucose metabolism. Biochemical and Biophysical Research Communications.

[bib134] Xing Z, Li Y, Cortes-Gomez E, Jiang X, Gao S, Pao A, Shan J, Song Y, Perez A, Yu T, Highsmith MR, Boadu F, Conroy JM, Singh PK, Bakin AV, Cheng J, Duan Z, Wang J, Liu S, Tycko B, Yu YE (2023). Dissection of a Down syndrome-associated trisomy to separate the gene dosage-dependent and -independent effects of an extra chromosome. Human Molecular Genetics.

[bib135] Xu L, Huo HQ, Lu KQ, Tang XY, Hong Y, Han X, Fu ZX, Fang KH, Xu M, Guo X, Liu Y (2022). Abnormal mitochondria in Down syndrome iPSC-derived GABAergic interneurons and organoids. Biochimica et Biophysica Acta. Molecular Basis of Disease.

[bib136] Yore MM, Syed I, Moraes-Vieira PM, Zhang T, Herman MA, Homan EA, Patel RT, Lee J, Chen S, Peroni OD, Dhaneshwar AS, Hammarstedt A, Smith U, McGraw TE, Saghatelian A, Kahn BB (2014). Discovery of a class of endogenous mammalian lipids with anti-diabetic and anti-inflammatory effects. Cell.

[bib137] Yu T, Li Z, Jia Z, Clapcote SJ, Liu C, Li S, Asrar S, Pao A, Chen R, Fan N, Carattini-Rivera S, Bechard AR, Spring S, Henkelman RM, Stoica G, Matsui SI, Nowak NJ, Roder JC, Chen C, Bradley A, Yu YE (2010). A mouse model of Down syndrome trisomic for all human chromosome 21 syntenic regions. Human Molecular Genetics.

[bib138] Zhang Z, Reis F, He Y, Park JW, DiVittorio JR, Sivakumar N, van Veen JE, Maesta-Pereira S, Shum M, Nichols I, Massa MG, Anderson S, Paul K, Liesa M, Ajijola OA, Xu Y, Adhikari A, Correa SM (2020). Estrogen-sensitive medial preoptic area neurons coordinate torpor in mice. Nature Communications.

[bib139] Zhang J, Wei Y, Yue Y, Jiao H, Wu Y, Fu W, Lin KM, Lu C, Mou S, Zhong Q (2024). RIPK4 promotes oxidative stress and ferroptotic death through the downregulation of ACSM1. PNAS.

[bib140] Zhu J, Tsai HJ, Gordon MR, Li R (2018). Cellular stress associated with aneuploidy. Developmental Cell.

