# Supplementary material for: Gene dosage imbalance disrupts systemic metabolism in the Dp16 Down syndrome mouse model
Source: eLife. 2026 Aug 3;15:RP110476. doi: 10.7554/eLife.110476 (PMC13432951; doi:10.7554/eLife.110476)
Supplement: Peer review history

# Editor report: eLife Assessment

**DOI:** 10.7554/eLife.110476.3.sa0

**Authors:** Jonathan S Bogan

**Affiliations:** Yale University United States

**Keywords:** Compelling, Important

This article describes the comprehensive metabolic phenotype of a mouse model of Down Syndrome, together with supporting transcriptomic, metabolomic, and biochemical data. The evidence presented is **compelling** and highlights several core phenotypes including insulin resistance, dyslipidemia, and tissue signatures indicating inflammatory and cellular stress pathways. Similarities and differences in male and female mice are highlighted. This **important** study provides essential groundwork for the further genetic dissection of dosage-sensitive genes causing metabolic dysregulation in Down Syndrome.

# Referee report: Reviewer #1 (Public review)

**DOI:** 10.7554/eLife.110476.3.sa1

[Editors' note: this version has been assessed by the Reviewing Editor without further input from the original reviewers. The authors have addressed the comments raised in the previous round of review.]

Summary:

Chen et al. describe metabolic phenotypes in Dp16 Down Syndrome mice, specifically the Dp(16)1Yey/+ mice - segmental duplication model carrying a majority of the triplicated Hsa21 gene orthologs. The group has performed metabolic phenotyping data in chow and high-fat diets, as well as undertaking a transcriptomic and metabolomic approach in tissues such as white and brown adipose tissues, liver, skeletal muscle, and hypothalamus to reveal both shared and sex-specific differences. The group describes sexual dimorphism in body weight, body temperature, food intake, and physical activity. Core shared features are insulin resistance, glucose intolerance, impaired lipid clearance, and dyslipidaemia in the Dp16 mice. They report tissue signatures of immune activation and a pro-inflammatory state, ER and oxidative stress, fibrosis, impaired glucose and fatty acid catabolism, altered lipid and bile acid profiles, and reduced mitochondrial respiration in Dp16 mice.

Strengths:

Overall, this is a good study with detailed, comprehensive data from an excellent group who have previously published on metabolic phenotyping of 2 other Down Syndrome mouse models. Although somewhat descriptive, it does certainly add to the current field and understanding of strengths and weaknesses of Down Syndrome mouse models, as well as identifying new features whilst strengthening previously suggested mechanisms.

# Referee report: Reviewer #2 (Public review)

**DOI:** 10.7554/eLife.110476.3.sa2

Summary:

Human DS is associated with metabolic dysfunction in humans, but the precise details of this have not been studied in detail. Here, the authors use a mouse model of DS to study systemic metabolic and transcriptional responses in key metabolic tissues to provide a deep understanding of the metabolic changes associated with DS. As part of his work, the authors also aimed to help inform the selection of a mouse model that best reflects the metabolic profile of DS, through comparison with other DS model metabolic data.

The data presented in this model will be of interest to those in the field of metabolism. The immediate impact is unclear, but the breadth of data presented makes this a very useful resource.

Strengths:

(1) This work builds on other comprehensive analyses that the authors have performed in other DS mouse models.

(2) The authors note common metabolic disturbances between male and female mice (e.g., insulin resistance) alongside clearly sexually dimorphic phenotypes (e.g., body weight). Studying both sexes in this context is important.

(3) The authors have written the paper in a way that integrates a large number of observations well. There is complex data, and a high degree of sexual dimorphism. The study has generated a valuable and wide-ranging dataset comprising molecular, biochemical, and physiological data that will be useful for further, more mechanistic studies of metabolism in DS.

(4) For specific observations, like the findings of altered body temperature in male and female mice, the authors undertake follow-up hypothesis-driven analyses of BAT mitochondria and specific hormones. Although these analyses do not explain the change in temperature, they ensure the study is not purely descriptive in nature.

# Referee report: Reviewer #3 (Public review)

**DOI:** 10.7554/eLife.110476.3.sa3

Summary:

The article by Chen et al. describes the comprehensive metabolic profiling of DP16 mice, a Down syndrome model that carries a duplicated segment of the mouse chromosome syntenic to human chromosome 21. The authors note that this model is superior to previously used models, based on genetics, as ~65% of the chromosome 21 orthologues. The metabolic phenotypes also appear to be more consistent with those observed in humans with Down Syndrome. The study lays the groundwork for a more detailed genetic dissection of dosage-sensitive genes that contribute to the metabolic deficits observed in Down Syndrome.

Strengths:

There is an enormous amount of data in this manuscript, and the methods are described with adequate attention to detail. A strength of the manuscript is that both male and female mice were analyzed, so that concordant and discordant phenotypes were identified. Both males and females had evidence of insulin resistance. Transcriptomic and metabolomic data revealed impaired pathways for lipid metabolism, a pro-inflammatory state, reduced mitochondrial health and oxidative stress. Although the effects of a high-fat diet on weight gain were divergent, this diet caused worsened insulin resistance in both males and females.

The discussion is excellent. Limitations of the study are well described. This reviewer does not identify any critical missing data.

# Author comment: Author response

**DOI:** 10.7554/eLife.110476.3.sa4

**Authors:** Fangluo Chen, Muzna Saqib, Christy M Nguyen, Dylan C Sarver, Yuejin Eugene Yu, Susan Aja, Marcus M Seldin, G William Wong

**Affiliations:** Johns Hopkins University Baltimore United States; Johns Hopkins University Baltimore United States; University of California, Irvine Irvine United States; Johns Hopkins University Baltimore United States; Roswell Park Comprehensive Cancer Center Buffalo United States; Johns Hopkins University School of Medicine Baltimore United States; University of California, Irvine Irvine United States; Johns Hopkins University Baltimore United States

**Public Reviews:**

**Reviewer #1 (Public review):**

Summary:

Chen et al. describe metabolic phenotypes in Dp16 Down Syndrome mice, specifically the Dp(16)1Yey/+ mice - segmental duplication model carrying a majority of the triplicated Hsa21 gene orthologs. The group has performed metabolic phenotyping data in chow and high-fat diets, as well as undertaking a transcriptomic and metabolomic approach in tissues such as white and brown adipose tissues, liver, skeletal muscle, and hypothalamus to reveal both shared and sex-specific differences. The group describes sexual dimorphism in body weight, body temperature, food intake, and physical activity. Core shared features are insulin resistance, glucose intolerance, impaired lipid clearance, and dyslipidaemia in the Dp16 mice. They report tissue signatures of immune activation and a pro-inflammatory state, ER and oxidative stress, fibrosis, impaired glucose and fatty acid catabolism, altered lipid and bile acid profiles, and reduced mitochondrial respiration in Dp16 mice.

Strengths:

Overall, this is a good study with detailed, comprehensive data from an excellent group who have previously published on metabolic phenotyping of 2 other Down Syndrome mouse models. Although somewhat descriptive, it does certainly add to the current field and understanding of strengths and weaknesses of Down Syndrome mouse models, as well as identifying new features whilst strengthening previously suggested mechanisms.

Weaknesses:

Many aspects of this study have been described in other Down syndrome mouse models, though there are certainly aspects that are new. It would be useful if the authors could do a direct critique and comparison with previous publications in the area, utilizing the same Down Syndrome mouse model. There are also a few limitations in the number of animals used and the interpretation of the data that should be acknowledged.

We have cited all relevant publications using Down syndrome mouse models. Regarding the Dp16 model, we have cited and discussed the only other study addressing metabolic aspects beyond body weight (Reference #138; PMID: 39803786). While that study reported glucose intolerance, insulin resistance, and defective insulin secretion, we did not measure pancreatic insulin content in our mice. Crucially, while the previous study found no sexual dimorphism, our study observed extensive sexual dimorphism in body weight gain, tissue-specific gene expression, and serum and liver metabolite changes.

Regarding sample size, we used 6 mice per genotype per sex for transcriptomic and metabolomic analyses; this is constrained by the cost of performing these omics-type analyses. For mitochondrial respiration assays, we used 9–10 mice, and for most other in vivo and ex vivo assays, we utilized 12–15 mice, with some assays exceeding 20. We believe these sample sizes are robust and appropriate for this study.

**Reviewer #2 (Public review):**

Summary:

Human DS is associated with metabolic dysfunction in humans, but the precise details of this have not been studied in detail. Here, the authors use a mouse model of DS to study systemic metabolic and transcriptional responses in key metabolic tissues to provide a deep understanding of the metabolic changes associated with DS. As part of his work, the authors also aimed to help inform the selection of a mouse model that best reflects the metabolic profile of DS, through comparison with other DS model metabolic data.

The data presented in this model will be of interest to those in the field of metabolism. The immediate impact is unclear, but the breadth of data presented makes this a very useful resource.

Strengths:

(1) This work builds on other comprehensive analyses that the authors have performed in other DS mouse models.

(2) The authors note common metabolic disturbances between male and female mice (e.g., insulin resistance) alongside clearly sexually dimorphic phenotypes (e.g., body weight). Studying both sexes in this context is important.

(3) The authors have written the paper in a way that integrates a large number of observations well. There is complex data, and a high degree of sexual dimorphism. The study has generated a valuable and wide-ranging dataset comprising molecular, biochemical, and physiological data that will be useful for further, more mechanistic studies of metabolism in DS.

(4) For specific observations, like the findings of altered body temperature in male and female mice, the authors undertake follow-up hypothesis-driven analyses of BAT mitochondria and specific hormones. Although these analyses do not explain the change in temperature, they ensure the study is not purely descriptive in nature.

Weaknesses:

(1) Assessing metabolism using dynamic testing is a strength. ITT, GTT and LTTs are included.

(2) The dosing for GTTs, ITTs and LTTs was performed per body weight. But the mice under chow and HFD had different body weights. This may compromise the interpretation of the data. Further, ITTs are presented as percentage change, and this can be heavily influenced by baseline glucose measures. The changes appear quite dramatic, so can the authors plot the raw data instead?

We have updated the ITT data plots to show raw glucose values instead of percentage change. Regarding the dosing, we believe basing it on body weight is an appropriate approach. This method is consistent with nearly all published rodent studies, as blood volume and metabolic tissues such as skeletal muscle and adipose tissue scale with body weight. Adjusting for weight prevents potentially erroneous conclusions. As for the diet groups, we compared WT and Dp16 mice only within the same diet group (Chow or HFD) rather than across different diets. We believe this ensures a valid and appropriate comparison for our study.

(3) In addition, throughout the manuscript, it is not clear which tissues are the most dominant in disrupting metabolism. The ITT and GTT are composite measures across tissues. Tissue-specific analyses using a clamp technique or isolated tissues may provide more clarity here.

Our data suggest a systemic metabolic deficit across multiple tissues, supported by tolerance tests, pan-tissue transcriptomic analyses, and liver and serum metabolite profiling. This is consistent with the triplication of genes in Down syndrome, several of which have known metabolic roles as highlighted in our discussion. We do not have evidence to support the role of a dominant tissue that contributes to the systemic metabolic dysfunction.

Regarding the suggestion to use a clamp technique, we agree this would effectively determine whether insulin resistance is localized in the liver or skeletal muscle. However, we do not currently have the necessary equipment at Johns Hopkins University to perform these experiments. Conducting this work would require sending separate cohorts of WT and Dp16 male and female mice (on both chow and HFD) to an NIH-funded Mouse Metabolic Phenotyping Centre (MMPC). While we appreciate the value of this approach, we believe such labor-intensive experimentation falls beyond the scope of the present study.

(4) One of the aims of the study was "to help inform the selection of mouse model that best reflects the metabolic profile of DS". The discussion does not contain a comparison between the previous work on different strains and relative to known human data.

We chose not to include a comparison of different mouse models in the "Discussion" section because we previously highlighted the widely used Down syndrome models (Ts65Dn, Tc1, and TcMAC21) and their associated caveats in the "Introduction." Given the significant limitations of those models such as hypermetabolism in TcMAC21 and the presence of 41 triplicated protein-coding genes unrelated to human chromosome 21 we focused our in-depth metabolic analyses on the Dp16 model, which does not share these issues. We felt that restating this information in the "Discussion" would be unnecessarily repetitive.

(5) Data availability. Raw metabolomic data should be made available.

We have uploaded all metabolomics data, along with details regarding sample processing and data analysis, to the Metabolomics Workbench, an NIH-funded public repository. We have updated the "Methods" and "Data Availability" sections of the manuscript to include this information and the corresponding access link.

**Reviewer #3 (Public review):**

Summary:

The article by Chen et al. describes the comprehensive metabolic profiling of DP16 mice, a Down syndrome model that carries a duplicated segment of the mouse chromosome syntenic to human chromosome 21. The authors note that this model is superior to previously used models, based on genetics, as ~65% of the chromosome 21 orthologues. The metabolic phenotypes also appear to be more consistent with those observed in humans with Down Syndrome. The study lays the groundwork for a more detailed genetic dissection of dosage-sensitive genes that contribute to the metabolic deficits observed in Down Syndrome.

Strengths:

There is an enormous amount of data in this manuscript, and the methods are described with adequate attention to detail. A strength of the manuscript is that both male and female mice were analyzed, so that concordant and discordant phenotypes were identified. Both males and females had evidence of insulin resistance. Transcriptomic and metabolomic data revealed impaired pathways for lipid metabolism, a pro-inflammatory state, reduced mitochondrial health and oxidative stress. Although the effects of a high-fat diet on weight gain were divergent, this diet caused worsened insulin resistance in both males and females.

The discussion is excellent. Limitations of the study are well described. This reviewer does not identify any critical missing data.

Weaknesses:

It might have been helpful to have included blood pressure measurements, given the differences in 19-Nor-deoxycorticosterone. The discussion references several articles that describe sex-dependent differences in metabolic phenotypes in humans with Down syndrome, and it might have been helpful to state more explicitly whether these differences correlate with those observed here in mice.

We appreciate the suggestion of blood pressure measurements. While we agree this is an important metric, given the metabolic focus of the present study and the significant volume of data already presented, we feel that blood pressure analysis is beyond the current scope and better suited for a follow-up study.

Our study highlights sex differences in metabolic phenotypes in individuals with Down syndrome. While most published human studies focus on a limited set of parameters such as body weight, adiposity, serum lipoprotein profile, and fasting lipid/glucose levels our mouse data remain generally concordant with these findings. Beyond these standard measurements, we also observed substantial sex differences in pan-tissue transcriptomes as well as serum and liver metabolites.

**Recommendations for the authors:**

**Reviewer #1 (Recommendations for the authors):**

(1) A major question is how these findings compare to data that have previously been published. For example, Lamantia et al. Bone 2024 and Dard et al. European Journal of Pharmacology 2025 both report no changes in body weight using the same Dp(16)1Yey Down syndrome mouse model? There is also a recent publication on liver dysfunction in Down Syndrome using the same mouse model. It would be useful to understand some of the similarities and differences of what is being reported by Dunn et al. Cell Rep 2026. In this assessment, there is an in-serum alanine transaminase (ALT) level, which was not the case in Dunn et al?

For the Lamantia et al. Bone 2024 study, the authors only measured the body weights of Dp16 mice at 6 weeks of age. Our findings at 6 weeks align with Lamantia et al., showing no weight differences between Dp16 and WT mice of either sex (Fig. 2A and C). For the Dard et al. 2025 study, the authors only measured the body weights of Dp16 mice at 12 weeks old (P90) and observed no differences in body weights between genotype of either sex. At 12 weeks of age, we also did not observe body weight differences between Dp16 male mice and WT littermates (Fig. 2A). However, at 12 weeks of age, the Dp16 female mice clearly gained more weight compared to WT littermates (Fig. 2C). Our study tracked weights weekly from 6 to 16 weeks, revealing that while Dp16 females start at weights similar to WT littermates, the groups diverge over time. The reason for the difference between our findings and the single-point measurement by Dard et al. is unclear. Notable variables include:

Mouse Sourcing: We obtained all cohorts and littermate controls from Jackson Laboratory, while Dard et al. bred their mice in-house.

Diet: We used Envigo standard chow (catalogue # 2018SX). Dard et al. did not specify the chow used in their study.

It remains uncertain whether these or other environmental factors contribute to the observed weight differences in female mice.

In the Dunn et al study (Cell Rep 2026), they also performed metabolic analyses on serum and liver tissue in Dp16 mice. Consistent with their metabolic analyses of serum and liver tissue in Dp16 mice, we also observed the upregulation of multiple bile acids, including taurochenodeoxycholic, tauromuricholic, taurolithocholic, and lithocholic acids. Furthermore, our findings align with theirs regarding the transcriptomic and biochemical signatures of hepatic inflammation and fibrosis. However, there are two notable differences between our studies:

(1) Liver Injury Markers: We observed an elevation in serum ALT, whereas the Dunn et al. study did not.

(2) Sex Differences: We identified significant sex differences in the Dp16 transcriptome and metabolome. In contrast, Dunn et al. reported minimal to no sex differences and consequently combined male and female data for all analyses.

Because Dunn et al. combined male and female data, a sex-stratified comparison between our results (separated by sex) and theirs was not feasible.

(2) It would be important to understand trends in wild-type animals compared to Dp16 mice. For example, the sex specific and non-specific features - are any of these described in obesogenic wild-type animals fed on a high-fat diet? I.e., are the same features at play and just exacerbated in Dp16, or is this a Dp16-specific feature of systemic metabolism?

Published literature indicates that WT females typically gain significantly less weight on a high-fat diet (HFD) than WT males. However, our data suggest that the weight gain patterns observed in Figure 6A and C are specific to the Dp16 genotype. Dp16 females gained substantially more weight during the first six weeks of HFD before WT females caught up. In contrast, Dp16 males showed robust initial weight gain comparable to WT controls, but their weight plateaued after seven weeks while WT controls continued to gain, leading to a clear divergence (Fig. 6A).

Other metabolic parameters also appear specific to the Dp16 model. On a standard chow diet, WT mice of both sexes generally do not exhibit glucose intolerance, insulin resistance, dysregulated lipoprotein profiles (VLDL-TG), or an impaired capacity to handle lipid loads. We observed all of these features in our Dp16 male and female mice (Fig. 3). Furthermore, transcriptomic analyses of Dp16 mice on standard chow revealed gene signatures of inflammation, fibrosis, and oxidative stress that are absent in WT mice.

When challenged with HFD, while WT mice typically develop glucose intolerance and insulin resistance, the triplicated genes in Dp16 mice significantly exacerbated this metabolic deterioration. This is reflected in the worsening of glucose control and insulin sensitivity observed in our tolerance tests.

In summary, most of these metabolic features are specific to Dp16 mice on a standard chow diet and are further exacerbated when combined with a high-fat diet.

(3) Food intake data is difficult to interpret when weight has already diverged, as bigger animals will eat more food. Hence, the higher food may be a consequence rather than a cause of the weight gain (data in Figure 1).

The reviewer makes a valid point. Since physical activity and energy expenditure do not differ significantly between Dp16 females and WT controls (Fig. 2F), the observed increase in food intake may indeed contribute to the higher body weights in Dp16 female mice.

To rigorously confirm this, food intake would need to be measured between 6 and 8 weeks of age, prior to the divergence in body weight. Unfortunately, we did not measure food intake at that earlier time point.

(4) The n numbers seem to vary significantly. For example, the use of n=6 for metabolic studies is generally rather small and underpowered. For the seahorse data, another concern is the snap freezing of samples before Seahorse assessment. For example, snap freezing of samples has been shown to increase certain metabolites. Freeze-thaw tissues often show a significant reduction in optical redox ratio.

Regarding the transcriptomics and metabolomics studies, we utilized *N*=6 mice per tissue per sex. While we agree that a larger sample size is always preferable, the high cost of OMICS analyses covering 144 RNA-seq and 48 metabolomics samples limited our capacity to increase this number. However, *N*=6 remains a robust and standard approach for these specific assays. For the majority of our other in vivo and ex vivo data, we employed a higher sample size of 12-15 mice per genotype per sex to ensure statistical rigour. For a few assays, we have sample size of over 20.

Regarding the respirometry analysis, we acknowledge the limitations of using frozen tissue. We chose this method because it allowed us to perform Seahorse assays on multiple tissues from 9-10 mice, which is a significant sample size for this type of analysis. The alternative isolating mitochondria from fresh tissue would have restricted our ability to process multiple tissues from a large number of animals on the same day due to the length of the protocol. We believe this trade-off was necessary to maintain a high sample size across various tissues.

(5) For oestradiol measurements, were the samples taken at the same times within the estrous cycle? This may affect the comparability of female Dp16 and WT mice?

Regarding our protocol, blood samples were collected between 11:00 AM and noon, with food removed two hours prior. While we did not specifically monitor the oestrous cycle of the female mice, serum samples for both the Dp16 females and WT littermates were collected on the same day and at the same time to ensure comparability across the groups.

(6) Body weight reduction and organ size reduction on an HFD are especially interesting. Could enhanced inflammation and fibrosis be the root cause of this? Are there other mouse models where this is the reason?

On a high-fat diet, we observed a reduction in iWAT and gWAT fat depot weights in both male and female Dp16 mice, which is consistent with their lower overall body weights (Fig. 6 - figure supplement 3). Conversely, Dp16 females fed a high-fat diet showed increased heart and kidney weights. Despite their lower adiposity, the Dp16 mice on this diet exhibited greater insulin resistance and glucose intolerance (Fig. 7). This suggests that the worsening of glucose control is independent of obesity. While we observed signatures of inflammation and fibrosis, we do not yet have direct mechanistic evidence demonstrating that these factors causally impaired glucose and lipid metabolism.

(7) The authors are circumspect throughout to avoid over-claiming, as the majority of data is observational. One exception: "Many bile acids serve as ligands for nuclear hormone receptors (e.g., FRX and TGR5) that control various aspects of glucose and lipid metabolism (74, 75), and extensive changes in circulating bile acids are contributing, at least in part, to the systemic metabolic phenotypes in Dp16 mice." The authors have not shown a direct link between bile acids and metabolism in this model. Please edit.

We have edited the text accordingly.

Minor:

(1)"Most human studies at the whole-body level are limited to assessing the impact of trisomy 21 on food intake, adiposity, physical activity level, and energy expenditure in adolescents or adults with DS"

While we were uncertain of the reviewer's specific intent regarding the suggested changes, we have rephrased the sentence for clarity.

(2) It is somewhat surprising that T3 is elevated, although there are reports of T3 elevation in visceral obesity in humans (e.g., Sun Nam et al., Obes Res Clin Pract, 2010).

We observed that T3 levels did not differ by genotype in mice of either sex when fed a standard chow (Fig. 2 - figure supplement 5). However, we noted elevated T3 levels in both male and female Dp16 mice on a high-fat diet (Fig. 6 - figure supplement 2). While increased T3 levels correlated with higher physical activity and a modest increase in metabolic rate in Dp16 females, this was not observed in males (Fig. 6). We do not currently have a clear explanation for these findings. Given that individuals with Down syndrome often present with hypothyroidism and lower T3 levels, this discrepancy may reflect a species-specific difference between humans and mice.

(3) Please can the authors clarify the percentage gene coverage, as this is quoted as ~58% of Hsa21 gene orthologs or ~65% of the Hsa21 gene orthologs, where the same reference is used.

We apologize for the confusion. The number of triplicated genes in Dp16 mice corresponds to ~58% of Hsa21 genes (PMID: 26765563). We have corrected the typographical error in the text.

(4) "segmental duplication model carrying a majority of the triplicated Hsa21 gene orthologs" for this given percentage majority sounds too strong, and the use of percentage is recommended.

We have modified the text accordingly.

(5) It is puzzling that in female gWAT with 7 triplicated Hsa21 gene orthologs (Rbm11, Chodl, Cldn8, Sh3bgr, Igsf5, Itgb2l, and Tmprss2). Could this be a technical issue? Was the reduced expression quantified by RT-Q-PCR?

We have examined the normalized counts in the RNA-seq data for the seven genes in question, and the results do not appear to be an artifact. The sample size for this data is six mice per tissue per sex. In general, we prefer utilizing raw and normalized counts from RNA sequencing because there is a linear relationship between transcript amount and raw counts that is independent of housekeeping genes. In contrast, RT-qPCR involves mRNA amplification and requires expression to be normalized by one or more housekeeping genes (such as GAPDH, β-actin, 36B4, or ubiquitin) under the assumption that their levels remain constant.

(6) The difference in body temperature is of interest. In male Dp16 mice, there is an increase in core temperature and a lowering of body temperature in females. In female Dp16 mice, higher estradiol levels have been stated by the authors to contribute to lower body temperature and higher physical activity (69-72). I am uncertain if the references are all relevant, as some relate to ovariectomized animals. No explanation is given for males.

We currently do not have an explanation for why Dp16 males on a chow diet exhibit higher core body temperature, while Dp16 females show lower body temperatures. Although elevated T3 levels can increase body temperature, we have ruled this out; our data indicates there are no significant differences in T3 levels between genotypes for either sex on a chow diet.

(7) The authors find a higher percentage heart weight in Dp16 mice on HFD and comment in the discussion that this is in keeping with "high-fat diet-induced cardiac hypertrophy". From what I can see, no histology has been performed to justify this statement. Furthermore, it would be useful to understand which animals had congenital heart disease in the first instance.

We have modified the text accordingly. Unfortunately, we do not have histology data on the heart to inform us on whether some of our mice had congenital heart disease.

**Reviewer #2 (Recommendations for the authors):**

(1) The authors should comment on the dosing method of glucose/insulin/lipid in the tolerance tests to acknowledge that differences in body weight may affect these tests. In addition, I encourage the authors to present ITT data as raw data, and not % change.

In response to the reviewer’s comments, we have updated the ITT data plots to show raw data rather than percentage change. Regarding the dosing methodology, we maintain that basing dosage on body weight is appropriate. This approach is consistent with the vast majority of published rodent studies, as blood volume and metabolic tissues—such as skeletal muscle and adipose tissue—scale with body weight. Standardizing dose independently of body weight could lead to erroneous conclusions.

(2) It would be useful for the authors to include a discussion on the likely specific tissue involvement in the whole-body metabolic disturbance. From my reading of the manuscript, there seems to be data suggesting functional and transcriptional dysfunction across most tissues, but do the authors suggest there is a dominant tissue in this regard?

Due to the triplication of large number of genes on human chromosome 21, people with Down syndrome exhibit deficits across most organ systems (PMID: 32029743). Metabolic homeostasis also involves multiple tissues and cell types (adipose tissues, liver, skeletal muscle, pancreas, gut, hypothalamus, and immune cells). Most of the triplicated genes do express across these tissues. Our data indicate metabolic dysregulation across adipose tissues (white and brown), liver, skeletal muscle, and hypothalamus. Given the complex genetic perturbations of the Down syndrome mouse model, we do not think that there is a dominant tissue that contributes disproportionately to the systemic metabolic dysfunction phenotypes we observed in the Dp16 mice. Rather, we think that the metabolic phenotype is due to the combined deficits across multiple organs and tissues. As we do not have data to support the disproportionate contribution of any one tissue, we therefore did not speculate on the dominant contribution of any single tissue in the Discussion.

(3) Related to this, muscle lipid is thought to be a major driver of muscle insulin resistance. Do the authors have measures of muscle lipid accumulation? This might be particularly interesting in the HFD models.

Unfortunately, we did not measure lipid content in the skeletal muscle during this study. For the chow-fed mice, the entire gastrocnemius muscle was used for RNA isolation to perform RNA sequencing, and no tissue remains for additional analysis. Regarding the HFD-fed group, skeletal muscle was not collected at the termination of the study. As a result, we are unable to provide the requested lipid analysis data.

(4) For mitochondrial analyses - do the authors have measures of total tissue mitochondria, and might changes in mitochondria abundance be driving some of these differences?

For all our mitochondrial respiration analyses, we normalized the data to mitochondrial content as quantified by the MTDR assay (PMID: 32432379; PMID: 39704485). These results indicate that for a given amount of mitochondrial content, respiration as measured by the Seahorse assay is reduced in Dp16 mouse tissues, specifically in the BAT and liver.

(5) To broaden the scope and interest, can the authors compare the transcriptional or metabolomic data to what has been found in non-DS insulin resistance (humans or mice), for example? This may help to highlight the key changes in metabolism that are causal for specific phenotypes.

Overall, this is a comprehensive assessment of metabolism in a DS model.

We appreciate the reviewer’s suggestion. However, given the vast number of published datasets on non-DS insulin resistance in both humans and mice, comparisons would yield varying results depending on the specific datasets selected. Consequently, we feel that such an analysis is beyond the scope of this study. We would like to highlight that many of the processes dysregulated in Dp16 mice as identified through our pan-tissue transcriptomes and metabolomes align with those frequently observed in non-DS insulin resistance. These include signatures of chronic low-grade inflammation, fibrosis, ER and oxidative stress, and impaired glucose and lipid metabolism.

**Reviewer #3 (Recommendations for the authors):**

It is slightly disconcerting that Figure 5 - Figure Supplements 2-5 are referred to in the text before the data in Figure 5 are discussed. It might make sense to indicate that the data are discussed further below (assuming that the authors do not wish to renumber these figures).

We have fixed this issue raised by the reviewer.
